# Outdoor risky play and health-related outcomes in children aged 3–12 years: A mixed-methods systematic review

**DOI:** 10.1371/journal.pone.0347444

**Published:** 2026-10-26

**Authors:** Rachel Ramsden, Alethea G. Jerebine, Gemma Brailey, Ellen Beate Hansen Sandseter, Hebah Hussaina, Avril Johnstone, Bryony Longdon, G. J. Melendez-Torres, Rachel Nesbit, Mariana Brussoni, Helen F. Dodd

**Affiliations:** 1 Department of Pediatrics, The University of British Columbia, Vancouver, British Columbia, Canada; 2 British Columbia Children’s Hospital Research Institute, Vancouver, British Columbia, Canada; 3 Human Early Learning Partnership, School of Population and Public Health, University of British Columbia, British Columbia, Vancouver, Canada; 4 Deakin Institute for Physical Activity and Nutrition, School of Health and Social Development, Deakin University, Melbourne, Victoria, Australia; 5 Faculty of Health and Life Sciences, University of Exeter, Exeter, United Kingdom; 6 Queen Maud University College of Early Childhood Education, Trondheim, Norway; 7 School of Health and Wellbeing, University of Glasgow, Glasgow, United Kingdom; University of Stirling, UNITED KINGDOM OF GREAT BRITAIN AND NORTHERN IRELAND

## Abstract

**Background:**

Outdoor risky play and its relationship with children’s health, development and well-being have garnered significant attention in recent years.

**Objective:**

This systematic review synthesised existing evidence on associations between children’s outdoor risky play and health outcomes.

**Methods:**

A systematic search of five electronic databases (SportDiscus, MEDLINE, PsychInfo, ERIC, and CINAHL) was initially conducted in December 2023 and updated three times (April 2024, March 2025, May 2026) during the review development. Studies were eligible if they examined outdoor risky play in children aged 3–12 years and assessed a health outcome. Studies that did not use empirical data or did not have an accessible full-text were excluded. Five reviewers independently screened all articles and two separate reviewers resolved discrepancies. Quantitative narrative synthesis and qualitative framework synthesis were completed, and a mixed-methods synthesis occurred using a Pillar Integration Process.

**Results:**

A total of 74 studies met the eligibility criteria for inclusion (48 quantitative; 19 qualitative; 7 mixed-methods). Six health domains (physical health, mental health and well-being, aggression, social, cognitive, and risk competence and injury) and four risky play exposures were identified: general risky play (n = 13 studies); environments that support risky play (n = 21); independent mobility (n = 19); and rough-and-tumble play (n = 21). General risky play and risky play supportive environments were positively associated with health outcomes across physical, mental health and well-being, social, cognitive, and risk competence domains. Independent mobility was positively associated with physical activity, with insufficient evidence to determine associations for other domains. Evidence for rough-and-tumble play was inconsistent across all domains, except for a negative association with aggression (i.e., increased aggression). The overall quality of evidence was low-to-moderate.

**Conclusion:**

Outdoor risky play shows promising associations with children's health outcomes. The low number of studies in some health domains and need for higher quality evidence preclude firm conclusions and provide direction for further research in this field.

**Registration:**

International Prospective Register of Systematic Reviews (PROSPERO) (No. CRD42023488023).

## Introduction

Risky play is a thrilling and exciting form of free play that involves elements of uncertainty and the possibility of physical injury [1]. It encompasses activities such as climbing to heights, moving at high speeds, using tools, playing near potentially dangerous elements like water or fire, engaging in rough-and-tumble (R&T) play, exploring unfamiliar environments without adult supervision, and experiencing the thrill of risky play vicariously by observing others [1,2]. Alternative terms to describe play that involves elements of risk [3] include ‘outdoor play,’ ‘nature play,’ ‘active play,’ ‘adventurous play,’ and ‘free’ or ‘unstructured’ play [4]. While the terminology may differ across geographic regions and sectors, it is well recognised that some degree of risk is inherently present when children are given the freedom to engage in self-directed play. Importantly, a clear distinction must be made between ‘risk,’ which entails situations that children can perceive and evaluate, and ‘hazard,’ which involves dangers that surpass a child's developmental capacity to recognise or manage effectively [3]. Children actively and willingly seek out situations based on their subjective perception of danger and uncertainty [5]. For them, the excitement of a thrilling experience and the satisfaction of mastering increasingly difficult challenges often outweighs the perceived risks or potential negative outcomes [6]. Risky play is predominantly associated with outdoor or natural environments, as these settings provide a unique combination of affordances that support and stimulate children's engagement with challenge, exploration, and risk [7,8], for example through climbing trees, exploring uneven terrain, or building makeshift structures.

Amidst the prevalence of screen use and structured activities, risky play offers invaluable opportunities for children to immerse themselves in outdoor activities that afford challenge. Risky play has emerged as a distinct category of play due to its unique developmental benefits for children and its diminishing presence in children's everyday play experiences [9,10]. This can be viewed as a response to increasingly restrictive safety regulations and policies, as well as societal safety-first attitudes, that have been widely implemented and embraced in recent years [11]. Thus, while it is natural for children to participate in risk-associated activities during play, restrictive environments and practices reduce opportunities for risky play [12].

The systematic review conducted in 2015 by Brussoni et al. [13] was the first to synthesise evidence on child health outcomes related to risky play. This review included 18 eligible studies and highlighted a range of positive associations between outdoor risky play and children’s health, including physical activity (PA) and social competence. Drawing on Sandseter’s six categories of risky play [1,14], the review found that independent mobility (IM), as well as environments that support risky play, were positively linked to PA and social health, while being negatively associated with sedentary behaviour. Play at heights showed no correlation with bone fracture frequency or severity. Additionally, R&T play was not linked to increased aggression and was positively associated with higher social competence for boys and popular children, although the results were mixed for other children. Furthermore, qualitative evidence indicated risky play supportive environments increased playtime, and improved social interactions, creativity, and resilience. Of importance, this systematic review demonstrated that the overall health benefits of increased outdoor risky play outweighed the potential health risks associated with such play. The quality of evidence in this landmark review ranged from ‘very low’ to ‘moderate,’ revealing the need for more high-quality studies in this area.

Since Brussoni et al.’s systematic review [13], research examining children's risky play has expanded, alongside increased availability of education, training, and resources on facilitating risky play, and policy changes aimed at promoting risky play, such as updated safety guidelines and advocacy for unstructured and risky play [9,15–18]. Despite this, much of the evidence has relied predominantly on quantitative approaches that treat risky play as a singular, undifferentiated exposure, and have not accounted for the diversity of ways risky play has been conceptualised and operationalised across disciplines. Critically, quantitative approaches alone cannot capture the lived experiences of children and caregivers that are essential for understanding not just whether children’s risky play influences health outcomes, but how and why. There is also a need to synthesise evidence across the diverse ways in which risky play transpires, including environments that support risky play and specific types of risky play, such as IM and R&T play, to enable a more nuanced and comprehensive appraisal of the evidence. The purpose of this mixed-methods systematic review is twofold: to provide an up-to-date synthesis of quantitative and qualitative evidence on the relationship between outdoor risky play and health outcomes in children aged 3–12 years; and to map findings across risky play exposure domains in order to identify where evidence is consistent and convergent, where it is emerging or uncertain, and where significant evidence gaps remain.

## Methods

The approach for the current review was informed by the Brussoni et al. [13] review, but did not replicate it as progress in the field necessitated adaptations, as outlined in the subsequent sections. This current systematic review was registered with the International Prospective Register of Systematic Reviews (PROSPERO) (No. CRD42023488023) on December 6, 2023. The methodology, encompassing both the search strategy and the analysis process, adhered to the Preferred Reporting Items for Systematic Reviews and Meta-analyses (PRISMA) statement for systematic reviews [19,20]. Ethical approval was not required for this study, as it used secondary data and did not involve human participants or identifiable data.

### Study inclusion and exclusion criteria

The inclusion and exclusion criteria for this systematic review are outlined in Table 1. We included studies with a sample mean age between 3.00 and 12.99 years; where school level of participants (i.e., grade or school year) was outlined rather than the age, the standard regional age range for the identified grade level was used. For studies following participants over time (prospectively or retrospectively), child participant age at the time at which the risky play exposure occurred was used to determine eligibility. To assess the association between risky play and health outcomes, some variance in risky play is required, therefore quantitative studies were required to include a less risky or non-risky play behavioural or environmental comparison (including internal comparison) or control. Studies with continuous measures of risky play (without a formal comparison group) were eligible if there was an inferential analysis of the relationship between risky play and health. Non-English full-texts were translated and assessed for inclusion.

**Table 1:** Systematic review inclusion and exclusion criteria.

Criteria	Inclusion	Exclusion
*Study type*	Quantitative, qualitative and mixed-method studies that use empirical methods of data collection.	Studies that do not use empirical data collection methods (e.g., reviews, opinion pieces, editorials).
*Language and availability*	Full-text is available.	Full-text not available or translation not possible of full-text in non-English language.
*Population*	Mean age of participants must be between 3 years and 12.99 years. Oldest participant must be ≤ 18 years. Include teacher/ practitioner/parent perspectives if describing effect of risky play intervention/activity on children (within given age range).	Mean age of participants is < 3 years or > 12.99 years. Adults’ retrospective reflection on the effects of their childhood play. General adult perspectives of play/risky play (e.g., effect without a measured activity/intervention). Exclude animal studies.
*Exposure (measure of risky play)*	Objective and/or subjective measures of risky play are included OR environments that support/afford risky play are studied.	Risky play not measured and/or environments that afford risky play not studied. Studies examining indoor play exclusively. Studies examining environments where risky play was not specified as occurring or it is unclear if the environment included hazards. Therapeutic play interventions for clinical conditions. Exposure is not playful (intent to harm; or not reciprocated/consented by all children/participants). Risky play exposure is not the primary exposure of interest but an incidental or confounding variable.
*Comparison group*	Quantitative studies that include a less risky or non-risky play behavioural or environmental comparison (including internal comparison) or control.	Quantitative studies reporting only descriptive data with no inferential analysis of the relationship between risky play and health.
*Outcome*	Health- or development-related outcome.	Outcome is not health, development or well-being related. Studies where risky play/play is the outcome.
*Outcome (if measure of injury risk)*	Outline risk of injury (including denominator) and a relative risk of injury could be calculated.	Studies related to risk of injury if they do not state how many children were exposed to the risk (such that relative risk of injury could be determined). Studies reporting only injury incidence without a behavioural or environmental risky play exposure.

### Risky play exposure and health outcome

This review utilized the definition of risky play as defined by Sandseter and Kennair [21], where risky play is ‘thrilling and exciting forms of physical play that involve uncertainty and a risk of physical injury.’ This risk can be real or perceived, supporting children to experience excitement, thrill and a level of fear [1,22]. Specific categories of risky play behaviours (‘risky play subtypes’), as defined by Sandseter [1,14], were included as exposures. IM, defined as the extent to which children can travel and play in public spaces without adult supervision [23] was categorised as a subtype of risky play (disappear/ get lost). Studies that examined environments or affordances that support risky play were also included, as outlined in S1 Appendix. Health as an outcome in this review was guided by the World Health Organization’s definition of health and well-being, which describes health as ‘a state of complete physical, mental, and social well-being and not merely the absence of disease or infirmity’ [24].

### Information sources and search strategy

The search was conducted in SportDiscus (EBSCO), MEDLINE (OVID), PsychInfo (OVID), ERIC (EBSCO), and CINAHL (EBSCO). The five databases were selected to capture literature across the key disciplines relevant to children's risky play and health, including sport and exercise science (SportDiscus), medicine and public health (MEDLINE, CINAHL), psychology (PsychInfo), and education (ERIC), were considered most likely to index the interdisciplinary literature relevant to this review's scope, and were used in the 2015 review by Brussoni et al. [13]. Supplementary search strategies (network call; citation searches) were employed in part to mitigate the risk of missing relevant literature not captured by the five databases. Initial search terms were developed by HD and tested by preliminary scoping searches to refine the search terms. The search terms used for the systematic searches were finalized by HD and MB to identify papers focused on our exposure of interest only (risky play, including IM); no search terms were included for health-related outcomes given the breadth of terms that would be required to capture all relevant outcomes. A primary set of search terms were generated, with papers including these terms in their title, abstract or keywords included in the screening phase. The initial search was conducted on December 6, 2023 and the search was re-run on April 11, 2024, March 20, 2025 and May 19, 2026. The full search terms and strategy can be found in S1 Appendix. Through the review process, supplementary search strategies were used. References from key publications on children’s risky play were examined and a network call was conducted through social media and email in April 2024. The network call involved outreach to established professional network of researchers and practitioners working in the field of children's risky play to share studies meeting the eligibility criteria. All submissions were subject to the same screening and eligibility criteria as database-identified studies. While this approach aimed to reduce publication bias, the network was not exhaustive and may reflect geographic or disciplinary concentrations within the research team's professional connections.

### Selection and data collection process

References were imported into Covidence systematic review software [25] and duplicates were removed. Five independent reviewers (RR, HH, GB, BL, AJ) independently screened titles and abstracts, and full text articles (RR, HH, GB, AJ, RN) in Covidence. In instances where reviewers disagreed, they were resolved by HD and MB. Consensus was achieved for all eligibility decisions. Full-text articles that were not published in English (n = 2) were translated for assessment. Data extraction was completed in Covidence by two reviewers (HH, GB) and checked by RR and RN. Development of the data extraction form was guided by best practice guidelines for data extraction, as per Taylor et al. [26].

### Quality assessment

The quality of evidence for all studies was assessed by four reviewers (HH, GB, RR, AGJ) using the Mixed Methods Appraisal Tool (MMAT, 2018 version) [27]. Each study was assessed using the five criteria relevant to its design (qualitative, quantitative, or mixed-methods). Individual studies were not assigned numerical scores; instead, they were rated as low, moderate, or high quality based on the MMAT criteria and narrative assessments of methodological strengths and limitations. No studies were discarded due to low quality, instead, the quality assessment was used to assess the potential influence of the study design, and relevant approaches, on the derived results for each outcome. Study quality ratings were integrated into the mixed-methods synthesis through narrative interpretation rather than formal statistical weighting, given the heterogeneity of study designs and consistent with best practice guidance recommending that methodological quality inform the interpretation and weighting of findings during synthesis rather than determine study inclusion [27–29].

### Data synthesis

To examine the association between outdoor risky play and children’s health, quantitative and qualitative data were analysed separately using quantitative narrative synthesis and framework synthesis, respectively. Given the wide range of study designs, exposure and outcome categories, it was not feasible to conduct a meta-analysis of the quantitative data. The quality of evidence, as assessed by the MMAT, was integrated into the mixed-methods synthesis. All qualitative coding and framework synthesis were conducted in NVivo (version 15) [30]. Quantitative narrative synthesis and vote counting were conducted manually, with frequency plots generated in R. Missing data were not imputed; where studies reported incomplete outcome data, this was noted in the narrative synthesis and considered in the evidence quality grading. Quantitative and qualitative data were coded as positive, inconsistent or neutral, or negative health outcomes consistent with how they were reported by study authors.

### Quantitative narrative synthesis

The quantitative narrative synthesis was conducted by HD as per the Centre for Reviews and Dissemination guidance [31]. Using the extracted quantitative data, the range of health-related outcomes were initially grouped into broader outcome categories (physical, mental health and well-being, aggression, social, cognitive, and risk competence and injury). The papers were then tabulated within these categories and subsequently grouped by risky play exposure type (leading to four outcome categories: general risky play, environments that support risky play, independent mobility, and rough-and-tumble play) , giving an initial overview of how studies were distributed across exposure and outcome pairings.

An adjusted vote-counting approach was used to capture the direction and consistency of associations within each exposure-outcome pairing. For each paper, the number of quantitative associations between a risky play exposure and a health outcome that had been examined statistically was recorded. Subsequently, the number of those associations that were significant and positive were counted, as well as the number that were significant and negative. Positive associations are defined as those where increased risky play was significantly related to better health outcomes. Negative associations are defined as those where increased risky play was significantly related to poorer health outcomes. Any uncertainties regarding vote counting were discussed with the wider research team until consensus was reached. These vote counts were then used to categorise each paper as showing positive associations, negative associations or no consistent findings overall based on the balance of vote counts, for each health outcome separately. All relevant associations were included in the vote counting approach, thus where a single study reported multiple outcome measures within the same outcome category (e.g., both physical activity and motor development within the physical health domain), the association reported for each outcome measure was treated as a separate vote. These vote counts were then visualised on a frequency plot for each health outcome category separately. As vote counting of associations within studies provides information about inconsistencies within studies, this was integrated into the visualisation. The plots for each health outcome visualise the number of papers that provide evidence of positive or negative associations on health, split by consistency. Studies that reported mixed or conflicting findings were categorised as ‘no consistent evidence’ rather than positive or negative. These plots and the extracted data informed the narrative descriptions of the evidence for each outcome, which were drafted to include consideration of the relationships within and between studies, and discussed within the research team until consensus was reached.

### Qualitative framework synthesis

Qualitative synthesis was conducted by AGJ and RN following the five key stages of framework synthesis: familiarisation, framework selection, indexing, charting, and mapping/interpretation. First, included studies were imported into NVivo (version 15) [30] and read by one reviewer (AGJ) several times for familiarity. Then, a codebook was developed in NVivo to guide the qualitative analysis using the child health outcome framework developed for the synthesis of quantitative studies. Third, qualitative data presented as ‘results’ or ‘findings’ in the included studies were extracted and coded using NVivo. Using the child health outcomes framework and codebook, these data were indexed by one author (AGJ), while a second author (RN) independently coded and indexed a subset of papers (n = 4), which were then discussed to further develop and refine the codebook. Where data did not align with the existing framework, new outcome subcategories were created and defined. Next, codes and labels were charted in tables and reviewed and refined collaboratively. Participant and author quotes from the original studies to illustrate each outcome were selected through discussion between authors (AGJ, HD, MB). Outcome categories and subcategories were mapped through tables and narratives, which formed the basis of the manuscript. To strengthen the trustworthiness of the analysis, the framework synthesis method was applied systematically, and the review team practiced reflexivity through journaling, email correspondence and peer debriefing as the analysis progressed. Two authors (AGJ, RN) engaged in an iterative process of coding, discussion, and theme refinement, with interpretations reviewed and discussed with members of the broader review team (HD, MB, RR).

### Mixed-methods synthesis

To synthesise the quantitative and qualitative results, a Pillar Integration Process (PIP) was undertaken, as outlined by Johnson et al. [29]. This approach integrated quantitative and qualitative data through four iterative stages: listing, matching, checking, and pillar building. To integrate findings, study health outcome categories were listed, matched and checked for convergences and divergences. The pillar building stage consisted of developing health outcome categories that applied to both data strands, and determining the strength and direction of evidence within each derived health outcome category by comparing the quantitative and qualitative results. The quality of evidence (MMAT rating), the strength of evidence (number of studies reporting significant findings) and the direction of the evidence (positive, null, negative) within each data strand (quantitative and qualitative) was graded as high (positive or negative), moderate (positive or negative), low (positive or negative) or insufficient, as recommended by the Agency for Healthcare Research and Quality’s Evidence-based Practice Center program [32]. Insufficient grading reflected either a limited evidence base (<2 studies) or mixed findings within the data strand.

Graded evidence for each data strand was then compared and synthesised to create an overall mixed-methods evidence grade. A high (positive or negative) grade indicated concordant directionality between the two graded (high, moderate, or low) quantitative and qualitative data strands. A moderate (positive or negative) grade indicated one high-graded data strand paired with one insufficient or mixed data strand. A low (positive or negative) grade indicated one moderate- or low- graded data strand paired with one insufficient or mixed data strand. An insufficient grading indicated discordant directionality between the two data strands or insufficient evidence overall.

## Results

### Study selection

A total of 74 studies were included in the review (Fig 1) and are summarized in Table 2. Among included studies, 48 were quantitative, 19 were qualitative, and 7 were mixed-methods. Among the 7 mixed-methods studies, 1 did not contain relevant qualitative data [33] and 2 did not include relevant quantitative data [34,35]. This left, a total of 53 papers that were eligible for quantitative synthesis and 25 papers were eligible for qualitative synthesis.

**Table 2:** Overview of included studies.

Author (year)	Sample	Study Type	Risky Play Exposure	Health Outcome Category					
				Phys	MH	Agg	Social	Cogn	Risk/Inj
Adams et al. (2018) [36]	57 (5–10 years)	Cross-sectional	Risky play environments	■					
Aggio et al. (2017) [37]	13,169 (7 years)	Cross-sectional	IM	■					
Aliyas et al. (2024) [38]	537 (6–12 years & parents)	Cross-sectional	IM	■					
Bauer and Pike (2023) [39]	13 (9–13 years)	Phenomenological	General risky play		♢				♢
Bauer et al. (2024) [40]	16 (mothers of 4–12-year-olds)	Phenomenological	General risky play				♢	♢	♢
Branje et al. (2021) [41]	197 (3–5 years); 27 (ECE)	Mixed: RCT; Qualitative descriptive	Risky play environments	■♢	♢		♢	♢	♢
Brussoni et al. (2017) [42]	45 (2–5 years); 16 (ECE)	Mixed: NR-Exp.; Qualitative descriptive	Risky play environments	■	■♢	■	■♢	♢	♢
Bundy et al. (2009) [43]	12 (5–7 years); 9 (Teachers)	Mixed: NR-Exp.; Qualitative descriptive	Risky play environments	■♢	♢	♢	♢	♢	♢
Bundy et al. (2017) [44]	221 (5–7 years); n = NR (Teachers from 9 schools)	Mixed: RCT; Qualitative descriptive	Risky play environments	♢		♢	■♢	♢	♢
Cho et al. (2023) [45]	862 (3–5 years)	Cross-sectional	General risky play	■					■
Coe (2017) [46]	4 (4–5 years); 1 early childhood educator	Phenomenological	Risky play environments	♢	♢		♢	♢	♢
Colwell and Lindsey (2005) [47]	60 (4–6 years)	Cross-sectional	R&T play				■		
De Meester et al. (2014) [48]	736 (10–12 years)	Cross-sectional	IM	■					
Dodd et al. (2023) [49]	2346 (5–11 years)	Cross-sectional	General risky play		■				
Engelen et al. (2013) [50]	221 (5–7 years)	RCT	Risky play environments	■					
Farmer et al. (2017) [51]	10 principals and administrators	Qualitative descriptive	Risky play environments	♢	♢	♢	♢	♢	♢
Farmer et al. (2017) [52]	840 (6–9 years)	RCT	Risky play environments	■					
Farmer et al. (2017) [53]	840 (6–9 years)	RCT	Risky play environments		■		■		
Finegan et al (1991) [54]	108 (7 years)	Cross-sectional	R&T play		■	■	■	■	
Flanders et al. (2009) [55]	85 father-child (2–6 years) dyads	Cross-sectional	R&T play		■	■			
Flanders et al. (2010) [56]	34 father-child (2–6 years) dyads	Cohort	R&T play		■	■			
Flannigan and Dietze (2018) [57]	27 (4–5 years); 4 (educators)	Qualitative descriptive	Risky play environments	♢	♢	♢	♢	♢	♢
Fliek et al. (2015) [58]	105 (2–6 years & parents)	Cross-sectional	R&T play		■				
Freeman and Robinson (2022) [59]	30 father-child (4–7 years) dyads	Cross-sectional	R&T play					■	
Garcia-Cervantes et al. (2016) [60]	1,376 (8–18 years)	Cross-sectional	IM	■					
Hamamci and Dagal (2022) [61]	127 (age NR)	Cross-sectional	R&T play		■			■	
Hart et al. (1992) [62]	136 (3–5 years)	Cross-sectional	R&T play				■		
Higginbottom et al. (2023) [35]	60 (2–6 years); 15 (ECE)	Mixed: NR-Exp, grounded theory	Risky play environments						♢
Hinchion et al. (2021) [63]	10 (6–8 years)	Ethnography	General risky play	♢	♢		♢	♢	♢
Huang et al. (2024) [64]	330 (8–12 years)	Cross-sectional	IM	■					
Imai et al. (2022) [65]	32 (67.9–74.9 months)	Cross-sectional	General risky play				■		
Kirby et al. (2011) [66]	641 (11 years)	NR-Exp	IM	■					
Larouche et al. (2020) [67]	1,699 (grades 4–6)	Cross-sectional	IM	■					
Larouche et al. (2024) [68]	2,291 parents of children (7–10 years)	Cross-sectional	IM		■				
Lavrysen et al. (2017) [69]	76 (4–6 years)	NR-Exp	General risky play	■	■		■	■	■
Leung and Loo (2017) [70]	281 (6–12 years)	Case report	IM		■				
Lindsey and Colwell (2013) [71]	122 (4–6 years)	NR-Exp	R&T play		■				
Lindsey (2014) [72]	148 (age NR)	Cross-sectional	R&T play				■		
Little (2015) [73]	26 (mothers of 4–5-year-olds)	Phenomenological	General risky play	♢	♢			♢	♢
Marques et al. (2014) [74]	636 (11–12 years)	Cross-sectional	IM	■					
McCormack et al. (2011) [75]	927 (10–12 years)	Cross-sectional	IM	■					
McCormack et al. (2024) [76]	14 (caregivers of 3–11-year-olds)	Phenomenological	Risky play environments	♢	♢			♢	♢
Nelson et al. (2008) [77]	357 (4–5 years)	NR-Exp	R&T play		■	■	■	■	
Nielsen et al. (2010) [78]	417 (5–12 years)	Cross-sectional	Risky play environments	■					
Noonan et al. (2016) [79]	194 (9–10 years)	Cross-sectional	IM	■					
Oliver et al. (2022) [80]	377 (parents of 5–11-year-olds)	Qualitative descriptive	General risky play	♢	♢			♢	♢
Page et al. (2009) [81]	1,307 (10–11 years)	Cross-sectional	IM	■					
Page et al. (2010) [82]	1,307 (10–11 years)	Cross-sectional	IM	■					
Paquette et al. (2003) [83]	468 (parent- child dyads); 75 (fathers); 372 (children)	Cross-sectional	R&T play		■	■			
Pellegrini (1988) [84]	94 (5–9 years)	Cross-sectional	R&T play			■	■		
Pellegrini (1989) [85]	94 (5–9 years)	NR-Exp	R&T play			■	■		
Pellegrini (1991) [86]	94 (5–9 years)	NR-Exp	R&T play				■		
Pellegrini (1993) [87]	42 (10–11 years)	Cross-sectional	R&T play				■		
Pellegrini (1994) [88]	54 (10–13 years)	Cross-sectional	R&T play			■	■		
Pellegrini (1995) [89]	82 (12–13 years)	NR-Exp	R&T play			■	■		
Pellegrini (2003) [90]	138 (median = 12.8 years)	Cross-sectional	R&T play			■			
Pepler et al. (1998) [91]	39 (6–12 years)	Cross-sectional	R&T play		■	■	■		
Rutberg et al. (2025) [92]	43 (9–10 years)	Qualitative descriptive	IM	♢	♢		♢		
Sando et al. (2021) [93]	79 (3–5 years)	Cross-sectional	General risky play	■	■				
Sandseter (2009) [94]	29 (4–5 years)	Ethnography	General risky play	♢	♢	♢			♢
Sandseter (2012) [95]	16 (3–5 years); 7 (ECE)	Phenomenological	General risky play	♢	♢				♢
Schoeppe et al. (2014) [23]	191 (8–13 years)	Cross-sectional	IM	■					
Speldewinde (2025) [96]	220 (4–5 years); 15 (ECE)	Ethnography	Risky play environments	♢	♢			♢	♢
Spencer et al. (2021) [97]	ECE working with children aged 3–5 years (n = NR)	Qualitative descriptive	Risky play environments	♢	♢			♢	♢
Sterman et al. (2020) [98]	27 school staff working with children (5–17 years)	Qualitative descriptive	Risky play environments				♢	♢	♢
StGeorge et al. (2018) [99]	52 parents (mean = 8.4 years)	Phenomenological	R&T play	♢	♢	♢	♢	♢	♢
Stone et al. (2014) [100]	856 (10–12 years)	Cross-sectional	IM	■					
Storli and Lorås (2025) [101]	96 (7–10 years)	Cross-sectional	General risky play	■					
Storli and Hagen (2010) [33]	16 children (3–5 years)	Mixed: NR-Exp	Risky play environments	■					
Traub (2025) [102]	13 adults (directors, teachers and parents)	Qualitative descriptive	Risky play environments	♢	♢			♢	♢
van Rooijen et al. (2023) [103]	475 (4–12 years); 49 (4–10 years)	Phenomenological	Risky play environments	♢	♢	♢	♢	♢	♢
Veiga et al. (2022) [104]	90 (3–5 years)	Cross-sectional	R&T play		■	■			
Weir (2023) [105]	17 (9–10 years)	Phenomenological	IM	♢			♢		♢
Zamani (2016) [34]	22 (qualitative) & 36 (quantitative) children (4–5 years); 4 (ECE)	Mixed: cross-sectional, qualitative descriptive	Risky play environments	♢	♢		♢	♢	♢

ECE = Early Childhood Educator; Exp. = Experimental; IM = Independent Mobility; Mixed = Mixed = Methods; Qual. = Qualitative; RCT = Randomised Control Trial; Phys = Physical health outcome; MH = Mental health and well-being outcome; Social = Social outcome; Agg = Aggression outcome; Cogn = Cognitive outcome; Risk/Inj = risk competence and injury outcome; Phenomenology = qualitative phenomenology; Ethnography = qualitative ethnography; NR-Exp. = Non-randomised experimental; NR = Not reported.

♢ = qualitative outcome; ■ = quantitative outcome.

**Fig 1:**
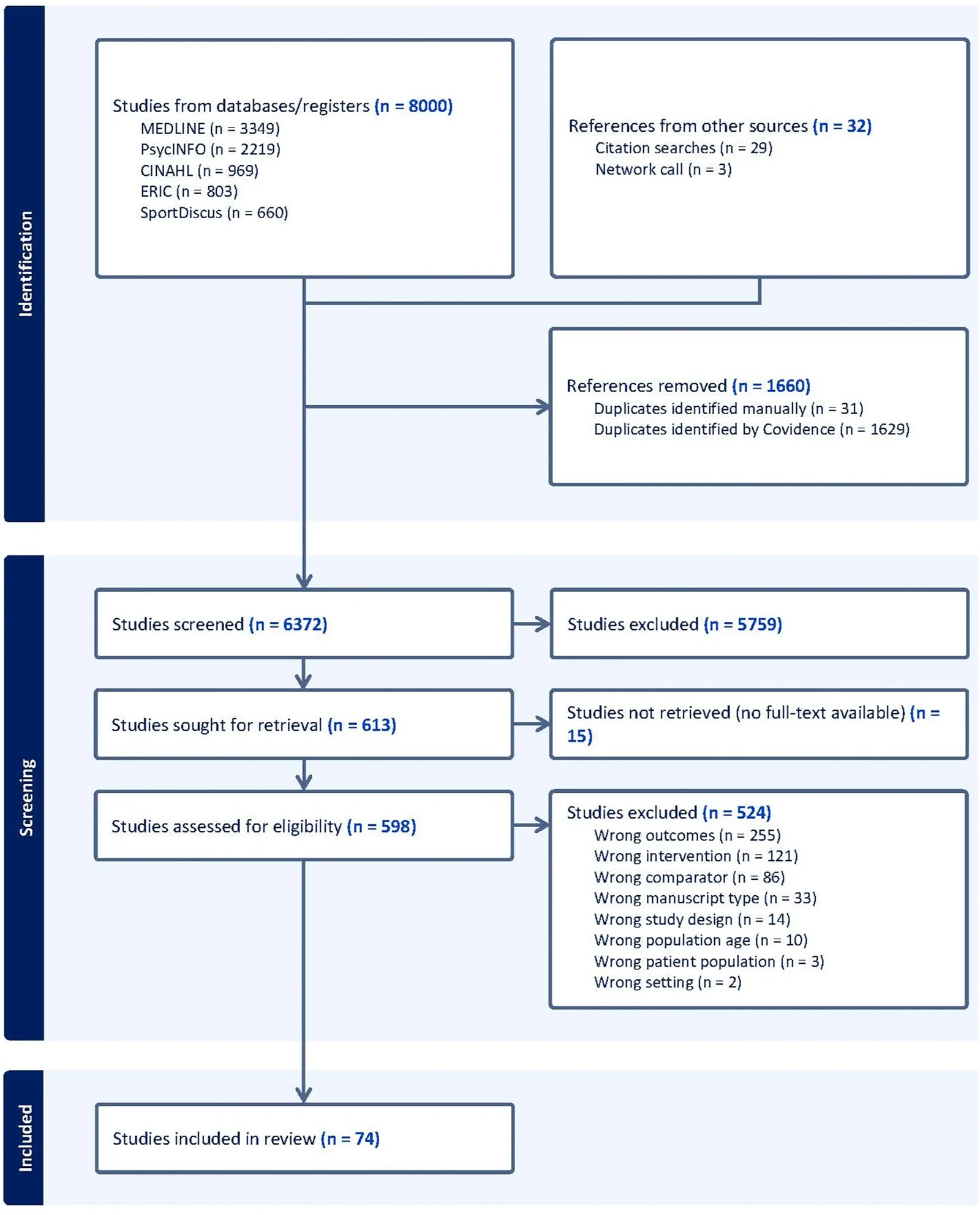
PRISMA diagram for selection of studies included in the systematic review.

### Study characteristics

A full list of papers included in the quantitative and qualitative synthesis, as well as their key study characteristics and relevant findings are presented in S2 Appendix. Studies were published between 1988 and 2025, with over half of eligible studies produced since 2015 (n = 43). Primary countries covered by the studies included Canada (n = 17), the USA (n = 14), Australia (n = 12), UK (n = 9), New Zealand (n = 4) and Norway (n = 5). Most studies included children aged between 3 and 5 years (n = 15) or 6 and 12 years (n = 34). Seven studies included participants over the age of 12 years, with two studies including children aged up to 17 or 18 years [60,98]. Six studies included children under age 3; one study included children aged 1 [83]. Sample sizes ranged from 4 [46] to 13,169 [37]; 26 studies included >200 participants and 38 studies included under 100 participants. The majority of included studies sampled children; 14 studies sampled adults (parent, educator, etc.) on their own and 31 studies sampled adults in conjunction with children. Only 5 studies employed a longitudinal design [55,66,71,86,89] and 5 studies reported on a randomised controlled trial (RCT) [41,44,50,52,53]. Across the quantitative studies, effect sizes were consistently small in magnitude.

Across studies, the most common risky play exposures were R&T play (n = 21) and environments that support risky play (n = 21). The only other risky play subtype specifically investigated was IM (n = 19); thirteen studies examined general risky play (studies where risky play was assessed without a focus on specific subtypes). Final child health outcomes were categorised as follows: physical outcomes (n = 44); mental health and well-being outcomes (n = 36); aggression outcomes (n = 20); social outcomes (n = 29); cognitive outcomes (n = 24); and risk competence and injury outcomes (n = 26) (Table 2). Results are presented by each health outcome category, with findings from the synthesis of quantitative studies and qualitative studies provided sequentially. Evidence synthesis is illustrated by health outcome category for quantitative studies in Figs 2–7, and by health outcome with illustrative quotes for qualitative studies in S3 Appendix.

**Fig 2:**
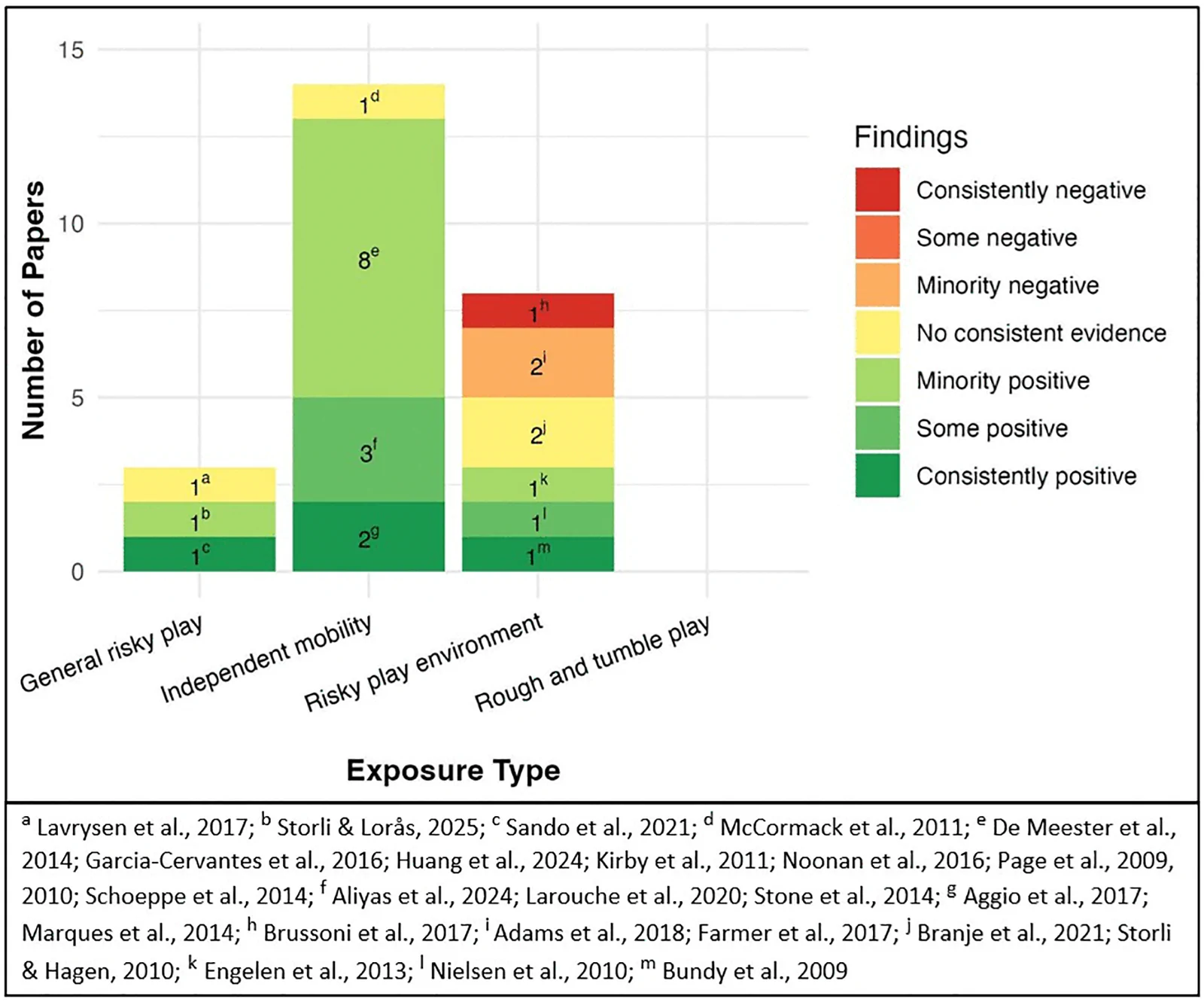
The number of studies reporting quantitative findings between risky play categories and physical outcomes, organised by risky play exposure category.

**Fig 3:**
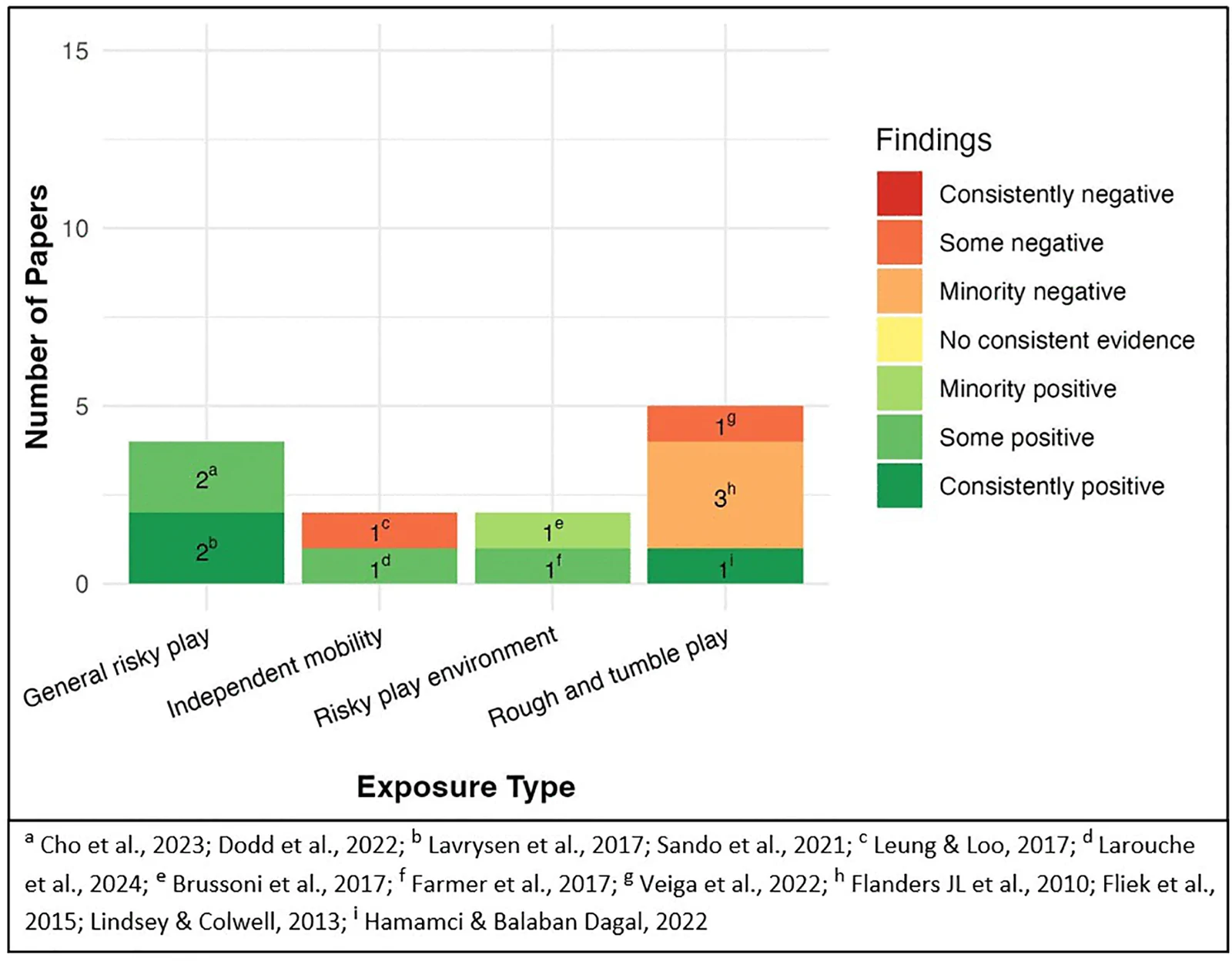
The number of studies reporting quantitative findings between risky play and mental health and well-being outcomes, organised by risky play exposure category.

**Fig 4:**
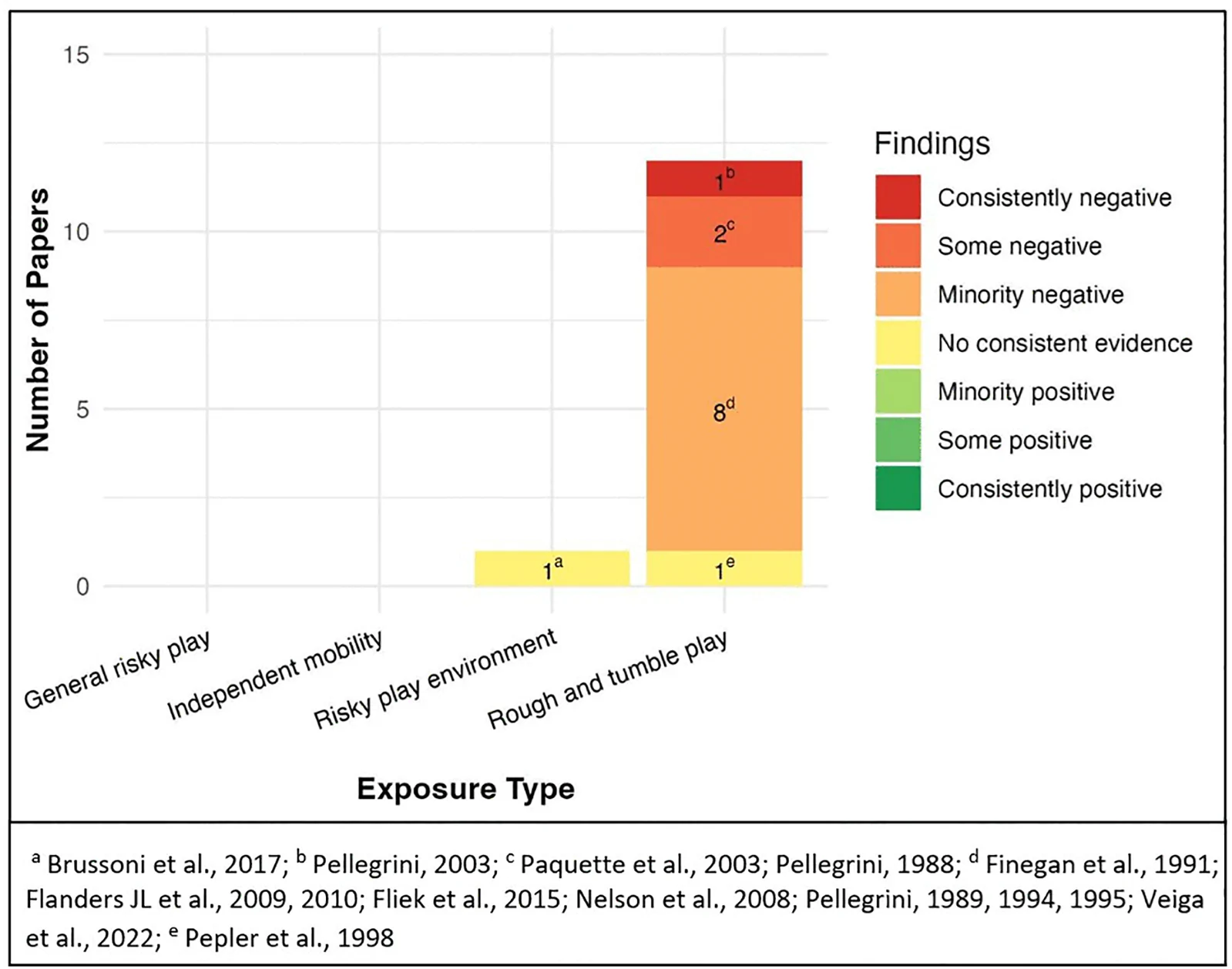
The number of studies reporting quantitative findings between risky play and aggression outcomes, organised by risky play exposure category.

**Fig 5:**
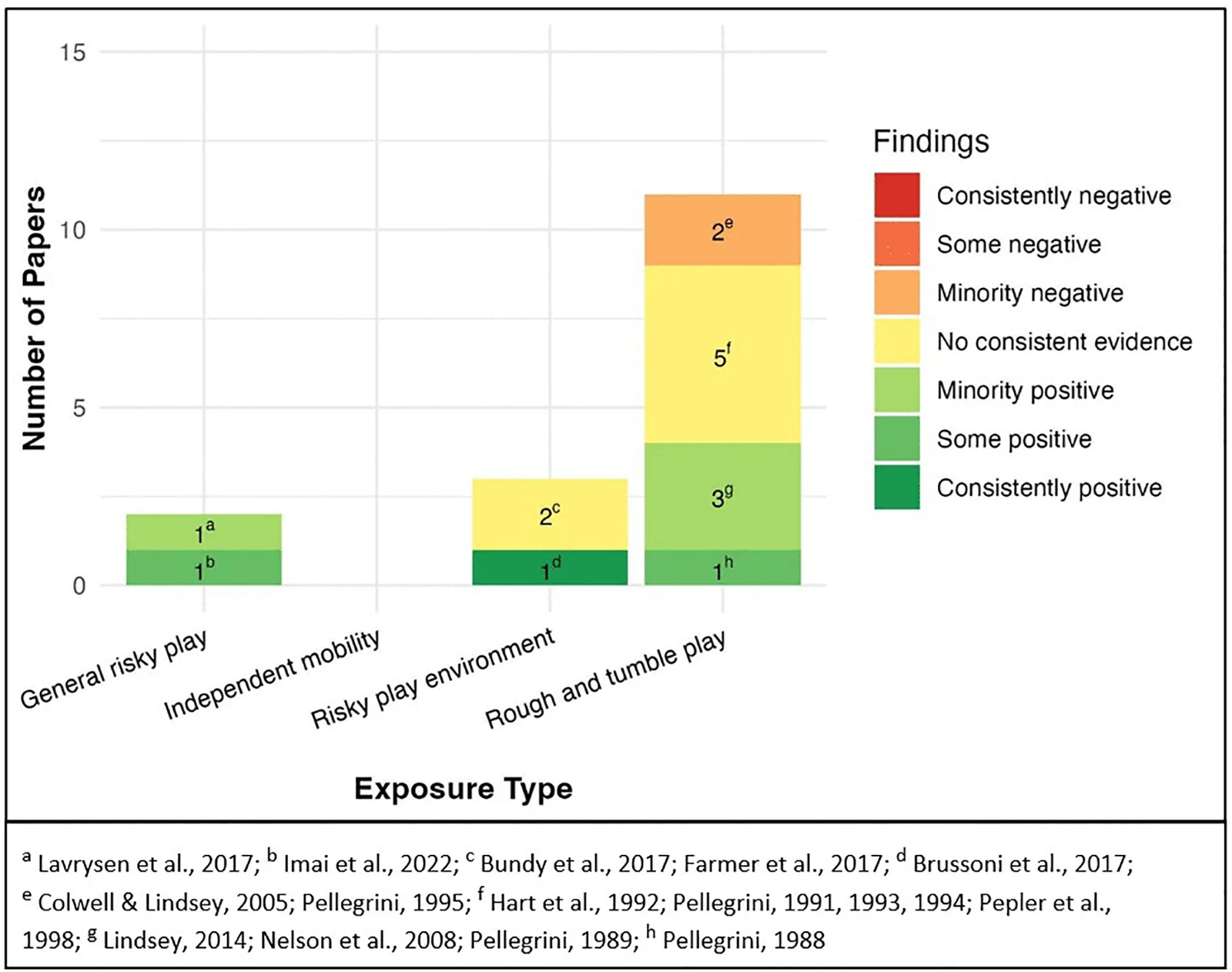
The number of studies reporting quantitative findings between risky play and social outcomes, organised by risky play exposure category.

**Fig 6:**
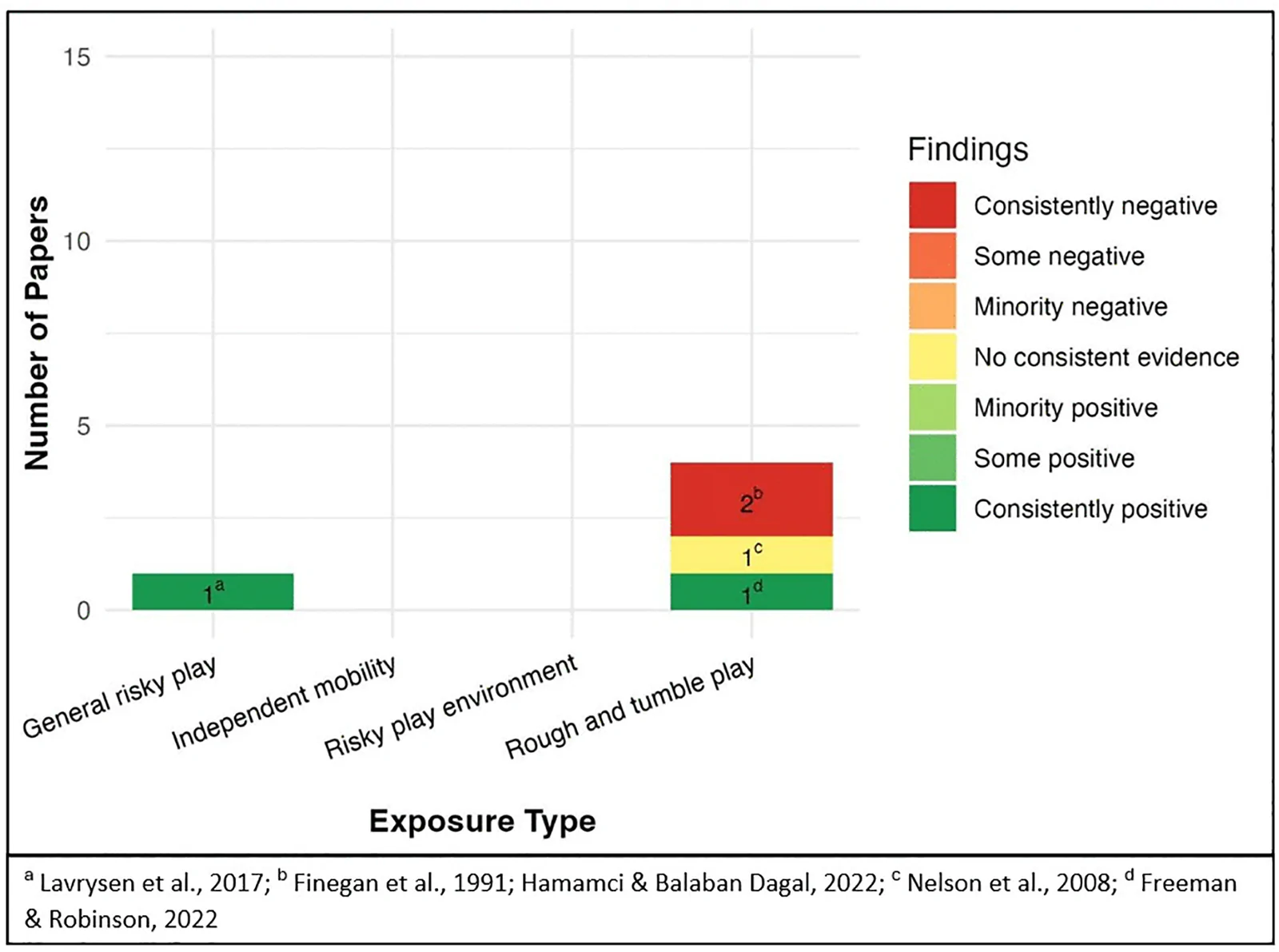
The number of studies reporting quantitative findings between risky play and cognitive outcomes, organised by risky play exposure category.

**Fig 7:**
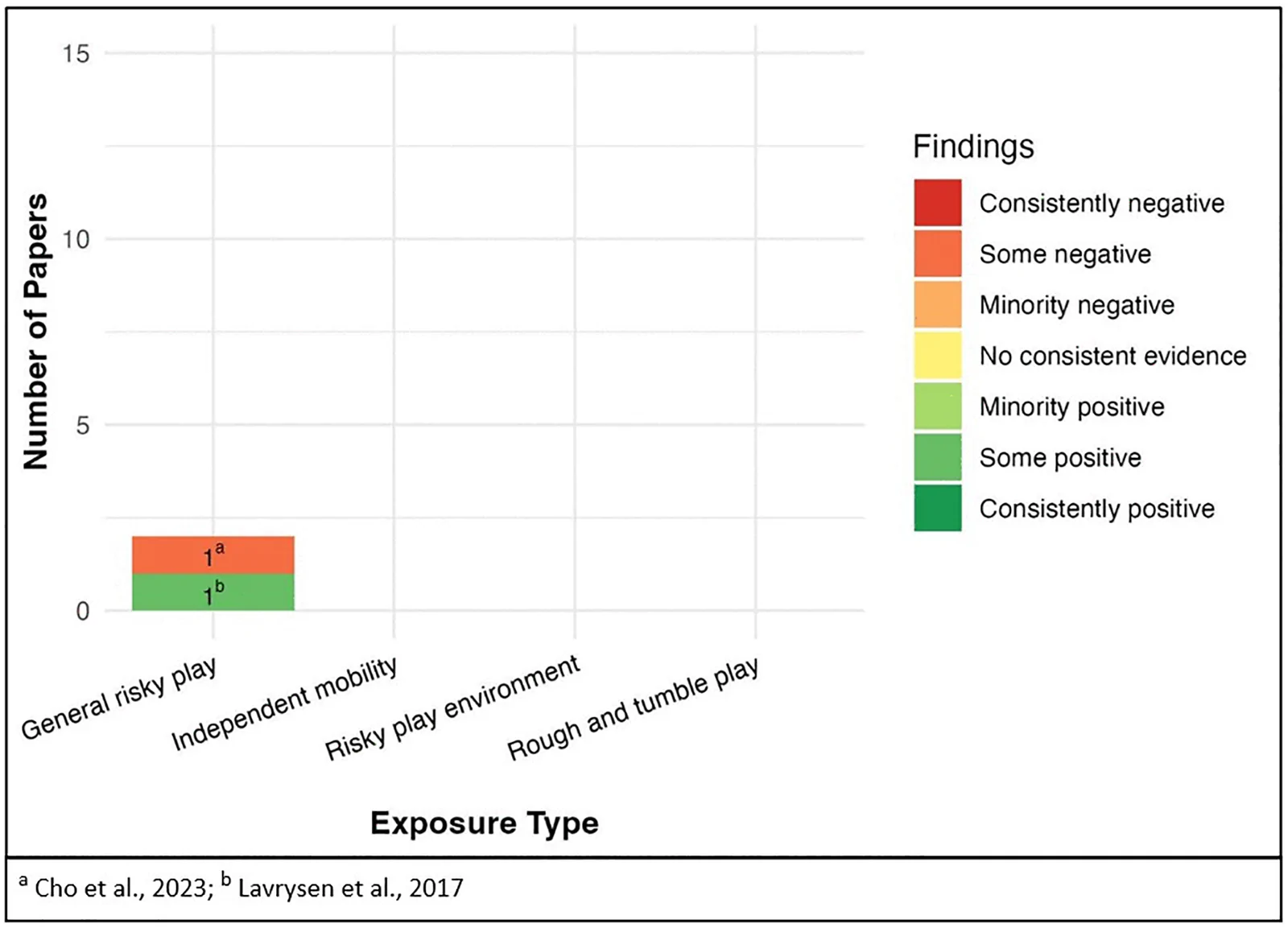
The number of studies reporting quantitative findings between risky play and risk competence and injury outcomes, organised by risky play exposure category.

### Study quality

Of the 74 studies, 18 (24%) were rated high quality, 37 (50%) were rated moderate quality and 19 (26%) were rated low quality. Qualitative study designs (n = 19) were primarily high quality (74%); those not meeting high quality (n = 5) had limited coherence between qualitative data sources, collection, analysis and interpretation. Mixed-methods study designs included in this study (n = 7) were often rated low (n = 2) or moderate quality (n = 5), with none rated high quality, due to the different components of the study not being effectively integrated to answer the outlined research question. Only 4 quantitative studies (8%) were rated as high quality; included quantitative studies frequently faced limitations in generalizability of findings, controlling for confounding variables and adequate statistical analyses employed.

### Physical outcomes

#### Quantitative findings.

Overall, 25 studies quantitatively reported on children’s physical health-related outcomes [23,33,36–38,41–43,48,50,52,60,64,66,67,69,74,75,78,79,81,82,93,100,101] (Fig 2). Of these, 14 studies focused on IM as the risky play exposure [23,37,38,48,60,64,66,67,74,75,79,81,82,100], eight on risky play supportive environments [33,36,41–43,50,52,78] and three on general risky play [69,93,101]. No studies reported on R&T play and physical health outcomes. Most physical outcomes were PA-related outcomes (n = 23), including the number of minutes or counts for different PA intensities (sedentary, light, moderate, moderate-to-vigorous, vigorous) and total PA, such as the number of steps per day and the reported or observed number of minutes in various PA categories and domains [23,33,36–38,42,43,48,50,52,60,64,66,67,74,75,78,79,81,82,93,100,101]. Within studies reporting PA-related outcomes, 18 (78.3%) studies reported positive associations between risky play exposure and PA outcomes [23,37,38,43,48,50,60,64,66,67,74,78,79,81,82,93,100,101], 2 studies (8.7%) reported no association [33,75] and 3 studies (13.0%) reported negative associations [36,42,52]. Other reported physical outcomes included cardiorespiratory fitness [79] and anthropometric-related outcomes [52,64,79], including body mass index (BMI), waist circumference and waist-to-height ratio. Motor competence outcomes included fundamental movement skills, object control skills, locomotor skills and motor control [36,41,69,101].

Physical outcomes were derived using a number of measurement techniques, including accelerometers [23,33,36,37,42,43,50,52,60,74,78,81,100], pedometers [67,75], questionnaires [38,48,64,66,79,82], direct observation [93] and whole body motion capture [101]. There was considerable variability in the methods employed to monitor and report PA behaviour, even when the same type of measurement technique was used. Most physical health outcome studies were cross-sectional (n = 17); 4 were RCT designs and 4 reported other study designs (longitudinal, case-control, non-randomised experimental, convergent mixed-methods).

All studies that examined children’s IM and a physical outcome focused on PA, with most reporting a positive relationship between IM and PA [23,37,38,48,60,64,66,67,74,79,81,82,100]. Among these studies, the relationship between IM and active transport also reported significant positive associations, with children who are granted more IM engaging in greater amounts of active travel [38,67]. There were no significant associations between IM and any fitness or anthropometric variables [79]. Gender-specific associations were observed across included studies investigating IM and PA. Two studies outlined a positive relationship between IM and PA for girls [23,48], while another study found a positive relationship in boys only [82].

Studies investigating the associations of interventions that modified the playground environment (risky play supportive environment category) in schools and child care centres (through the introduction of loose parts, recycled materials etc.) produced mixed results [33,36,41,43,50,52,78]. Among these studies, 3 reported positive results related to children’s physical health [43,50,78], 3 reported negative results [36,42,52], and 2 reported no consistent evidence [33,41]. Playground interventions [41,43] and type of playground environment [36] did not have any influence on anthropometric measures [52] or fundamental movement skills [41,43]. Of the three studies examining children’s general risky play in relationship to physical health outcomes [69,93], two reported positive associations with children’s physical activity [93,101] and a third paper reported no consistent evidence related to children’s motor control [69].

#### Qualitative findings.

Most studies investigating children’s risky play and behaviour in risky play environments using qualitative methods reported vigorous physical activities, such as climbing, jumping, wrestling, carrying, and riding, swinging and running fast [34,41,43,46,51,57,63,73,80,92,94–97,105]. Children participating in loose parts play interventions or play in natural settings, such as forest/bush kindergartens, were physically active in diverse ways, which was perceived to enhance their strength [43,46,73,97,102], motor competence (including balance, coordination, and movement skills) [34,41,46,51,63,73,76,94–97,99,102,103] and cardiovascular fitness [43,44,51,73,92]. A researcher observing Canadian preschool children’s play with loose parts in a rural natural environment, described children “…*enjoying the freedom of outdoor spaces and the openness of the early childhood educators to movement and verbal expression, such as running, chasing, fleeing, climbing, and screaming”* [57]. While a New Zealand Primary School leader observed that following a year long loose parts play intervention, “*We have noticed that our children…we do those preparatory walks before going to Year 8 camp…that they’re much ﬁtter as a general group and we’re only, well, we’re only having to do two walks this year instead of four. The kids are much ﬁtter*” [51]. During risky play, children were observed to challenge themselves and “push themselves out of their comfort zone” [97] supporting new physical skills and “*feeling a sense of achievement*” [99]. A Dutch child attending a loose parts play afterschool program explained, “*It is scary, but then I just do it anyway*” [103] Children’s play in these settings frequently involved complex movements (climbing, jumping, pulling, lifting, pushing), which children repeated in creative ways with small variations, progressively taking on greater physical challenges as their skills developed [41,51,96]. A Canadian educator explained that during loose parts play, children *“…really push themselves physically, and…over time they’re just taking bigger risks, using more muscles you know, using different muscles*” [97]. Children reported that they enjoyed adding physical challenges and competition to their play, for example playing soccer on a trampoline for added excitement, which for one 8-year-old Irish boy was “*like you are exploding your head*” [63].

### Mental health and well-being outcomes

#### Quantitative findings.

Thirteen papers included mental health and well-being outcomes [42,45,49,53,56,58,61,68–71,93,104] (Fig 3), of which, five studies focused on R&T play [56,58,61,71,104], four on general risky play [45,49,69,93], two on risky play supportive environments [42,53], and two studies focused on IM [68,70]. The age range of participants across the studies was 2–12 years; the majority of studies focused on younger children up to 6 years old. Two of the studies did not state the ages of the children. Sample sizes ranged from 34 to 1919, with most having fewer than 130 participants. Six of the studies were cross sectional [45,49,58,61,93,104], four were longitudinal [53,56,68,71], one used a case report design [70], one used a convergent mixed-methods repeated measures design [42], and one was a non-randomised experimental study [69]. A combination of observations and questionnaires, with child, parents and/or teachers as responders were used.

As shown in Fig 3, there was significant variability between studies in relation to the association between risky play and mental health and well-being outcomes. A positive association was clearest for the four studies with general risky play as the exposure. General risky play was found to be associated with well-being [93], fewer internalising problems and more positive affect [58], higher levels of self-control [45], and higher self-esteem [69]. Although these studies provide fairly consistent positive evidence, all of the studies were cross-sectional, and only one study used an intervention/control design [69]. Also, within these studies there were some inconsistencies, such as findings not being replicated using independent observer ratings [69] or associations only being found for lower income families [49].

Results were overall negative in relation to associations found between R&T play and mental health and well-being [56,58,61,71,104]. Two studies focused on parental R&T play [56,58], where the remaining focused on R&T play with their peers, mainly in an education setting [61,71,104]. Within studies examining parental R&T play, paternal factors were found to moderate the negative associations. Paternal anxiety was found to be a moderator in the relationship between R&T play and child anxiety [58], parental gender differences in R&T play participation were observed [58], and lower emotional regulation scores were reported only at higher levels of father dominance [56]. Results from studies focused on R&T play in school or child care settings found that more R&T play increased emotional dysregulation at home [104] and positive emotional regulation skills [61]. However, one study demonstrated R&T play was associated with more negative affect expression and less positive affect expression following an R&T, but not with emotion regulation [71].

Two studies focused on risky play supportive environments, with positive associations reported on children’s mental health and well-being, including overall mental health [42] and happiness at school [53]. Farmer et al. [53] highlight that their school playground intervention significantly improved children's reported happiness only after two years, with the one-year results being non-significant. Only two studies examined IM in relation to children’s mental health and well-being and they had notably different findings. One study found that parental licence to cross roads was negatively associated with happiness, with lower ratings of happiness for those who were allowed to cross the road independently [70]. However, the authors note that this finding was likely due to children’s desire for companionship as children enjoyed walking more than being transported, but independent travellers were less happy on their school journeys than their adult-accompanied counterparts. In this study, no associations were found between independent travel after school and happiness [70]. In the second study examining IM in relation to children’s mental health and well-being, home range, but not mobility licence, was associated with lower distress, with children allowed to travel further away from home having lower distress levels [68].

#### Qualitative findings.

Children’s enjoyment of outdoor risky play was emphasised in over half of the studies, ranging from children’s self-described “*fun*” [34,39,46,63,92,94,103], to caregiver perceptions of *“pure joy”* [99], “*fearful joy*” [95] or enjoyment [41,43,80,97], and researcher observed “*exhilaration*” [94]. Self-confidence was another frequently reported outcome for children; both general confidence or self-belief [34,42,51,57,73,76,80,95,97,99,102,103], and task and skill-specific confidence, which we describe as self-efficacy in the synthesis [41,46,63,94–96,103]. Parents, educators and researchers commonly reported that when children challenged themselves physically and emotionally, the experience of ‘mastery’ over an activity contributed to a sense of accomplishment and self-efficacy. A Norwegian researcher observing preschool children’s outdoor play described: “*Sam (4) is trying to get up in a thin but high tree…Sam has difficulties, but he keeps on trying…He slowly ascends the tree, little by little. After some while he reaches the few branches right under the top of the tree…He now stays up in the tree, approximately 2 metres over the ground…he says, directed to himself: ‘I really did dare that!*’” [94]. Parents and educators perceived that through risky play, children developed the ability to regulate strong emotions by learning how to understand and manage emotional responses such as anxiety, fear, frustration and anger [41,42,57,73,76,99,102]. An Australia mother explained: “*They’re learning how to deal with their own anxieties. So one of the things they learn is how they personally deal with situations they’re uncomfortable with and how they need to manage any fear or anxiety they may have*” [73]. These experiences were also perceived to build resilience [34,43,73,80,99] in children by learning how to deal with physical and emotional setbacks. For example, “*…children who fell over were more likely to pick themselves up and continue playing rather than cry as they might previously have done*” [43].

### Aggression outcomes

#### Quantitative findings.

Thirteen papers included aggression-related outcomes [42,54–56,77,83–85,88–91,104] (Fig 4). One was an intervention study to promote nature-based risky play in child care centres (a risky play supportive environment exposure), which found no association with teacher-reported relational or overt aggression [42]. The other 12 studies focused on R&T play as the risky play exposure. Of these, three studied father-child R&T play specifically [55,56,83] and the rest focused on children’s R&T play with one another, often in an education context. Three studies focused only on boys [54,88,89]. Age ranges of participants varied from age 2 through to adolescence. Most studies had small sample sizes of <100 participants and were cross-sectional studies using correlation or regression to examine associations. Exceptions to this were two studies which included a longitudinal design or element [56,89], and one intervention study with a pre-post comparison [42]. A combination of observation and questionnaires, with child, parents and/or teachers as responders, were used.

Overall, Fig 4 shows a clear trend for R&T play frequency to be associated with more aggression in children, classified as a negative health outcome in this review. Although there was striking consistency in the direction of effects found, there was significant inconsistency within studies, with only one having consistent findings [88]. Several studies examined moderators of the association between R&T play and aggression-related outcomes, which provide indication of what factors may underpin the observed inconsistencies. Two studies found R&T play with fathers was only associated with more aggression when fathers were relatively less dominant playmates [55,56]. Three studies examined whether there were different patterns by gender [54,85,104] with one finding R&T play was only associated with aggression in girls [85], one finding R&T play was associated with less aggression in girls, although the interaction with gender was not significant [104], and one finding inconsistent patterns across genders [54]. Furthermore, two studies examined social status as a moderator; both found a significant association only in children who were socially rejected [54,88], with non-significant trends in the opposite direction for children who had higher sociometric status.

#### Qualitative findings.

Qualitative studies reported mixed findings for aggression-related outcomes in relation to children’s risky play. Some studies described instances of negative behaviours with peers [103] such as “*tattling, bossiness, testing of social limits, and exclusion of others*” [57], while other studies reported *“…behavioural problems on the playground have been reduced*” [44] and less bullying and conflict in risky play supportive environments [43,44,51]. A New Zealand primary school principal participating in a loose parts intervention [51] explained: “*We have less teachers on duty now because the positive outcomes in terms of behaviour, kids being busy, there being less bullying, less conﬂict in the playground, means that we don’t run a time out space anymore for playground, you know, problems*.” Two studies investigating R&T play found children learn how to regulate aggression to keep it fun and exciting, but, both studies found that in some instances playfighting could turn into real fighting [94,99].

### Social outcomes

#### Quantitative findings.

Overall, 16 papers [42,44,47,53,62,65,69,72,77,84–89,91] included quantitative outcomes relating to social outcomes (Fig 5). Most studies (11/16) focused on R&T play as the risky play exposure [47,62,72,77,84–88,91], with the remaining studies focusing on risky play supportive environments [42,44,52,53] and general risky play [65,69]. Three of the studies [87–89] only included boys in the sample. The ages of participants ranged from 2–13 years. One study [65] did not report the age of participants but referred to them as preschoolers throughout. Sample sizes ranged from 31 to 840, however, most studies (11/16) had sample sizes of <100 participants. Most of the studies (10/16) were cross-sectional in design [47,62,65,72,77,84,85,87,88,91], with two adopting longitudinal designs [86,89], one was a non-randomised experimental study [69], one was a repeated measures intervention study [42], and two used a randomised controlled trial design [44,53].

Fig 5 outline the mixed findings for the association between R&T play and outcomes related to social outcomes. Observed R&T play was not found to have any significant associations with outcome expectations [62], social affiliation [86], social cognition [86], observed pro-social behaviours [91] or social problem solving [88] in children of varying ages. Significant positive associations with social problem solving were reported by Pellegrini et al. [84], however, associations were only significant in children who were rated as being popular and not those who were rated as rejected. Associations were also shown to vary by gender, with positive correlations with social competence observed in boys [47,85,87], but not girls [85]. Similar findings were also reported for peer acceptance [47,72]. The gender of the playmate for R&T play also influenced findings; Colwell and Lindsey [47] reported negative associations between R&T play with mixed-sex peers and peer acceptance and social competence, for boys only, while same-sex R&T play was associated with better peer acceptance for boys. Similarly, Pellegrini [89] found that boys engaging in R&T play were less likely to be liked and more likely to be a victim. It is noteworthy that most studies (9/11) investigating the associations between R&T play and social outcomes were published more than 20 years ago [47,62,84–88,91], with the most recent study published in 2014 [72].

Three studies examined risky play supportive environments, with two studies reporting no significant findings related to social outcomes [44,53], and one study finding that peer problems decreased following a risky play environment modification at child care [42]. Studies that looked at the influence of general risky play on social outcomes demonstrated positive associations [65,69].

#### Qualitative findings.

Commonly reported social outcomes of risky play included improvements in social competence and interpersonal skills needed for building friendships [42–44,57,63,92,98,99,103,105], and cooperation skills for playing and getting along with other children [34,41,43,46,51,57,63,98]. Educators, teachers and researchers observed that risky play supportive environments, like loose parts play, provided rich opportunities for children to practice social skills [34,41,43,46,51,57,63,98]. A Canadian ECEC educator explained: “*So, it was really nice to see how they worked together and were mentoring each other and cooperating and helping the younger ones*” [41]. While an Australian primary school study [43] found “*…most teachers reported gains in social play. For example, children who did not usually play together (e.g., children in different age groups) were more likely to do so given the availability of the* [loose parts play] *materials*.” Children’s communication skills [41,43,57,103] and skills for resolving problems with other children [40,57,103] were also perceived to improve through risky play. “*They* [children in a Dutch afterschool loose parts program] *also shared some undesirable experiences, like when one child spoils play for another child. Children usually view these risks positively, as a natural part of playing together…They must indicate their own borders, and making agreements is necessary when rough-and-tumble play is going on*” [103]. Children described how friends could make risky play more thrilling and exciting, and opportunities for IM, where children were allowed to explore their neighbourhoods autonomously, enabled them to create meaningful social experiences with friends [92,105]. A Swedish researcher explained: “*They* [children] *also pointed out that being independent made it easier to make after-school plans with friends.* [As described by] *Tilde: ‘And it’s easier if you want to hang out with a friend or someone else after school if you cycle or walk.’”* [92]

### Cognitive outcomes

#### Quantitative findings.

Only five papers included outcomes that were categorised as cognitive outcomes [54,59,61,69,77] (Fig 6). Four of these focused on R&T play [54,59,61,77] and one on general risky play [69]; participating children were aged between 4 and 7 years (one study did not provide participant ages [61]). Four studies were cross-sectional [54,59,61,77] and one used a non-randomised experimental design [69]. Sample sizes ranged from 76 to 357 and the age of children ranged from 3 to 8 years of age. A combination of parent and teacher report were used to assess risky play, with one study also using observations [77] and one study evaluating an intervention [69]. All studies assessed outcomes via teacher and parent report, with one study also using a standardised working memory assessment [59].

Fig 6 shows mixed associations between R&T play and cognitive and academic outcomes. Of the four R&T play papers, two were focused on the school environment [61,77], one on R&T play with fathers [59] and one on children’s desire to engage in R&T play [54]. Only one R&T play paper found positive associations, focusing on father-child R&T play in young children (assessed via questionnaire) and working memory skills [59]. Negative associations were found in two R&T play papers related to working memory and inhibitory control [61] and inattention and hyperactivity [54]. One study discovered no significant associations between R&T play and distractibility [77]. Only one paper examined general risky play in the context of cognitive outcomes and found that a school-based risky-play intervention improved concentration relative to controls [69].

#### Qualitative findings.

Most studies investigating children’s play in risky play supportive environments (i.e., loose parts play [41–44,51,57,76,98,103]; nature play [34,63,102]) observed children engaging in imaginative, dynamic and creative play. Educators observed that children developed inventive ways to play in these environments, such as building structures and creating imaginative role-playing scenarios. Canadian nature preschool children describing their play [34] explained: “*I like the trail in back woods: I play running to ﬁnd a baby cheetah”, “I like to run in the woods and play Ninja Turtles*.” While an older Dutch child attending a loose parts play afterschool program described “*We always play astronaut; the parasol is then the satellite and the barrel is the rocket which rolls down the mountain. I then go after the barrel with the buggy or run with the parasol after it to have enough reach*” [103]. Studies also found that children developed a sense of awareness of their bodies in movement through risky play [34,41,46,99], learning how to respond to unpredictability in the environment; and practice problem solving [40–42,57,73,80,97]; which in one study involved a preschool child navigating an obstacle course backwards without colliding with other children [41]. A Canadian mother described this learning experience for her daughter as: “*Allowing her to figure out what her strengths and weaknesses are…like natural consequences and help like allowing her to problem solve by herself without me having to tell her or guide her or direct her*.” [40] Additionally, several studies reported young children’s language development [41,44,57] and experiential learning, for example, in sciences [34,96,102], were fostered in risky play supportive environments. In an Australian Bush Kinder program, “*Bernie [educator] asked ‘why do you keep falling down, what makes things fall down from trees?’ Poppy [child] replied, ‘I think it is gravity.’ Bernie, continued to ask Poppy about gravity then asked Poppy about what she had learned about climbing. Poppy said she had to hold on tightly*” [96].

### Risk competence and injury outcomes

#### Quantitative findings.

Two papers included outcomes related to risk competence [69] or injury outcomes [45] (Fig 7). Both these studies [45,69] focused on young children (3–5 years and 3–8 years, respectively) with sample sizes ranging from 76 [69] to 862 [45] participants. Cho et al. [45] used a cross-sectional design and a questionnaire to establish exposure to risky play and assess injury. This study found that children who tried more diverse types of risky play experienced more injuries than those who tried fewer types of risky play [45]. However, this study found that child self-control moderated the association between risky play exposure and injury outcome, with little effect of exposure on injury in children who had higher self-control [45]. Lavrysen et al. [69] examined the effects of a risky play intervention through a non-randomised experimental study design, finding a positive association between risky play and risk competence on a computer-based risk detection assessment, but no association when considering observed risk competence in the playground [69].

#### Qualitative findings.

Children’s development of skills to manage risk in play outdoors was a frequently reported outcome across qualitative studies. We have categorised these outcomes using dynamic risk management theory, which describes three interrelated components of children’s risk competence: risk willingness, risk assessment and risk handling [106]. Children’s risk willingness, their openness or inclination (strong or weak) to take risks, was described in eight studies [39,46,57,63,94,97,103,105]. Openness to risky play was linked to children’s emotional responses and perceived skills for managing risks, which could change over time. As a Canadian child reported: “*Risky play is fun … It’s just a feeling of anticipation*” [39]. Studies emphasised the subjective experience of risky play – what was risky for one child may not be risky for another. As a researcher observing Irish children playing outdoors explained: “*Aoife (aged 8) stepped on the lowest branch. She described this as ‘scary’ and ‘exciting’. Dan (aged 7) climbed eﬀortlessly to the top of the tree, remarking, ‘it’s so easy’.*” [63]

Children’s ability to assess risk was described in 13 studies in terms of the development of skills to evaluate a risky activity and decide whether or not they should do it [35,39–41,46,57,73,76,80,94,97,98,102]. As a Canadian forest kindergarten teacher describing a student’s risk assessment skills observed: “…*She knows her physical limitations. But [she] will try things if she sees the other children trying them and if she thinks that they’re safe.*” [46]. While, a Norwegian researcher described “*the child first approached the play [climbing, jumping and balancing] but decided to back off after a moment of consideration, as if the child wanted to partake but did not dare to*.” [94] Natural environments and loose parts play environments were particularly conducive to children learning how to assess risks and determine their own risk-level, as a Canadian educator reported: “*it [loose parts] gives them the opportunity to scale their risk, depending on what they’re comfortable with”.* [97] While a Canadian forest kindergarten teacher explained: *“…If someone [child] wants to climb a tree, which they’re most welcome to do, they make sure that one of the grown-ups is nearby [and] able to watch…they also then check all of the ground around for anything they may hurt themselves on…And then if there are branches low enough down, they try to wiggle them and see if they’re going to be strong enough for their weight”* [46]*.*

Children’s handling of risk was described across 14 studies, particularly the ways children improved their skills for risky play, such as practicing physical skills, observing how their peers managed risks, or gradually pushing their own limits with a caregiver nearby [35,39–41,43,46,51,57,63,73,80,96,97,102]. A Canadian researcher observing a loose parts play intervention in preschool reported: “*The children rarely rushed into a risky situation without testing or questioning the action or environment. Oftentimes they would take sequential steps as part of completing an entire risky movement*.” [57] A principal of a New Zealand primary school participating in a loose parts intervention described children’s skill development for managing risks in the playground: “*It was interesting that you know one or two people again were a little bit, ‘Ooh’ you know ‘cause one child got wiped out by a tyre but then do you ban the whole thing because someone got hit by a tyre? No, everyone just after that knew to look…watch out for the tyres*.” [51] Similarly, a teacher in a US nature preschool explained: “*We use real adult tools, with supervision, but with that does pose its own risks…The kids are very interested in using them, so they’re also careful because they know if they’re not using them properly, they don’t get to use them*.” [102]

Across 12 studies, parents and caregivers described the role of occasional injuries as a positive outcome in children’s learning, where children build new skills, become stronger and better able to assess and manage risks in the future, when they are challenged, have the opportunity to take risks, make mistakes or fail, and sometimes experience minor injuries during outdoor play [34,40,41,43,73,76,80,95,97,99,103,105]. We have drawn on the concept of ‘antifragility’ to describe this outcome [107]. A Norwegian preschool educator explained: “*They could get hurt, but still they HAVE to be allowed to take risks − they have to find out that it wasn’t such a big deal if I fell down−If they never experience this...then suddenly they’re at a point in life where they REALLY fall, then it becomes serious...they have some scrubs and bruises − that’s a part of life.*” [95] Similarly, a Canadian caregiver said “*It’s [a] time for [kids] to try to learn and basically know that if they make a mistake or if something happens that they can get back up, they can try a different way or not do that again*” [76]. One Australian mother emphasised “*Risk-taking is all about that we have to let children use trial and error to learn from their mistakes.*” [73]

There were mixed findings across and within studies regarding perceptions of injuries, how likely they were to occur or how serious they might be during risky play, or in the case of play interventions where injury incidence was observed or recorded, how often they did occur [35,39,40,42–44,73,76,80,95,97,99,102]. Studies also discussed injuries in varying ways making comparison between studies difficult. Some compared risky play or risky play environments to other types of outdoor play and reported no change in injuries [43,44,97], or fewer or similar ‘minor’ injuries, like scrapes, bumps, bruises and small cuts [35,42,102]. For example, a teacher from a US nature preschool explained, “*I guess there’s probably more scrapes and cuts and bruises [at a nature preschool]. Then of course we’re walking on rocks and…uneven surfaces”* [102]. While an Australian loose parts play primary school intervention reported: “*Although, some teachers worried about safety, only 1 incident required medical attention, no greater incidence than usual over the time period*” [44]. Studies investigating parent, educator or child perspectives on risky play, made a distinction between minor injuries, described as scrapes, small cuts, grazes, bruises, which were perceived positively, as an expected part of risky play when children are learning about their bodies and abilities and building new skills, and more serious injuries, commonly described as those needing emergency care, which could occur in dangerous play situations [39,40,73,76,95,99]. As a Canadian child explained: “*Danger is like … jumping off a very high place without being a professional … then risk is like, you have a chance of getting hurt, but you also have a chance of surviving or living or not getting hurt … Risk can be legs scraped, banged on the woodchips*.” [39] While a director at a US nature preschool reported: “*It’s mostly superficial, scrapes and bumps and bruises, and occasionally bee stings. We haven’t had any broken bones or sprains, or anything really intense.*” [102] No qualitative studies reported occurrence of serious injuries.

### Synthesis of quantitative and qualitative findings

The synthesis of quantitative and qualitative results is summarised in Table 3, with the overall direction of evidence for each health domain and risky play exposure depicted in Fig 8. The complete mixed-methods synthesis results are reported in S4 Appendix. General risky play and risky play supportive environments were positively associated with physical, mental health and well-being, social, cognitive, and risk competence. General risky play and risky play supportive environments were also associated with positive health outcomes for injury domains. Evidence for R&T play was inconsistent across all health outcome domains, except for a negative association with aggression (i.e., increased aggression). IM was understudied except in relation to physical and social health outcomes, where positive associations were derived from the synthesis. Quality of evidence varied across exposure-outcome pairings; qualitative studies were more consistently rated moderate-to-high-quality across health domains, whereas quantitative study quality was more variable.

**Table 3:** Synthesised findings and quality of evidence on the association between risky play and health outcomes, by outcome category and exposure type.

Health Outcome Category	Exposure Type	Quantitative Evidence & Relationship^a^	Qualitative Evidence & Relationship^a^	Quality of Evidence (overall) (%)			Synthesised evidence (Pillar synthesis)
				Low	Moderate	High	
Physical	General risky play	+ [43,50,78,101] * [33,41,75] - [36,42,52]	+ [63,73,80,94,95]	20.0	46.7	33.3	Moderate Positive
	Risky play supportive environments	+ [93] * [69]	+ [34,41,43,44,46,51,57,76,96,97,102,103]	28.6	38.6	42.9	Moderate Positive
	Rough-and-tumble	n/a	+ [99]	n/a	100.0	n/a	Insufficient
	Independent mobility	+ [23,37,38,48,60,64,66,67,74,79,81,82,100] ^*^ [75]	+ [92,105]	12.5	62.5	25.0	High Positive
Mental Health and Well-being	General risky play	+ [45,49,69,93]	+ [39,63,73,80,94,95]	10.0	20.0	70.0	High Positive
	Risky play supportive environments	+ [42,53]	+ [34,41–43,46,51,57,76,96,97,102,103]	28.6	35.7	35.7	High Positive
	Rough-and-tumble	+ [58,61] - [56,71,104]	+ [99]	16.7	83.3	n/a	Insufficient
	Independent mobility	+ [68] - [70]	+ [92]	n/a	66.7	33.3	Insufficient
Aggression	General risky play	n/a	* [94]	n/a	n/a	100.0	Insufficient
	Risky play supportive environments	+ [42]	+ [43,44,51] - [57,103]	16.7	50.0	33.3	Insufficient
	Rough-and-tumble	- [54–56,77,83–85,88–91,104]	* [99]	69.2	30.8	n/a	Low Negative
Social	General risky play	+ [65,69]	+ [40,63]	50.0	n/a	50.0	High Positive
	Risky play supportive environments	+ [42,52] * [44]	+ [34,41–44,46,51,57,98,103]	15.4	53.8	30.8	Moderate Positive
	Rough-and-tumble	+ [72,77,84,85] * [62,86–88,91] - [47,89]	+ [99]	33.3	66.7	n/a	Insufficient
	Independent mobility	n/a	+ [92,105]	n/a	n/a	100.0	Low Positive
Cognitive	General risky play	+ [69]	+ [40,63,73,80]	20.0	n/a	80.0	Low Positive
	Risky play supportive environments	n/a	+ [34,41–44,46,51,57,76,96–98,102,103]	21.4	42.9	35.7	Moderate Positive
	Rough-and-tumble	+ [59] * [77] - [54,61]	+ [99]	40.0	60.0	n/a	Insufficient
Risk Competence	General risky play	+ [69]	+ [39,40,63,73,80,94,95]	12.5	12.5	75.0	Moderate Positive
	Risky play supportive environments	n/a	+ [34,35,41,43,46,51,57,76,96–98,102,103]	23.1	30.8	46.2	Moderate Positive
	Rough-and-tumble	n/a	+ [99]	n/a	100.0	n/a	Insufficient
	Independent mobility	n/a	+ [105]	n/a	n/a	100.0	Insufficient
Injury	General risky play	- [45]	+ [39,40,73,95] - [80]	n/a	33.3	66.7	Low Positive
	Risky play supportive environments	n/a	+ [35,42,44,97] * [43,102] - [76]	28.6	42.9	28.6	Low Positive
	Rough-and-tumble	n/a	+ [99]	n/a	100.0	n/a	Insufficient

+ = positive relationship; - = negative relationship; *= neutral/no directionality relationship

^a^Relationship direction is related to a positive or negative health outcome (i.e., for health outcomes where a decrease in occurrence was reported as beneficial to children’s health, such as aggression, a positive relationship indicates a decrease in these outcomes).

n/a = no studies examining this relationship included in this review.

**Fig 8:**
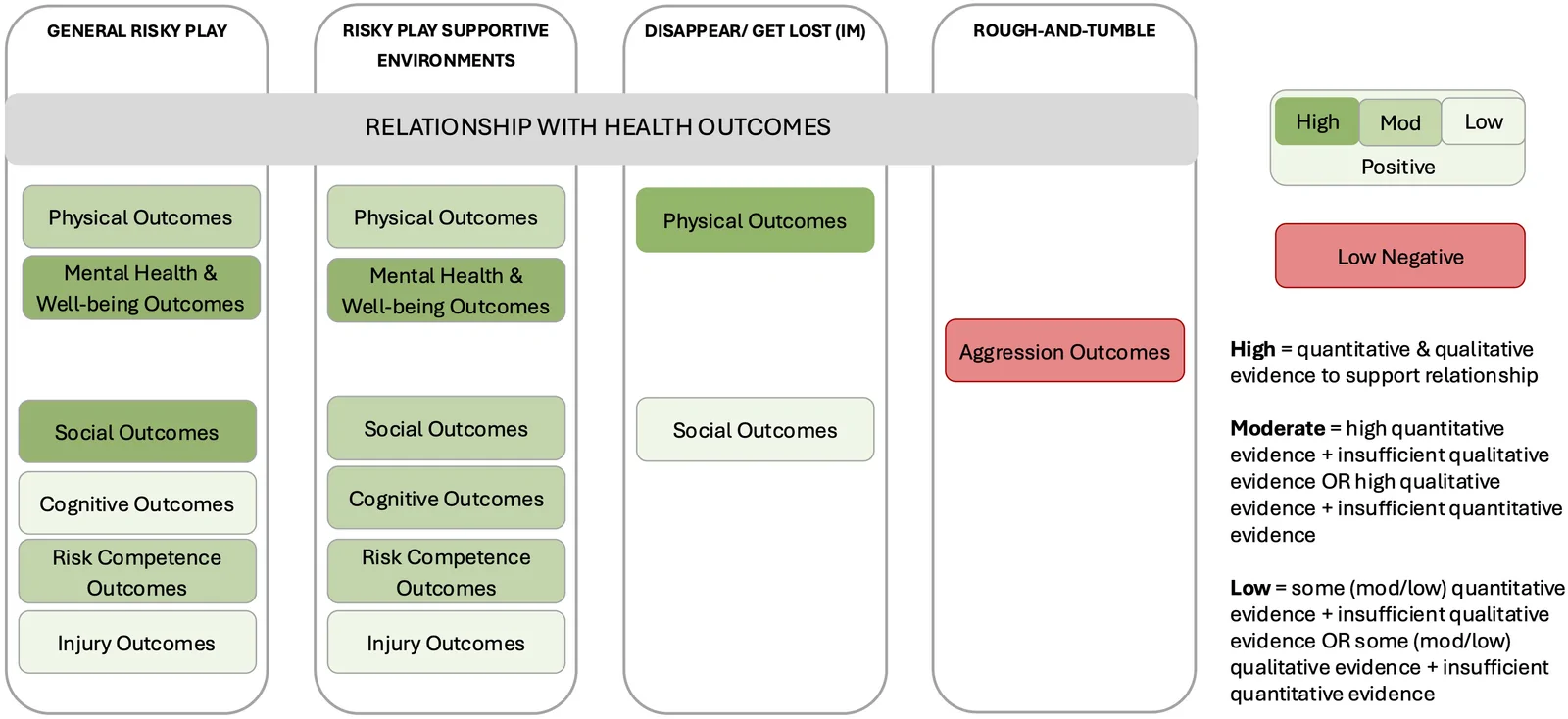
Synthesised results of the literature examining quantitative and qualitative evidence on the relationship between risky play (exposure) and health outcomes (physical, mental health & well-being, aggression, social, cognitive, risk competence and injury).

Physical health outcomes were positively associated with all risky play exposures except R&T play, which was investigated in only one qualitative paper. For IM, physical activity was the only outcome studied, yielding a positive association in the synthesis supported by predominantly moderate-to-high-quality evidence (87.5%). For general risky play and risky play supportive environments, inconsistent quantitative findings on physical activity and perceived motor competence were offset by qualitative evidence outlining positive relationships with a range of outcomes (e.g., vigorous physical activity, motor competence, fitness, strength).

Mental health and well-being outcomes, including emotional self-regulation, enjoyment, well-being, and self-confidence, were positively associated with both general risky play (90% moderate-to-high-quality evidence) and risky play supportive environments (71% moderate-to-high-quality evidence). R&T play and IM showed discrepant findings and limited evidence for this domain, yielding insufficient evidence. Aggression outcomes were largely understudied, except for R&T play, where positive statistical associations with aggression (i.e., increased R&T play was associated with increased aggression) were found in the quantitative literature (n = 12), which was predominantly low quality (69.2%) and conducted between 1988 and 2003. With only a single, moderate-quality qualitative study available, the overall synthesis classified this relationship as an adverse (negative) health outcome finding between risky play and aggression.

Social outcomes, including social competence and communications skills, were positively associated with general risky play (evidence evenly split between low and high quality) and risky play supportive environments (predominantly moderate-quality evidence). IM was not quantitatively studied for social outcomes, but a small, high-quality qualitative evidence base (n = 2) supported a positive relationship. R&T play evidence was mixed and limited, yielding an insufficient synthesis. Cognitive outcomes were mixed and understudied in the quantitative literature across all exposures. General risky play and risky play supportive environments were both positively associated with cognitive outcomes, driven almost entirely by moderate-to-high-quality qualitative evidence (100% and 79%, respectively). The evidence base for R&T play and IM was too limited across both pillars to draw conclusions.

Risk competence and injury outcomes were similarly understudied quantitatively. Risk competence was positively associated with both general risky play and risky play supportive environments, supported by mostly moderate-to-high-quality qualitative evidence (100% and 77%, respectively). Evidence for injury outcomes was mixed; however, a positive association was synthesised for general risky play and risky play supportive environments related to injury outcomes. This finding was primarily supported by moderate-to-high-quality qualitative evidence outlining fewer or only minor injuries in relation to general risky play and risky play supportive environments. IM and R&T play were understudied for both risk competence and injury outcomes, yielding insufficient synthesised evidence for these health domains.

## Discussion

This systematic review synthesises current evidence on the relationship between outdoor risky play and health outcomes in children aged 3–12 years. There has been a three-fold increase in research in this area since Brussoni et al. [13] conducted the first systematic review on children’s health outcomes related to risky play in 2015. Despite this, evidence quality remains a limitation; of the 74 included studies, only 24% were rated high quality, with qualitative studies rated high quality far more often (74%) than quantitative studies (14%). The results of this review highlight qualitative evidence as generally more consistent and higher quality, while quantitative evidence is more uneven and, in some domains, actively limits confidence in the observed associations.

### Consistent findings across the evidence base

The relationship between children's independent mobility (IM) and physical activity (PA) was the most consistently supported finding in this review, drawn from a comparatively strong and higher-quality evidence base [23,48,60,64,67,74,81,82] and consistent with prior literature [108]. This more robust evidence base for IM may be because both IM and PA are more easily and consistently measured, reducing the measurement heterogeneity that likely contributes to inconsistency elsewhere in this review. This review also highlights a need to extend inquiry beyond PA to other physical outcomes for IM, including motor control, muscular strength, and functional movement skills, where the evidence base remains thin.

Risk competence outcomes showed a similarly consistent positive relationship across risky play exposures, though this rests almost entirely on qualitative evidence [40,51,63,73,94–97,103]. Quantitative risk-competence measures may be less developed or standardized than the observational and interview-based methods qualitative researchers have used to characterize this construct. Until novel and validated quantitative measures of risk competence are established, this evidence base is likely to remain qualitative led.

Outdoor risky play was positively associated with children’s mental health and well-being, including self-confidence, self-efficacy, resilience, and self-regulation. This relationship was supported by qualitative evidence across all risky play exposures, whereas quantitative findings were more mixed for R&T play and IM exposures. The divergence in quantitative findings may reflect measurement philosophy rather than a true difference in effect; quantitative studies typically operationalized mental health through deficit-based tools (emotion dysregulation, socioemotional difficulties), while qualitative studies captured constructs like enjoyment and self-efficacy directly from children's own accounts.

### Divergent findings across the evidence base

The role of R&T play in child development is contested, with perceptions of benefits of play fighting often conflicting across academic and societal realms [109]. The negative association between R&T play and aggression synthesised within this review is a consistent finding, but it rests on lower-quality quantitative literature conducted before 2011 [54–56,77,83–85,88–91]. More recent research [110,111] suggests this finding may partly reflect a measurement artifact of older methods, because distinguishing R&T play from genuine aggression requires attending to reciprocal cues (e.g., consent signalled through body language and vocalization) that earlier observational protocols were not designed to capture [109]. Systematic, consent-sensitive observation protocols carried out by trained researchers can make this distinction, helping avoid R&T play and aggression being conflated.

The relationships between PA and both general risky play and risky play supportive environments were less well-defined in this review, likely reflecting measurement limitations rather than an absence of association. Accelerometers are not able to adequately capture activities like climbing, lifting, or carrying [52], and the available RCT evidence examining this relationship was of uneven quality [44,50,52]. It is also plausible that risky play environments demand a trade-off in how children move; these environments are complex [112] and children may slow down to navigate risk carefully [94], potentially reducing moderate-to-vigorous physical activity while still contributing to other physical skills (strength, balance, spatial orientation, motor competence) that qualitative studies were more likely to detect. Furthermore, the increased engagement afforded by risky play environments may reduce the frequency with which children transition between activities out of boredom, instead encouraging sustained exploration within a single setting [113].

Social outcomes (e.g., social competence and communication skills) showed a positive relationship with most risky play exposures, but the quantitative evidence supporting this was limited or low quality, and most studies drew on parent or educator reports rather than children's perspectives. This is a meaningful gap as children's social experiences during risky play (e.g., negotiating conflict, reading peers’ cues) are best understood from the child's vantage point [114], and future qualitative work incorporating children's voices could substantially strengthen this evidence base.

### Gaps in the current evidence base

Cognitive outcome findings emphasized that risky play environments afforded more imaginative, dynamic, and creative play, awareness of body in movement, and increased engagement in problem solving, language development, and scientific learning. This synthesised finding was primarily derived from moderate-to-high-quality qualitative evidence, underscoring a need for more quantitative evidence examining the association between risky play and cognitive health outcomes. Future research would benefit from a more targeted, theory-driven approach, such as dynamic risk management theory [106], and testing specific cognitive skills mechanistically linked to risky play (problem-solving, creative thinking, executive function under uncertainty) rather than skills with a less obvious connection (e.g., memory, mathematics).

Injury outcomes were mixed, with evidence in one study that injuries increased alongside more diverse risky play, potentially moderated by self-control [45]. Risk competence outcomes, while consistently positive across all included studies, lacked meaningful quantitative corroboration. One plausible pathway linking these two health domains is that minor injuries during risky play exploration may contribute to risk competence, which may in turn reduce the likelihood of more serious injury later [106]; Cho et al. [45] provide some support for this, finding that children with more nature-play exposure showed greater self-control, and that self-control was associated with fewer injuries. As the full pathway from risky play exposure to risk competence to reduced serious injury has not been directly tested, combining objective injury surveillance with validated risk-competence measures is a priority for future longitudinal research. Although injury likelihood, incidence and severity were reported in 13 qualitative studies, injury was only investigated in one quantitative paper in this review. This is a key gap in the literature, given societal concerns about child safety and injury prevention, which have been reported as barriers to children’s outdoor risky play [12,115]. The role of minor injuries, such as scrapes and bruises, in children’s outdoor play, and the impact on child development of preventing all play-related injuries, warrants greater attention.

### Limitations of eligible studies

Qualitative studies consistently reported positive health outcomes across domains and may have been more vulnerable to subjective reporting bias. While quantitative measures have the potential to establish more objective evidence examining the association between risky play and health outcomes, the mixed results observed across quantitative studies suggest that such evidence is not straightforward to obtain. Children’s play is dynamic, fleeting, complex and ambiguous and we may lack appropriate and sensitive measures for assessing these capacities and phenomena quantitatively in children (e.g., interpersonal skills, mental and emotional well-being, creativity) [116,117]. Further, existing measures of risky play vary considerably in their conceptualisation and operationalisation, and many may not fully capture the breadth and complexity of risky play constructs as defined in this review, particularly the experiential, emotional, and contextual dimensions that qualitative findings suggest are central to understanding its relationship with child health.

The quantitative evidence base included a notable proportion of underpowered studies, which are less likely to detect associations and more likely to produce unstable estimates or contribute null findings. Therefore, a lack of significant findings for some health outcomes may indicate that the sample and measurement approaches were not robust enough to identify a substantial finding. Although the methods commonly used in this area may be effective in evaluating behaviour change or PA interventions, their application to play may not yield consistent effects because of a mismatch in measures and constructs.

In addition, the evidence base is predominantly cross-sectional in design, which limits the ability to establish temporal relationships between risky play exposures and health outcomes, and precludes causal inference. The included studies were geographically concentrated in a small number of high-income countries, particularly Australia, Canada, the United Kingdom, and Scandinavian nations, which may limit the generalisability of findings to other cultural, socioeconomic, and environmental contexts.

### Limitations of this systematic review

Several limitations should be considered when interpreting the findings of this review. First, substantial heterogeneity in study designs, exposure operationalisations, outcome measures, populations, and contexts precluded meta-analysis. As a result, findings were synthesised using an adjusted vote-counting approach, which does not account for effect sizes, precision, or sample size, and treats statistically and clinically significant findings equivalently. Nonetheless, this approach is appropriate for heterogeneous evidence bases and, in the absence of sufficient homogeneity to conduct meta-analysis, vote-counting represented the most appropriate available method for synthesising the heterogeneous quantitative evidence base. Additionally, without the ability to report pooled effect estimates, the conclusions regarding direction and strength of associations reported in this study should be interpreted as associative and hypothesis-generating rather than causal. Future reviews with a larger and more homogeneous evidence base may be able to employ meta-analytic techniques to provide more precise estimates of associations and effect.

Searching only five databases, while not exhaustive, was considered sufficient to capture the interdisciplinary literature relevant to this review's scope, though it is possible that relevant studies indexed solely in other databases were missed. In addition, included studies were restricted to those with accessible full texts, introducing the possibility of full-text availability bias, whereby relevant studies published in less accessible journals or databases may have been systematically excluded. While supplementary search strategies were employed to improve coverage of the literature, these approaches may have introduced selection bias if the network consulted reflects particular geographic, disciplinary, or methodological concentrations. Collectively, these limitations reduce confidence in drawing firm, generalizable conclusions from the current evidence base and highlight the need for methodologically rigorous, longitudinal research across diverse populations and contexts.

### Directions for future research

The scope of the literature and mixed findings identified in this review highlight several important directions for future research.

1. A broader range of validated quantitative outcome measures is needed, particularly for constructs currently captured only in qualitative studies, such as enjoyment, self-efficacy, strength, and social development. In addition, the focus on accelerometer data and PA may miss key physical benefits arising from climbing, jumping, and balancing; approaches such as observational tools capturing whole-body movement may be better suited to capturing children's lived experience than accelerometry alone. Future quantitative work would benefit from incorporating strength-based, child-reported measures alongside deficit-based ones, while recognising the challenges of using self-report measures for these constructs [118,119].
2. Aggression-related outcomes require renewed, contemporary quantitative research using consent-sensitive observation protocols, examining R&T play's relationship with a broader range of mental health outcomes, such as emotional regulation, within today's cultural and play context.
3. Future research should develop innovative approaches to assess cognitive outcomes, risk competence, and injury prevention, while remaining attentive to researcher bias, particularly in qualitative work. For example, emerging research has used virtual reality tools to assess the impact of risky play on risk management skills [120,121]. Risky play research should also include measures of injury incidence and severity to address the gap in existing evidence.
4. More robust study designs are required to strengthen the evidence base. Many of the included quantitative studies were cross-sectional, lacked comparison groups, or did not track outcomes over time, limiting the ability to assess the sustainability or directionality of associations. Longitudinal and experimental designs are needed to establish causal links and explore dose-response relationships between risky play exposures and health outcomes. Most studies in this review examined children from high-income countries; further research is needed with populations who may not have the same access to risky outdoor play, including children living with disability, children from low- and middle-income countries, and children from culturally and linguistically diverse or First Nations and Indigenous backgrounds**.**

## Conclusions

This review highlights the unique role of risky play in children’s lives and potential pathways to positive health and development outcomes. Findings indicate that risky play is associated with positive outcomes across physical, mental, social, and cognitive domains, although evidence remains mixed, especially in relation to R&T and aggression. The review also highlights significant gaps in the literature and methodological shortcomings. Qualitative research has been particularly valuable in broadening our understanding of the diverse health outcomes linked to risky play, pointing to directions that future quantitative work should pursue. This review highlights the need for higher quality research that captures the complex and multifaceted ways in which risky play contributes to children’s health and well-being across the lifespan.

## Supporting information

S1 Appendix Search strategy. (PDF)

S2 Appendix Overview of included studies. (DOCX)

S3 Appendix Qualitative evidence synthesis by health outcome. (DOCX)

S4 Appendix Synthesised quantitative and qualitative results. (DOCX)
