# Supplementary material for: Outdoor risky play and health-related outcomes in children aged 3–12 years: A mixed-methods systematic review
Source: PLoS One. 2026 Oct 26;21(10):e0347444. doi: 10.1371/journal.pone.0347444 (PMC13704885; doi:10.1371/journal.pone.0347444)
Supplement: Supplementary file 1 [file pone.0347444.s001.pdf]

S1 Appendix: Search Strategy

For risky play the search included the term ‘play’ paired within three words with one of the following terms: ‘heights’; ‘speed’; ‘tools’; ‘dangerous element’; ‘fire’; ‘rough and tumble’; ‘rough hous*’; ‘horse play’; ‘wrestling’; ‘aggressi*’; ‘fight’; ‘disappear’; ‘get lost’; ‘risky’; ‘adventur*’; ‘independent’; ‘supervision’; ‘unsupervised’; ‘unaccompanied’; ‘unescorted’; adult* presen*; ‘autonomous’. In addition, two secondary sets of search terms were created, one to capture play phrases and one to capture risk phrases. To be included in the screening, papers were required to have both a play phrase and a risk phrase in their title, abstract or keywords. Play phrases were: ‘play’ (as a keyword only); ‘play and playthings’; ‘playgrounds’; ‘childhood play behaviour’; ‘Children’s ‘recreational games’; ‘Children’s play development’; ‘playspace’; ‘play space’. Risk phrases were: ‘risk-taking’; ‘risk-management’; ‘risk-perception’; ‘safety’; ‘safety-management’; ‘adventur*’.

Papers with search terms from **A** in title, abstract or keywords are eligible for inclusion.

Papers with search terms from both **B AND C** in title, abstract or keywords are eligible for inclusion.

A. Risky play/independent mobility terms (combined with OR)
Play N3 Heights
Play N3 Speed
Play N3 Tools
Play N3 “Dangerous element*”
Play N3 fire
Play N3 “rough-and-tumble”
Play N3 “rough and tumble”
(roughhous* or rough hous*)
(horseplay or “horse play”)
Play N3 “wrestling”
Play N3 “aggress*”
Play fight*
play N3 disappear
play N3 “get lost”
risky N3 play
adventur* N3 play
play N3 (unsupervised OR supervision OR without N2 supervision OR unaccompanied OR unescorted OR adult* N2 presen* OR independent OR autonomous)
“independent mobility” AND child*

B. Play terms (combined with OR)
play (*as keyword only*)
“play and playthings”
Playgrounds
Childhood play behaviour
Children’s recreational games
Children’s play development
Playspace
“play space”

C. Risk terms (combined with OR)
risk-taking
“risk raking”
(risk not (“at risk” or risk factor*))
risk-management
risk-perception
safety
safety-management
adventur*

<u>Additional notes:</u>

- The ‘Apply Equivalent Subjects’ expander was used for all searches conducted within EBSCOHost.
- Searches were restricted to journal articles and reports where this is provided as an option within CINAHL only, the search was restricted by age category to only include children because this option was not clearly.

Table S1-1: Characteristics of risky play environments used to guide the systematic review.

**Risky Play Environments**	**Affordances for Risky Play**	**Risky Play Category**
Climbable features	Affords climbing	Great heights
Jump down-off-able features	Affords jumping down	Great heights
Balance-on-able features	Affords balancing	Great heights
Flat, relatively smooth surfaces	Affords running, rough-and-tumble	High speed, rough-and-tumble
Slopes and slides	Affords sliding, running	High speed
Swing-on-able features	Affords swinging	High speed, great heights
Graspable/detached objects	Affords throwing, striking, and fencing	rough-and-tumble
Dangerous tools	Affords whittling, sawing, axing and tying	Dangerous tools
Dangerous elements close to where the children play (e.g. lake/pond/sea, cliffs, fire pits, etc.)	Affords falling into or from something	Dangerous elements
Enclosure/restrictions (e.g. differently sized sub-spaces or private spaces where children can explore on their own or hide away from larger groups, mobility license)	Affords getting lost, disappearing	Disappear/get lost

EBSCOhost CINAHL

Thu, June 25, 2026 10:38

#	Query	Limiters/Expanders	Last Run Via
S8	(S1 OR S2) OR (S5 AND S6)	Expanders - Apply equivalent subjects<br>Narrow by SubjectAge: - child, preschool: 2-5 years<br>Narrow by SubjectAge: - child: 6-12 years<br>Search modes - Proximity	Interface - EBSCOhost Research Databases<br>Search Screen - Advanced Search<br>Database - CINAHL Ultimate
S7	(S1 OR S2) OR (S5 AND S6)	Expanders - Apply equivalent subjects<br>Search modes - Proximity	Interface - EBSCOhost Research Databases<br>Search Screen - Advanced Search<br>Database - CINAHL Ultimate
S6	TI (risk-taking OR "risk taking" OR ((risk not (“at risk” or risk factor*))) OR risk-management OR risk-perception OR safety OR safety-management OR "safety management" OR adventur*) OR AB (risk-taking OR "risk taking" OR ((risk not (“at risk” or risk factor*))) OR risk-management OR risk-perception OR safety OR safety-management OR "safety management" OR adventur*) OR (risk-taking OR "risk taking" OR (	Expanders - Apply equivalent subjects<br>Search modes - Proximity	Interface - EBSCOhost Research Databases<br>Search Screen - Advanced Search<br>Database - CINAHL Ultimate

	(risk not ("at risk" or risk factor*))) OR risk-management OR risk-perception OR safety OR safety-management OR "safety management" OR adventur*)		
S5	S3 OR S4	Expanders - Apply equivalent subjects<br>Search modes - Proximity	Interface - EBSCOhost Research Databases<br>Search Screen - Advanced Search<br>Database - CINAHL Ultimate
S4	play	Expanders - Apply equivalent subjects<br>Search modes - Proximity	Interface - EBSCOhost Research Databases<br>Search Screen - Advanced Search<br>Database - CINAHL Ultimate

S3	TI ((“play and playthings”) OR Playgrounds OR "Childhood play behaviour" OR "Children’s recreational games" OR "Children’s play development" OR "Playspace" OR “play space”) OR AB ((“play and playthings”) OR Playgrounds OR "Childhood play behaviour" OR "Children’s recreational games" OR "Children’s play development" OR "Playspace" OR “play space”) OR ((“play and playthings”) OR Playgrounds OR "Childhood play behaviour" OR "Children’s recreational games" OR "Children’s play development" OR "Playspace" OR “play space”)	Expanders - Apply equivalent subjects<br>Search modes - Proximity	Interface - EBSCOhost Research Databases<br>Search Screen - Advanced Search<br>Database - CINAHL Ultimate
S2	TI (play N3 disappear OR play N3 “get lost” OR risky N3 play OR adventur* N3 play OR (play N3 (unsupervised OR supervision OR without N2 supervision OR unaccompanied OR unescorted OR adult* N2 presen* OR independent OR autonomous)) OR (“independent mobility” AND child*)) OR AB (play N3 disappear OR play N3 “get lost” OR risky N3 play OR adventur* N3 play OR (play N3 (unsupervised	Expanders - Apply equivalent subjects<br>Search modes - Proximity	Interface - EBSCOhost Research Databases<br>Search Screen - Advanced Search<br>Database - CINAHL Ultimate

	OR supervision OR without N2 supervision OR unaccompanied OR unescorted OR adult* N2 presen* OR independent OR autonomous)) OR (“independent mobility” AND child*)) OR (play N3 disappear OR play N3 “get lost” OR risky N3 play OR adventur* N3 play OR (play N3 (unsupervised OR supervision OR without N2 supervision OR unaccompanied OR unescorted OR adult* N2 presen* OR independent OR autonomous)) OR (“independent mobility” AND child*))		
S1	TI (Play N3 Heights OR Play N3 Speed OR Play N3 Tools OR Play N3 “Dangerous element*” OR Play N3 fire OR Play N3 “rough-and-tumble” OR (Play N3 “rough and tumble”) OR ((roughhous* or rough hous*)) OR ((horseplay or “horse play”)) OR Play N3 “wrestling” OR Play N3 “aggress*” OR Play fight*) OR (Play N3 Heights OR Play N3 Speed OR Play N3 Tools OR Play N3 “Dangerous element*” OR Play N3 fire OR Play N3 “rough-and-tumble” OR (Play N3 “rough and tumble”) OR ((roughhous* or	Expanders - Apply equivalent subjects<br>Search modes - Proximity	Interface - EBSCOhost Research Databases<br>Search Screen - Advanced Search<br>Database - CINAHL Ultimate

rough hous*)) OR ((horseplay or “horse play”)) OR Play N3 “wrestling” OR Play N3 “aggress*” OR Play fight*) OR AB (Play N3 Heights OR Play N3 Speed OR Play N3 Tools OR Play N3 “Dangerous element*” OR Play N3 fire OR Play N3 “rough-and-tumble” OR (Play N3 “rough and tumble”) OR ((roughhous* or rough hous*)) OR ((horseplay or “horse play”)) OR Play N3 “wrestling” OR Play N3 “aggress*” OR Play fight*)

EBSCOhost ERIC

EBSCOhost

Thu, June 25, 2026 11:10:15 am

#	Query	Limiters/Expanders	Last Run Via
S15	(S9 OR S10) OR (S13 AND S14)	Expanders - Apply equivalent subjects<br>Search modes - Proximity	Interface - EBSCOhost Research Databases<br>Search Screen - Advanced Search<br>Database - ERIC
S14	TI (risk-taking OR "risk taking" OR ((risk not (“at risk” or risk factor*))) OR risk-management OR risk-perception OR safety OR safety-management OR "safety management" OR adventur*) OR AB (risk-taking OR "risk taking" OR ((risk not (“at risk” or risk factor*))) OR risk-management OR risk-perception OR safety OR safety-management OR "safety management" OR adventur*) OR (risk-taking OR "risk taking" OR ((risk not (“at risk” or risk factor*))) OR risk-management OR risk-perception OR safety OR safety-management OR "safety management" OR adventur*)	Expanders - Apply equivalent subjects<br>Search modes - Proximity	Interface - EBSCOhost Research Databases<br>Search Screen - Advanced Search<br>Database - ERIC
S13	S11 OR S12	Expanders - Apply equivalent subjects	Interface - EBSCOhost Research Databases

		Search modes - Proximity	Search Screen - Advanced Search<br>Database - ERIC
S12	play	Expanders - Apply equivalent subjects<br>Search modes - Proximity	Interface - EBSCOhost Research Databases<br>Search Screen - Advanced Search<br>Database - ERIC
S11	TI ((“play and playthings”) OR Playgrounds OR "Childhood play behaviour" OR "Children’s recreational games" OR "Children’s play development" OR "Playspace" OR “play space”) OR AB ((“play and playthings”) OR Playgrounds OR "Childhood play behaviour" OR "Children’s recreational games" OR "Children’s play development" OR "Playspace" OR “play space”) OR ((“play and playthings”) OR Playgrounds OR "Childhood play behaviour" OR "Children’s recreational games" OR "Children’s play development" OR "Playspace" OR “play space”)	Expanders - Apply equivalent subjects<br>Search modes - Proximity	Interface - EBSCOhost Research Databases<br>Search Screen - Advanced Search<br>Database - ERIC
S10	TI (play N3 disappear OR play N3 “get lost” OR risky N3 play OR adventur* N3 play OR (play N3 (unsupervised OR supervision OR without N2 supervision OR unaccompanied OR unescorted OR adult* N2 presen* OR	Expanders - Apply equivalent subjects<br>Search modes - Proximity	Interface - EBSCOhost Research Databases<br>Search Screen - Advanced Search<br>Database - ERIC

	independent OR autonomous)) OR (“independent mobility” AND child*)) OR AB (play N3 disappear OR play N3 “get lost” OR risky N3 play OR adventur* N3 play OR (play N3 (unsupervised OR supervision OR without N2 supervision OR unaccompanied OR unescorted OR adult* N2 presen* OR independent OR autonomous)) OR (“independent mobility” AND child*)) OR (play N3 disappear OR play N3 “get lost” OR risky N3 play OR adventur* N3 play OR (play N3 (unsupervised OR supervision OR without N2 supervision OR unaccompanied OR unescorted OR adult* N2 presen* OR independent OR autonomous)) OR (“independent mobility” AND child*))		
S9	TI (Play N3 Heights OR Play N3 Speed OR Play N3 Tools OR Play N3 “Dangerous element*” OR Play N3 fire OR Play N3 “rough-and-tumble” OR (Play N3 “rough and tumble”) OR ((roughhous* or rough hous*)) OR ((horseplay or “horse play”)) OR Play N3 “wrestling” OR Play N3 “aggress*” OR Play fight*) OR (Play N3	Expanders - Apply equivalent subjects<br>Search modes - Proximity	Interface - EBSCOhost Research Databases<br>Search Screen - Advanced Search<br>Database - ERIC

	Heights OR Play N3 Speed OR Play N3 Tools OR Play N3 “Dangerous element*” OR Play N3 fire OR Play N3 “rough-and-tumble” OR (Play N3 “rough and tumble”) OR ((roughhous* or rough hous*)) OR ((horseplay or “horse play”)) OR Play N3 “wrestling” OR Play N3 “aggress*” OR Play fight*) OR AB (Play N3 Heights OR Play N3 Speed OR Play N3 Tools OR Play N3 “Dangerous element*” OR Play N3 fire OR Play N3 “rough-and-tumble” OR (Play N3 “rough and tumble”) OR ((roughhous* or rough hous*)) OR ((horseplay or “horse play”)) OR Play N3 “wrestling” OR Play N3 “aggress*” OR Play fight*)		
S8	(S1 OR S2) OR (S5 AND S6)	Expanders - Apply equivalent subjects<br>Narrow by SubjectAge: - child, preschool: 2-5 years<br>Narrow by SubjectAge: - child: 6-12 years<br>Search modes - Proximity	Interface - EBSCOhost Research Databases<br>Search Screen - Advanced Search<br>Database - ERIC
S7	(S1 OR S2) OR (S5 AND S6)	Expanders - Apply equivalent subjects<br>Search modes - Proximity	Interface - EBSCOhost Research Databases<br>Search Screen - Advanced Search<br>Database - ERIC

S6	TI (risk-taking OR "risk taking" OR ((risk not (“at risk” or risk factor*))) OR risk-management OR risk-perception OR safety OR safety-management OR "safety management" OR adventur*) OR AB (risk-taking OR "risk taking" OR ((risk not (“at risk” or risk factor*))) OR risk-management OR risk-perception OR safety OR safety-management OR "safety management" OR adventur*) OR (risk-taking OR "risk taking" OR ((risk not (“at risk” or risk factor*))) OR risk-management OR risk-perception OR safety OR safety-management OR "safety management" OR adventur*)	Expanders - Apply equivalent subjects<br>Search modes - Proximity	Interface - EBSCOhost Research Databases<br>Search Screen - Advanced Search<br>Database - ERIC
S5	S3 OR S4	Expanders - Apply equivalent subjects<br>Search modes - Proximity	Interface - EBSCOhost Research Databases<br>Search Screen - Advanced Search<br>Database - ERIC
S4	play	Expanders - Apply equivalent subjects<br>Search modes - Proximity	Interface - EBSCOhost Research Databases<br>Search Screen - Advanced Search<br>Database - ERIC
S3	TI ((“play and playthings”) OR Playgrounds OR "Childhood play behaviour" OR "Children’s recreational games" OR	Expanders - Apply equivalent subjects<br>Search modes - Proximity	Interface - EBSCOhost Research Databases<br>Search Screen - Advanced Search<br>Database - ERIC

	"Children’s play development" OR "Playspace" OR “play space”) OR AB ((“play and playthings”) OR Playgrounds OR "Childhood play behaviour" OR "Children’s recreational games" OR "Children’s play development" OR "Playspace" OR “play space”) OR ((“play and playthings”) OR Playgrounds OR "Childhood play behaviour" OR "Children’s recreational games" OR "Children’s play development" OR "Playspace" OR “play space”)		
S2	TI (play N3 disappear OR play N3 “get lost” OR risky N3 play OR adventur* N3 play OR (play N3 (unsupervised OR supervision OR without N2 supervision OR unaccompanied OR unescorted OR adult* N2 presen* OR independent OR autonomous)) OR (“independent mobility” AND child*)) OR AB (play N3 disappear OR play N3 “get lost” OR risky N3 play OR adventur* N3 play OR (play N3 (unsupervised OR supervision OR without N2 supervision OR unaccompanied OR unescorted OR adult* N2 presen* OR independent OR autonomous)) OR (“independent	Expanders - Apply equivalent subjects<br>Search modes - Proximity	Interface - EBSCOhost Research Databases<br>Search Screen - Advanced Search<br>Database - ERIC

	mobility" AND child*)) OR (play N3 disappear OR play N3 "get lost" OR risky N3 play OR adventur* N3 play OR (play N3 (unsupervised OR supervision OR without N2 supervision OR unaccompanied OR unescorted OR adult* N2 presen* OR independent OR autonomous)) OR ("independent mobility" AND child*))		
S1	TI (Play N3 Heights OR Play N3 Speed OR Play N3 Tools OR Play N3 "Dangerous element*" OR Play N3 fire OR Play N3 "rough-and-tumble" OR (Play N3 "rough and tumble") OR ((roughhous* or rough hous*)) OR ((horseplay or "horse play")) OR Play N3 "wrestling" OR Play N3 "aggress*" OR Play fight*) OR (Play N3 Heights OR Play N3 Speed OR Play N3 Tools OR Play N3 "Dangerous element*" OR Play N3 fire OR Play N3 "rough-and-tumble" OR (Play N3 "rough and tumble") OR ((roughhous* or rough hous*)) OR ((horseplay or "horse play")) OR Play N3 "wrestling" OR Play N3 "aggress*" OR Play fight*) OR AB (Play N3 Heights OR Play N3 Speed OR	Expanders - Apply equivalent subjects<br>Search modes - Proximity	Interface - EBSCOhost Research Databases<br>Search Screen - Advanced Search<br>Database - ERIC

Play N3 Tools OR Play N3 “Dangerous element*” OR Play N3 fire OR Play N3 “rough-and-tumble” OR (Play N3 “rough and tumble”) OR ((roughhous* or rough hous*)) OR ((horseplay or “horse play”)) OR Play N3 “wrestling” OR Play N3 “aggress*” OR Play fight*)

EBSCOhost SPORTDISCUS

#	Query	Limiters/Expanders	Last Run Via	Results
S7	(S1 OR S2) OR (S5 AND S6)	Expanders - Apply equivalent subjects<br>Search modes - Proximity	Interface - EBSCOhost Research Databases<br>Search Screen - Advanced Search<br>Database - SPORTDiscus	Display
S6	TI (risk-taking OR "risk taking" OR ((risk not (“at risk” or risk factor*))) OR risk-management OR risk-perception OR safety OR safety-management OR "safety management" OR adventur*) OR AB (risk-taking OR "risk taking" OR ((risk not (“at risk” or risk factor*))) OR risk-management OR risk-perception OR safety OR safety-management OR "safety management" OR adventur*) OR KW (risk-taking OR "risk taking" OR ((risk not (“at risk” or risk factor*))) OR risk-management OR risk-perception OR safety OR safety-management OR "safety management" OR adventur*)	Expanders - Apply equivalent subjects<br>Search modes - Proximity	Interface - EBSCOhost Research Databases<br>Search Screen - Advanced Search<br>Database - SPORTDiscus	Display
S5	S3 OR S4	Expanders - Apply equivalent subjects	Interface - EBSCOhost Research Databases	Display

		Search modes - Proximity	Search Screen - Advanced Search<br>Database - SPORTDiscus	
S4	KW play	Expanders - Apply equivalent subjects<br>Search modes - Proximity	Interface - EBSCOhost Research Databases<br>Search Screen - Advanced Search<br>Database - SPORTDiscus	Display
S3	TI ((“play and playthings”) OR Playgrounds OR "Childhood play behaviour" OR "Children’s recreational games" OR "Children’s play development" OR "Playspace" OR “play space”) OR AB ((“play and playthings”) OR Playgrounds OR "Childhood play behaviour" OR "Children’s recreational games" OR "Children’s play development" OR "Playspace" OR “play space”) OR KW ((“play and playthings”) OR Playgrounds OR "Childhood play behaviour" OR "Children’s recreational games" OR "Children’s play development" OR "Playspace" OR “play space”)	Expanders - Apply equivalent subjects<br>Search modes - Proximity	Interface - EBSCOhost Research Databases<br>Search Screen - Advanced Search<br>Database - SPORTDiscus	Display
S2	TI (play N3 disappear OR play N3 “get lost” OR risky N3 play OR adventur* N3 play OR (play N3 (unsupervised OR supervision OR without N2 supervision OR unaccompanied OR unescorted OR adult* N2 presen* OR	Expanders - Apply equivalent subjects<br>Search modes - Proximity	Interface - EBSCOhost Research Databases<br>Search Screen - Advanced Search<br>Database - SPORTDiscus	Display

	independent OR autonomous)) OR (“independent mobility” AND child*)) OR AB (play N3 disappear OR play N3 “get lost” OR risky N3 play OR adventur* N3 play OR (play N3 (unsupervised OR supervision OR without N2 supervision OR unaccompanied OR unescorted OR adult* N2 presen* OR independent OR autonomous)) OR (“independent mobility” AND child*)) OR KW (play N3 disappear OR play N3 “get lost” OR risky N3 play OR adventur* N3 play OR (play N3 (unsupervised OR supervision OR without N2 supervision OR unaccompanied OR unescorted OR adult* N2 presen* OR independent OR autonomous)) OR (“independent mobility” AND child*))			
S1	TI (Play N3 Heights OR Play N3 Speed OR Play N3 Tools OR Play N3 “Dangerous element*” OR Play N3 fire OR Play N3 “rough-and-tumble” OR (Play N3 “rough and tumble”) OR ((roughhous* or rough hous*)) OR ((horseplay or “horse play”)) OR Play N3 “wrestling” OR Play N3 “aggress*” OR Play fight*) OR KW (Play N3	Expanders - Apply equivalent subjects<br>Search modes - Proximity	Interface - EBSCOhost Research Databases<br>Search Screen - Advanced Search<br>Database - SPORTDiscus	Display

Heights OR Play N3 Speed OR Play N3 Tools OR Play N3 “Dangerous element*” OR Play N3 fire OR Play N3 “rough-and-tumble” OR (Play N3 “rough and tumble”) OR ((roughhous* or rough hous*)) OR ((horseplay or “horse play”)) OR Play N3 “wrestling” OR Play N3 “aggress*” OR Play fight*) OR AB (Play N3 Heights OR Play N3 Speed OR Play N3 Tools OR Play N3 “Dangerous element*” OR Play N3 fire OR Play N3 “rough-and-tumble” OR (Play N3 “rough and tumble”) OR ((roughhous* or rough hous*)) OR ((horseplay or “horse play”)) OR Play N3 “wrestling” OR Play N3 “aggress*” OR Play fight*)

Set	Search Statement OVID MEDLINE
1.	(Play adj3 Height*).ab,kf,ti.
2.	(Play adj3 Speed*).ab,kf,ti.
3.	(Play adj3 Tools).ab,kf,ti.
4.	(Play adj3 "Dangerous element*").ab,kf,ti.
5.	(Play adj3 fire).ab,kf,ti.
6.	(Play adj3 "rough-and-tumble").ab,kf,ti.
7.	(roughhous* or rough hous*).ab,kf,ti.
8.	(horseplay or "horse play").ab,kf,ti.
9.	(Play adj3 "wrestling").ab,kf,ti.
10.	(Play adj3 "aggress*").ab,kf,ti.
11.	Play fight*.ab,kf,ti.
12.	(play adj3 disappear).ab,kf,ti.
13.	(play adj3 "get lost").ab,kf,ti.
14.	(risky adj3 play).ab,kf,ti.
15.	(adventur* adj3 play).ab,kf,ti.
16.	(play adj3 (((((unsupervised or supervision or without) adj2 supervision) or unaccompanied or unescorted or adult*) adj2 presen*) or independent or autonomous)).ab,kf,ti.
17.	(" independent mobility" and child*).ab,kf,ti.
18.	1 or 2 or 3 or 4 or 5 or 6 or 7 or 8 or 9 or 10 or 11 or 12 or 13 or 14 or 15 or 16 or 17
19.	("play and playthings" or Playgrounds or "Childhood play behaviour" or "Children's recreational games" or "Children's play development" or "Playspace" or "play space").ab,kf,ti.
20.	play.kf.
21.	19 or 20
22.	(risk-taking or "risk taking" or (risk not ("at risk" or risk factor*)) or risk-management or risk-perception or safety or safety-management or "safety management" or adventur*).ab,kf,ti.
23.	21 and 22
24.	18 or 23

Set	Search Statement OVID PsychInfo
1.	(Play adj3 Height*).ab,id,ti.
2.	(Play adj3 Speed*).ab,id,ti.
3.	(Play adj3 Tools).ab,it,ti.
4.	(Play adj3 "Dangerous element*").ab,id,ti.
5.	(Play adj3 fire).ab,id,ti.
6.	(Play adj3 "rough-and-tumble").ab,id,ti.
7.	(roughhous* or rough hous*).ab,id,ti.
8.	(horseplay or "horse play").ab,id,ti.
9.	(Play adj3 "wrestling").ab,id,ti.
10.	(Play adj3 "aggress*").ab,id,ti.
11.	Play fight*.ab,id,ti.
12.	(play adj3 disappear).ab,id,ti.
13.	(play adj3 "get lost").ab,id,ti.
14.	(risky adj3 play).ab,id,ti.
15.	(adventur* adj3 play).ab,id,ti.
16.	(play adj3 (((((unsupervised or supervision or without) adj2 supervision) or unaccompanied or unescorted or adult*) adj2 presen*) or independent or autonomous)).ab,id,ti.
17.	(" independent mobility" and child*).ab,id,ti.
18.	1 or 2 or 3 or 4 or 5 or 6 or 7 or 8 or 9 or 10 or 11 or 12 or 13 or 14 or 15 or 16 or 17
19.	("play and playthings" or Playgrounds or "Childhood play behaviour" or "Children's recreational games" or "Children's play development" or "Playspace" or "play space").ab,id,ti.
20.	play.id.
21.	19 or 20
22.	(risk-taking or "risk taking" or (risk not ("at risk" or risk factor*)) or risk-management or risk-perception or safety or safety-management or "safety management" or adventur*).ab,id,ti.
23.	21 and 22
24.	18 or 23
