# Supplementary material for: Outdoor risky play and health-related outcomes in children aged 3–12 years: A mixed-methods systematic review
Source: PLoS One. 2026 Oct 26;21(10):e0347444. doi: 10.1371/journal.pone.0347444 (PMC13704885; doi:10.1371/journal.pone.0347444)
Supplement: Supplementary file 2 [file pone.0347444.s002.docx]

# **S2 Appendix: Overview of Included Studies**

Table S2-1: Studies contributing to quantitative analysis (n=53)

| **Lead author (year)** | **Study Overview** | **Exposure(s)** | **Outcome(s)** | **Quantitative measures of effect** | **Overall effect on health** |
| --- | --- | --- | --- | --- | --- |
| Adams et al. (2018) (1) | Cross-sectional study in Melbourne, Australia objectively measuring the activity levels, functional movement skills and equipment used during play of children aged 5-10 years (n=57) in a variety of community playground settings. | *Risky Play Supportive Environments*  Playground type/ design (traditional, contemporary, or adventure) | *Physical Outcome*  a. Accelerometers (15 s epoch):   i. MPA (mean minutes)   ii. VPA (mean minutes)   iii. MVPA (mean minutes)   b. Fundamental motor skills from observations using the SOFIT instrument | ai. The GLM identified a significant association (p=0.03) in time spent in MPA by playground. Post-hoc tests (not shown) showed that children spent significantly more time in MPA at the traditional playground compared to the adventure playground (2.72 mean difference, p=0.023)  aii. p>0.05, stats NR  aiii. p>0.05, stats NR   b. F2,54 =0.2, p=0.855 | Minority negative effect on health |
| Aggio et al. (2017) (2) | Cross-sectional study in the United Kingdom investigating associations between physical activity, demographic, behavioural, environmental, and social correlates and children aged 7 years (n=13,169) independent outdoor play. | *Independent Mobility*  Independent mobility, reported by parents | *Physical Outcome*  Accelerometers (15s epoch):  a. sedentary (<100 cpm)  b. MVPA (>2241 cpm)  c. Steps (per day) | β (95% CI)  a. −4.91 (−7.54, −2.29)  b. 2.21 (1.09, 3.34)  c. 336.66 (209.80, 463.51) | Consistently positive effect on health |
| Aliyas et al. (2024) (3) | Cross-sectional study to quantify the levels of independent mobility (IM), active transportation (AT), and free play (FP) among children aged 6-12 years (n=537) residing in high- and low-SES neighbourhoods in Montreal & Kingston, Canada. | *Independent Mobility*  Independent mobility, reported by children using a previously validated mobility licences’ scale | *Physical Outcome*  Active transportation, self-reported by children | a. Low SES: r = 0.291  High SES: r = 0.284  b. Low SES: r = 0.426  High SES: r = 0.121   (statistical significance not reported, CIs not reported) | Some positive effect on health |
| Branje et al. (2021) (4) | Explored the impact of a child care-based outdoor loose parts play intervention on preschoolers' aged 3-5 years (n=197) fundamental movement skills using a multi-method, clustered, randomized control trial (RCT) design in Nova Scotia, Canada. | *Risky Play Supportive Environments*  Intervention that integrated loose parts into the outdoor environment of regulated childcare centres | *Physical Outcome*  The TGMD-3 was used to assess:  a. total locomotor skills  b. total object control skills  c. total fundamental movement score   The PGMQ was used to assess:  d. total balance control score | Multi-level modelling showed group (intervention, control) to be non-significant in each model  a. p= 0.99, other stats NR  b. p= 0.18, other stats NR  c. p= 0.51, other stats NR  d. p= 0.08, other stats NR | No consistent evidence of effect on health |
| Brussoni et al. (2017) (5) | Convergent mixed methods repeated measures study to investigate the effects of natural risky play environments on children's aged 2-5 years (n=45) health and well-being in two child care centres in Vancouver, Canada. | *Risky Play Supportive Environments*  Intervention based on seven Cs (repeated measures design, T1 and T2) | *Mental Health & Well-being* Outcome  Strengths and Difficulties Questionnaire (teacher report):  a. emotional symptoms subscale  b. conduct problems subscale  c. hyperactivity/inattention subscale    Preschool Social Behaviour Scale (teacher report):  d. depressed affect subscale. | a. n.s. no statistics provided)  b. n.s. no statistics provided)  c. n.s. (no statistics provided)  d. Median  T1=6.0, T2=3.0; z=2.24, p=0.03. | Minority positive effect on health |
|  |  |  | *Physical Outcome*  Accelerometers: MVPA (³420 count/15s) | *M* decrease = 1.32 min, *SE* = 0.37, *p* < 0.001 | Consistently negative effect on health |
|  |  |  | *Aggression Outcome*  Preschool Social Behaviour Scale (teacher report):  a. relational aggression subscale  b. overt aggression subscale | a. n.s. no statistics provided)  b. n.s. no statistics provided) | No clear evidence of effect on health |
|  |  |  | *Social* *Outcome*  Strengths and Difficulties Questionnaire (teacher report) peer problems subscale | Median T1= 2.3, T2= 2.0;  z = 2.10, p = 0.036 | Consistently positive effect on health |
| Bundy et al. (2009) (6) | Non-randomised experimental study investigated a primary school intervention to promote active, social, and creative play through the addition of loose parts for children aged 5-7 years (n=12) to play with within their school ground in Western Sydney, Australia. | *Risky Play Supportive Environments*  Intervention consisting of introducing ‘loose parts’, or ‘scrounge materials’ to the school playground for a term and a half (11 weeks of school time) during winter. | *Physical Outcome*  Accelerometers (60 s epoch):  Mean PA counts at lunchtime | Mean PA counts increased from 1028 (SD=770) to 1612 (SD= 491); Wilcoxon signed rank test (one-tailed) p=0.014; es = 0.9 SD | Consistently positive effect on health |
| Bundy et al. (2017) (7) | Randomised controlled trial in Sydney, Australia that aimed to promote play through an intervention that placed recycled materials with no obvious play value on school playgrounds and examined effects on physical activity, social skills, and perceived competence/social acceptance of 5 to 7-yeard-old children (n=113 intervention group). | *Risky Play Supportive Environments*  Cluster RCT, 12 schools allocated to intervention or control. Intervention; 13-week playground intervention where recycled materials were placed on school playgrounds for use at each breaktime, and parents and teachers were invited to participate in a 2-hour risk reframing workshop | *Social* *Outcome*  a) Time spent in play and social interactions i) play ii) social interactions  b) Pictorial Scale of Perceived Competence and Social Acceptance for Young Children (PSPCSAYC) used to assess:  i) child perceived peer acceptance  ii) teacher perceived peer acceptance   c)Social Skills Improvement System Rating Scale used to assess:  i) parent reported social skills ii) teacher reported social skills | ai) Coefficient: 11.8%, 95% CI: −1.3-24.8, p=.08 aii) Coefficient: 3.80%, 95% CI: −3.6-11.2, p=.31 bi) Coefficient: -0.13, 95% CI: −0.29-0.28, p=.11  bii) Coefficient: -0.13, 95% CI: −0.33-0.07, p=.19 ci) Coefficient: ns cii) Coefficient: 2.02, 95% CI: -0.98-5.02, p=.19 | No consistent evidence of effect on health |
| Cho et al. (2023) (8) | Cross-sectional study to determine the role of children aged 3-5 years (n=862) self-control in the relationship between risky play attempts and injury experiences in a naturalistic environment in Korea. | *General Risky Play*  1. Number of risky play types endorsed on TRiPS.  2. Naturalistic setting visit frequency (teacher report)  3. Duration of stay in nature per visit (teacher report)  4. Naturalistic setting visit frequency (parent report)  5. Duration of stay in nature per visit (parent report) | *Mental Health & Well-being Outcome*  Self-control ability (leadership scale, adapted. Mother report) | Correlations:   1. r = .02, n.s.  2. r = .11, p < .01  3. r = .01, n.s.  4. r = .17, p <.001  5. r = .19, p < .01 | Some positive effect on health |
|  |  |  | *Risk Competence and Injury Outcome*  Injury experience (parent report). Assessed via Injury History Checklist. | Regression (all in same model controlling for gender, age, harm avoidance, self-control).  1. Beta = .12, p <.01  2. Beta = -.02, n.s.  3. Beta = .08, p <.05  4. Beta = .07, p <.05  5. Beta = .28, p < .001    Significant interaction between the number of play types (1) and self-control (β  = .−.08, p < .01). Greater injury experience found at higher frequencies of risky play, in those with low self-control. For those with high self-control, difference were negligible. | Some negative effect on health |
| Colwell and Lindsey (2005) (9) | Cross-sectional study to examine associations between preschool children's aged 4-6 years (n=60) pretend and physical play with same-sex, other-sex, and mixed-sex peers and children's social competence with peers in the United States. | *Rough & Tumble*  Rough and tumble play:  1. Same sex peers  2. Mixed sex peers | *Social Outcome*  a) Children's classroom peer acceptance (as rated by same sex peers)  b) Children's classroom peer acceptance (as rated by other sex peers)  c) teacher-rated social competence | GIRLS:  1a) r = 0.05, n.s.  1b) r = 0.04, n.s.  1c) r = 0.01, n.s.  BOYS:  1a) r = 0.31, p<0.05  1b) r = 0.14, n.s.  1c) r = 0.28, p<0.05   GIRLS:  2a) r = -0.13, n.s.  2b) r = -0.09, n.s.  2c) r = -0.07, n.s.   BOYS:  2a) r = -0.28‚ p<0.05  2b) r = -0.32‚ p<0.05  2c) -0.30‚ p<0.05 | Minority negative effect on health |
| De Meester et al. (2014) (10) | Cross-sectional study in Flanders, Belgium to examine the associations of parental perceptions of neighborhood environmental attributes with active transport and total physical activity in 10-12 year old children (n=736) and the potential mediating effect of independent mobility on these associations. | *Independent Mobility*  Independent mobility, reported by parents | *Physical Outcome*  Child-completed Flemish Physical Activity Questionnaire (min/d):  a. duration of school-related active transport  b. walking for transport during leisure time  c. cycling for transport during leisure time  d. total physical activity   Pedometer or accelerometer:  e. daily step counts (steps/d) | β (95% CI)  Girls:  a. 0.057 (0.012 – 0.102), p<0.05  b. 0.037 (−0.012 – 0.085), p>0.05  c. p > .05, stats NR  d. p > .05, stats NR  e. 0.051 (0.033 – 0.06), p<0.05  Boys:  a. p > .05, stats NR  b. p > .05, stats NR  c. p > .05, stats NR  d. p > .05, stats NR  e. p > .05, stats NR | Minority positive effect on health |
| Dodd et al. (2023) (11) | Cross-sectional study in Northern Ireland & United Kingdom to examine associations between the time that children aged 5-11 years (n=427 & 1,919) spent playing adventurously and their mental health. | *General Risky Play*  Total time spent playing adventurously (parent report) | *Mental Health & Well-being* *Outcome*  Parent report of:  a) Strengths and Difficulties Questionnaire (SDQ) Internalising problems  b) Strengths and Difficulties Questionnaire (SDQ) Externalising problems  c) Positive Affect  d) Negative Affect | Study 1  a) r=-.19 [-0.28, ‚-0.10], p < .0125  b) r = -.02 [-0.11, 0.08], n.s.  c) 0.12 [0.03, 0.22], p < .0125  d) r = 0.02 [-0.11, 0.08], n.s.  Study 2  a) r=-0.06 [-0.11, -0.02], p < .0125  b) r=0.04 [-0.01, 0.08], n.s.  c) r=0.18 [0.13, 0.22], p < .0125  d) 0.04 [-0.01, 0.09], n.s. | Some positive effect on health |
| Engelen et al. (2013) (12) | Randomised controlled trial that tested the effectiveness of a modification of school playground and adult intervention programme on physical activity of children aged 5-7 years (n=221) in Sydney, Australia. | *Risky Play Supportive Environments*  Introduced loose, primarily recycled materials on the school playgrounds to be used during all break times | *Physical Outcome*  Accelerometers (15 s epoch):  a. Break time PA     i. Sedentary (min; 0-100 cpm)     ii. LPA (min; 101-2295 cpm)     iii. MVPA (min; >2296 cpm)     iv. Total counts x 1000   b. Whole school day PA     i. Sedentary (min; 0-100 cpm)    ii. LPA (min; 101-2295 cpm)      iii. MVPA (min; >2296 cpm)     iv. Total counts x 1000 | β (95% CI)  ai. -2.13 (-3.8, -0.5), p=0.01  aii. 0.17 (-1.1, 1.4), p=0.79  aiii. 1.82 (0.5, 3.1), p=0.006  aiv. 9.35 (3.5, 15.2), p=0.002   bi. 0.04 (-6.0, 6.1), p=0.99  bii. -4.03 (-9.4, 1.4), p=0.14  biii. 1.55 (-0.8, 3.9), p=0.19  biv. 4.61 (-143, 116.3), p=0.49 | Minority positive effect on health |
| Farmer et al. (2017) (13) | Randomised controlled trial to determine whether increasing opportunities for risk and challenge within the school playground influence children aged 6-9 years (n=840) interactions with one another, particularly negative interactions like bullying, in Otago & Auckland regions of New Zealand. | *Risky Play Supportive Environments*  PLAY intervention (cluster RCT) | *Social Outcome*  Child report of exclusion during playtime | 1 year follow-up: OR = 1.11 (95% CI 0.72 – 1.70), p = .64  2 year follow-up: OR = 1.14 (95% CI 0.69 – 1.90, p=.61 | No consistent effect on health |
|  |  |  | *Mental Health & Well-being Outcome*  Child report of happiness in school. | 1 year follow-up: OR = 1.03 (95% CI 0.71 – 1.49), p = .88   2 year follow-up: OR = 1.64 (95% CI 1.20-2.25), p=0.02 | Some positive effect on health |
| Farmer et al. (2017) (14) | Randomised controlled trial to determine whether providing greater opportunities for risk and challenge in primary schools in Otago & Auckland regions of New Zealand increased children aged 6-9 years (n=840) physical activity and reduced relative body weight over the long term. | *Risky Play Supportive Environments*  Condition within RCT design (intervention vs control). Each intervention school was provided with a tailored plan to improve their play space. This could include the addition of more interactive play equipment, and alterations to school rules and policies that may limit risk-taking during play. | *Physical Outcome*  Accelerometers (15 s epoch):  cpm:  a. whole day  b. school day  c. break time  d. lunch time    MVPA (min):  e. whole day  f. school day  g. break time  h. lunch time    i. BMI  j. BMI z-score  k. waist circumference (cm)  l. waist-to-height ratio | Adjusted difference between conditions (95% CI)   1 year:  a. − 6 (−37, 24) p=0.687  b. − 21 (−56, 14) p=0.243  c. 21 (−71, 112) p=0.661  d. 35 (−51, 120) p=0.430  e. 0.3 (−3.2, 3.7) p=0.881  f. − 1.1 (−3.8, 1.6) p=0.415  g. 0.1 (−0.4, 0.5) p=0.697  h. 0.2 (−0.6, 1.0) p= 0.649  i. 1.0 (1.0, 1.0) p= 0.333  j. − 0.02 (−0.06, 0.02) p=0.337  k. − 0.3 (−1.0, 0.4) p=0.424  l. − 0.00 (−0.01, 0.00) p=0.404    2 years:  a. 28 (−66, 10) p= 0.146  b. − 63 (−107, − 20) p=0.005  c. − 5 (−132, 122) p= 0.943  d. − 44 (−164, 75) p=0.467  e. − 3.8 (−7.1, − 0.5) p=0.022  f. − 3.3 (−6.1, − 0.6) p=0.017  g. 0.0 (−1.0, 1.0) p= 0.978  h. 0.4 (−1.1, 2.0) p= 0.588  i. 1.0 (1.0, 1.0) p= 0.435  j. 0.02 (−0.04, 0.07) p=0.567  k. − 0.4 (−1.1, 0.4) p=0.325  l. −0.00 (−0.01, 0.00) p=0.321 | Minority negative effect on health |
| Finegan et al. (1991) (15) | Cross-sectional study examining the relation between Play Activity Questionnaire factor scores and children aged 7 years (n=108) measures of activity level, aggression, and sex-differentiated play in Canada. | *Rough & Tumble*  Rough and Tumble play as assessed on the Play Activity Questionnaire (mother-reported) | *Aggression Outcome*  a) Aggression (assessed via the Child Behaviour Scale, parent report)  Leifer-Roberts conflict situations (child report):  b) Physical aggression  c) Verbal aggression | a) Boys: r = .36, *p* < .0005; Girls: r = .15, n.s.  b) Boys: r = -0.08, n.s.; Girls: r = 0.04, n.s.  c) Boys: r= .02, n.s.; Girls: r = 0.26, p < .05 | Minority negative effect on health |
|  |  |  | *Cognitive* *Outcome*  a. Mother reported activity level, Conners Abbreviated Rating Scale  b. Mother reported activity level, Werry-Weiss-Peters Activity Scale | a. Boys: r=0.42, p<0.005  Girls: r= 0.25, p<0.05    b. Boys: r= 0.35, p<0.05  Girls: r= 0.24, p<0.05 | Consistently negative effect on health |
| Flanders et al. (2009) (16) | Cross-sectional study in Québec, Canada to test the hypothesis that among father-child (aged 2-6 years) play dyads (n=85) in which the father is more dominant, the frequency of RTP interactions should be associated with less aggressive behavior. | *Rough & Tumble*  Father report of rough and tumble play frequency using the 'Pere-En-Jeux' questionnaire | *Aggression* *Outcome*  Father report of physical aggression via the Behavior Questionnaire | Main effect: r= 0.28, p<0.05  (but note interaction between RTP and father dominance, β =.29, t (76) =2.70, P =.01 For low dominance fathers (but not average or high dominance), more rough and tumble play is associated with more physical aggression (Beta = .29, p = .04). | Minority negative effect on health |
| Flanders et al. (2010) (17) | Longitudinal study to test the hypothesis that higher frequencies of father-child (n=34) RTP at preschool age (aged 2-6 years) would continue to be associated with greater physical aggression and worse emotion regulation abilities at school age, among Québec, Canada children whose fathers were less dominant play partners RTP. | *Rough & Tumble*  Father report of rough-and-tumble play frequency | *Mental Health & Well-being* *Outcome*  Father report on Emotion Regulation Checklist (ERC) | Sequential regression analysis controlling for other play and time with child variables, Beta = -0.27, *p*= 0.14. (but note interaction between RTP frequency and dominance, Beta = .39, *p*=.03. For low dominance fathers, more rough and tumble play is associated with poorer emotion regulation (Beta = -.62, *p* < .01). | Minority negative effect on health |
|  |  |  | *Aggression Outcome*  Aggression, as assessed by father report on subscale of the National Longitudinal Study of Children and Youth (NLSCY) Behavior Questionnaire | Sequential regression analysis controlling for other play and time with child variables, Beta = -0.31, *p*= 0.1. (but note interaction between RTP frequency and dominance, Beta = -.39, *p*=.04. For low dominance fathers (but not average or high dominance), more rough and tumble play is associated with more physical aggression (Beta = .69, *p* < .01). | Minority negative effect on health |
| Fliek et al. (2015) (18) | Cross-sectional study to investigate the relationship between parental rough-and-tumble play and children’s aged 2-6 years (n=105) anxiety symptoms in Flemish, Belgium. | *Rough & Tumble*  The Parental Play and Care Questionnaire (PPCQ):   1. Mothers rough-and-tumble play  2. Fathers rough-and-tumble play | *Mental Health & Well-being Outcome*  The Preschool Anxiety Scale-Revised | 1. r= 0.00, n.s.  2. r = 0.11, n.s. (but note that for fathers, father anxiety significantly interacted with 2 (Beta = -.23, p < .05) children displayed higher levels of anxiety symptoms when their low trait anxious fathers displayed higher levels of R&T play). | Minority negative effect on health |
| Freeman and Robinson (2022) (19) | Cross-sectional study in Australia to understand the importance of father-child (n=30) rough and tumble play to the development of children aged 4-7 years executive function, working memory. | *Rough & Tumble*  Fathers were asked to answer a question as to how often they participate in RTP with the study child using a 4-point scale (1 = not at all; 4 = every day). | *Cognitive Outcome*  a. The Wechsler Preschool and Primary Scale of Intelligence, Fourth Edition Australian and New Zealand Standardised Edition (WPPSI-IV) was used to measure children’s working-memory ability. b. The Behaviour Rating Inventory of Executive Function (BRIEF) parent form was used as a behavioural measure of children’s working-memory problems. | a. r = 0.53, *p* = 0.003  b. r =−0.42, p = 0.020 | Consistently positive effect on health |
| Garcia-Cervantes et al. (2016) (20) | Cross-sectional study to investigate the association between independent mobility for walking, cycling, and taking public transport with objectively measured nonschool PA on week- and weekend days in children (n=1,376) aged 8-18 years in Madrid & Cadiz, Spain. | *Independent Mobility*  Independent mobility, assessed through child self-report surveys.    Categorised into:  1. IM walk  2. IM cycling  3. IM transport | *Physical Outcome*  Accelerometers (10 s epoch; MVPA ≥ 2000 cpm)   a. non-school weekday PA   i. MVPA   ii. Total PA   b. weekend PA   i. MVPA   ii. Total PA | Multivariate analyses - β ± SE (note, associations that were not significant in bivariate analyses were not included in multivariate analyses and are therefore reported as p > 0.05, stats NR).   1ai. 4th grade: p > 0.05, stats NR  7th grade: 0.018 ± 0.008, p<0.05  10th grade: 0.018 ± 0.010, p>0.05  1aii. 4th grade: 0.010 ± 0.007, p>0.05  7th grade: 0.012 ± 0.007, p<0.10  10th grade: 0.001 ± 0.017, p>0.05  1bi. 4th grade: p > 0.05, stats NR  7th grade: p > 0.05, stats NR  10th grade: p > 0.05, stats NR  1bii. 4th grade: p > 0.05, stats NR  7th grade: p > 0.05, stats NR  10th grade: p > 0.05, stats NR  2ai. 4th grade: p > 0.05, stats NR  7th grade: p > 0.05, stats NR  10th grade: 0.054 ± 0.014, p<0.001  2aii. 4th grade: p > 0.05, stats NR  7th grade: p > 0.05, stats NR  10th grade: 0.053 ±0.013, p<0.001  2bi. 4th grade: p > 0.05, stats NR  7th grade: p > 0.05, stats NR  10th grade: p > 0.05, stats NR  2bii. 4th grade: p > 0.05, stats NR  7th grade: p > 0.05, stats NR  10th grade: p > 0.05, stats NR  3ai. 4th grade: p > 0.05, stats NR  7th grade: 0.006 ± 0.007, p>0.05  10th grade: p > 0.05, stats NR  3aii. 4th grade: p > 0.05, stats NR  7th grade: 0.004 ± 0.006, p>0.05  10th grade: p > 0.05, stats NR  3bi. 4th grade: p > 0.05, stats NR  7th grade: p > 0.05, stats NR  10th grade: p > 0.05, stats NR  3bii. 4th grade: p > 0.05, stats NR  7th grade: -0.0015 ± 0.010, p>0.05  10th grade: p > 0.05, stats NR | Minority positive effect on health |
| Hamamci and Dagal (2022) (21) | Cross-sectional study to explore the role of executive function and emotion regulation as major components of self-regulation on children's (5 years) (n=127) natural play behaviour in Instanbul, Turkey. | *Rough & Tumble*  Rough and tumble play as reported via the Play behaviour scale for children (teacher report) | *Mental Health & Well-being Outcome*  The Emotional Regulation Checklist, total emotion regulation score (teacher report) | r = 0.463, *p* < .01 | Consistently positive effect on health |
|  |  |  | *Cognitive Outcome*  Teacher report using:  a. The Childhood Executive Functioning Inventory (CHEXI) – working memory  b. The Childhood Executive Functioning Inventory (CHEXI) – inhibitory control | a. r = -0.223, *p* < .05  b. r = -0.300, p < .01 | Consistently negative effect on health |
| Hart et al. (1992) (22) | Cross-sectional study to assess the linkages among children aged 3-5 years (n=136) rough & tumble play with prosocial and antisocial behaviours, along with parental disciplinary strategies in Southern United States. | *Rough & Tumble*  Observation of frequency of rough and tumble play during playground free play | *Social Outcome*  Outcome expectation interview (unfriendly-assertive responses to hypothetical scenarios) | F = 0.91, *p*=0.342 | No consistent evidence of effect on health |
| Huang et al. (2024) (23) | Cross-sectional study exploring IM by sociodemographic factors and type of neighborhoods and its association with PA among children aged 8-12 years (n=330) in Hong Kong. | *Independent Mobility*  (1) IM license: parents’ license granted to their children to travel independently and play outdoors without adults measured by the degree to which parents allowed their children to travel or play outdoors independently without an adult. (2) IM school: the actual mobility behavior through child self-report on school-related destinations. (3) IM non-school: the actual mobility behavior through child self-report on destinations other than school. (4) IM outdoor: the actual behavior through child self-report on outdoor play. | *Physical Outcome*  Light PA, MVPA and ST (in both weekdays and weekend days) were assessed using data from accelerometer data integrated into 15 s as epoch. | (1) Weekdays -light PA: ns Weekdays - MVPA: ns Weekdays-ST: ns Weekend - light PA: ns Weekend-MVPA: 1.33 (0.15-2.51) Weekend-ST: ns (2) Weekdays -light PA: ns Weekdays - MVPA: 1.14 (0.10-2.18) Weekdays-ST: ns Weekend - light PA: ns Weekend-MVPA: ns Weekend-ST: ns (3) Weekdays -light PA: ns Weekdays - MVPA: ns Weekdays-ST: ns Weekend - light PA: ns Weekend-MVPA: ns Weekend-ST: ns (4) Weekdays -light PA: ns Weekdays - MVPA: ns Weekdays-ST: ns Weekend - light PA: ns Weekend-MVPA: ns Weekend-ST: ns | Minority positive effect on health |
| Imai et al. (2022) (24) | Cross-sectional study to examine the relationship between risky play and prosocial behavior, as well as difficulties in daily life, among children aged 67.9-74.9 months (n=32) in Tokyo, Japan. | *General Risky Play*  Presence of risky play during observation of free play across three days at a public kindergarten (present vs. absent binary variable) | *Social Outcome*  Strengths and Difficulties Questionnaire Prosocial behaviour scale | Risky play predicting prosocial score (no covariates): F(1,29) =0.84, p=0.37, n2=0.03   With gender and type of employment as covariates: F(1,27) =6.11, p=0.02, n2=0.23    With gender, type of employment and MVPA as covariates: F(1,22)=5.76, p=0.03, n2=0.26 | Some positive effect on health |
| Kirby et al. (2011) (25) | Longitudinal study investigating parental and peer influences on physical activity, examining gender and developmental differences during early-mid adolescence at aged 11 years (n=641) in cities across Scotland. | *Independent Mobility*  Independent mobility, assessed through child self-report surveys | *Physical Outcome*  Physical activity, assessed with the PAQ-C in the following age groups:  a. P7 (final year of primary school  b. S2 (second year of secondary school  c. S4 (fourth year of secondary school) | OR (95% CI)   a. Boys: 2.44 (1.10, 5.41), p<0.05  Girls: p > .05, stats NR  b. Boys: p > .05, stats NR  Girls: 4.50 (1.95, 10.4), p<0.05  c. Boys: p > .05, stats NR  Girls: p > .05, stats NR | Minority positive effect on health |
| Larouche et al. (2020) (26) | Cross-sectional study to investigate the relationships among independent mobility, active transportation, and physical activity among children in grades 4-6 (n=76) in 3 diverse regions of Canada. | *Independent Mobility*  Independent mobility index computed from children’s responses to a validated mobility questionnaire | *Physical Outcome*  a. Number of active trips, self-reported by children   b. Volume of active travel to/from school (km), assessed by children drawing their usual route to school on an individualised map   c. Pedometer assessed steps/day, daily step counts averaged across the week (valid day 1000-29,999 steps) | IRR (95%CI)   a. overall: 1.09 (1.06-1.12), p<0.001  girls: 1.08 (1.04 to 1.12), p<.001  boys: 1.11 (1.07 to 1.15), p <.001   b. overall: 1.19 (1.15 to 1.24), p<0.001  girls: 1.18 (1.12 to 1.25), p<0.001  boys: 1.23 (1.16 to 1.29), p<0.001   β (95% CI)  c. overall: 147 (33 to 261), p=.011  girls: 74 (-70 to 218), p= .311  boys: 153 (-22 to 328), p=.086 | Some positive effect on health |
| Larouche et al. (2024) (27) | Cross-sectional study to examine the relationship between children's independent mobility and mental health among children aged 7- to 12-years (n=2,291 parents surveyed) in Canada. | *Independent Mobility*  Independent mobility index computed from parent (allowed to roam) and child (independent mobility) responses to two validated mobility questionnaires. | *Mental Health & Well-being Outcome*  Parent-perceived child distress assessed using the distress thermometer. | a. Compared to children allowed to roam <5 min, children allowed to roam 5–15 min (OR=0.76; 95% CI: 0.67–0.88) or >15-minutes (OR=0.61; 95% CI: 0.51–0.72) had lower odds of elevated distress.  b. No associations between child independent mobility licenses and distress (OR=0.94; 95% CI: 0.82–1.08). | Some positive effect on health |
| Lavrysen et al. (2017) (28) | Non-randomised experimental study to examine risk competence within the reality of a class context in young children between 3-8 years (n=76) in Flanders, Belgium. | *General Risky Play*  Intensive package of risky-play activities administered over a three-month period to two classes (four- and six-year-olds) and two age-matched classes served as controls. | *Mental Health & Well-being* *Outcome*  Teacher rated self-esteem using the Bonner questionnaire. | A significant interaction effect between Test Moment and Group (F_1,79_ = 19.88, p < .001). Self-esteem rated higher at post-test, particularly in experimental group. | Consistently positive effect on health |
|  |  |  | *Physical Outcome*  Teacher rated motor control using the Bonner questionnaire. | No significant interaction effect between Test Moment and Group (p > .05, stats not reported). | No consistent effect on health |
|  |  |  | *Cognitive* *Outcome*  Teacher rated concentration using the Bonner questionnaire. | Interaction effect between Test Moment and Group (F1,79 = 4.23, p < .05) revealed that the increase in concentration after the intervention period was greater in the experimental than in the control groups. | Consistently positive effect on health |
|  |  |  | *Risk Competence & Injury Outcome*  a. Reaction time on risk perception test  b. Correct answers on risk perception test  c. observed risk competence | a. 4-way interaction Test Moment × Stimulus Kind × Group × Age (F1,72 = 3.99, p < .05). Four year olds in experimental group showed effect of intervention, this was not obvious in six year olds.  b. Interaction Group × Test Moment (F1,72 = 6.569, p < .05). The experimental group improved from pre- to post-test (75.3 + 15.5% to 79 + 14.5%) whereas the control group did not show an improvement (81.7 + 16 to 78.9 + 14.8%).   c. F1,26 = 3.18, p= 0.086. | Some positive effect on health |
|  |  |  | *Social Outcome*  a. Teacher rated social competence using the Bonner questionnaire.   b. (Low) conflict sensitivity | a. No significant interaction effect between Test Moment and Group (p > .05, stats not reported).   b. interaction effects between Test Moment × Group (F1,79 = 8.26, p < .01) and Test Moment × Group × Age (F1,79 = 5.97, p < .05). The decrease in conflict sensitivity from pre- to post-test was more evident in the experimental group and more so for six- than four-year-olds. | Minority positive effect on health |
| Leung and Loo (2017) (29) | Case report in Hong Kong to study children aged 6-12 years (n=281) travel behaviour for journeys to and from school and all scheduled activities, and examine children's subjective feelings of their happiness of different experiences (momentary well-being) and in the domains of school and life in general (overall well-being), in terms of children's age, gender and travel characteristics. | *Independent Mobility*  Parent report of:  1. licence to cross roads  2. independent travel from school | *Mental Health & Well-being Outcome*  Child report of happiness with life in general | Linear regression controlling for age, gender and other factors:  1. Beta = -3.072, p = .002  2. Beta = .707, p = 0.480 | Some negative effect on health |
| Lindsey and Colwell (2013) (30) | Longitudinal study in Texas, United States to gain a better understanding of children aged 4-6 years (n=122) affective social competence (ASC) by exploring how different play forms, including rough & tumble play, contribute longitudinally to children's ASC skills. | *Rough & Tumble*  Observed and coded rough and tumble play in childcare centre. | *Mental Health & Well-being Outcome*  a) observed expression of positive emotion  b) observed expression of negative emotion  c) emotion regulation, based on mother report on Preschool Characteristics Questionnaire. | T1 (cross-sectional)  r= -0.14, n.s.  r= 0.10, n.s.  r= 0.16, n.s.    T2 (follow-up outcomes 1 year later)  r= -0.22, p < .05  r= 0.24, p < .05  r= 0.16, n.s. | Minority negative effect on health |
| Lindsey (2014) (31) | Observational study to examine associations between preschool-aged children’s (n=148) physical activity play, including rough & tumble play, and peer competence in Texas, United States. | *Rough & Tumble*  Proportion of play time engaged in rough-and-tumble (R&T) play (observed during freeplay sessions at childcare centres):  1. Mixed gender chase  2. Mixed gender fighting  3. Same gender chase  4. Same gender fighting  5. Other gender chasing | *Social Outcome*  Preschooler’s peer acceptance (sociometric assessment) | GIRLS  1. r = .11, n.s.  2. r = 0.06, n.s.  3. r =.15, n.s.  4. r =: -0.18, n.s.  5. r = -.09, n.s.   BOYS  1. r = .32, p < .001  2. r = -0.20, n.s.  3. r = .32, p < .001  4. r = .31, p < .001  5. r = -.18, n.s. | Minority positive effect on health |
| Marques et al. (2014) (32) | Cross-sectional study to explore whether independent mobility is associated with physical activity levels in children aged 11-12 years (n=636) in Porto, Portugal. | *Independent Mobility*  Independent mobility, assessed through child self-report surveys | *Physical Outcome*  Accelerometers (30 s epoch):   MVPA (>2296 cpm; average daily minutes) | The odds of having higher MVPA when children have higher independent mobility increased through the quartiles (except for quartile 2, due to lack of significance).   Individual parameter estimates revealed that the odds of children with higher independent mobility to belong to quartile 3 compared with quartile 1 were 1.454 times more than children with lower independent mobility. The odds for belong to quartile 4 compared with quartile 1 were 1.458 times higher. | Consistently positive effect on health |
| McCormack et al. (2011) (33) | Cross-sectional study examining associations between socio-demographic characteristics, sedentary and active leisure-time behavior, independent mobility, active transportation and the built environment, and achievement of sex-specific pedometer-based cut-points, among elementary school-aged children aged 10-12 years (n=927) in Perth, Australia. | *Independent Mobility*  Independent mobility, reported by parents | *Physical Outcome*  Pedometers   Average daily steps were categorised into sex-specific pedometer-based cut-points (<15000 vs. ≥15000steps/day for boys and <12000 vs. ≥12000 steps/day for girls). Children were categorised based on whether or not they achieved these pedometer-based cut-points | Achieving pedometer-based cut-points – independent mobility   (OR 95% CI)  Main effects:  Yes: reference category  No: 0.71 (0.41, 1.14), p>0.05  Main effects and interactions:  Yes: Reference category  No: 0.73 (0.45, 1.18) p>0.05 | No consistent evidence of effect on health |
| Nelson et al. (2008) (34) | Observational study in the United States to explore whether children's aged 4-5 years (n=357) solitary-pretend play and solitary-functional play are related to one another and to other indices of social adjustment/maladjustment to explore for potential gender differences in these associations. | *Rough & Tumble*  1. Behavioural observation scans and coding of cooperative rough-and-tumble play.  2. Teacher rating of cooperative rough-and-tumble play | *Aggression Outcome*  a. Proactive aggression bullying (observed)  b. Proactive aggression bullying (teacher rated)  c. Proactive instrumental aggression (observed)  d. Proactive instrumental aggression (teacher rated)  e. Reactive aggression bullying (observed)  f. Reactive instrumental aggression (observed  g. Reactive physical aggression (teacher rated | 1a. r=0.32 p<.01  1b. r=0.12, p<.05  1c. r=0.06, n.s.  1d. r=0.11, n.s.  1e. r=0.10, n.s.  1f. r= 0.03, n.s.  1g. r= 0.12, p<0.05   2a. r=0.04, n.s.  2b. r=0.22, p<0.05  2c. r=0.09, n.s.  2d. r=-0.1, p<.01  2e. r=-0.08, n.s.  2f. r= 0.05, n.s.  2g. r=-0.06, n.s. | Minority negative effect on health |
|  |  |  | *Cognitive Outcome*  Teacher behavior rating scale (TBRS) – distractable subscale | 1. r = -0.05, *p* > .05  2. r = -0.11, p > .05 | No evidence of effect on health |
|  |  |  | *Social Outcome*  a. Victimisation (observed)  b. Active exclusion (observed)  c. Peer acceptance (peer rated)  d. Assertive (teacher rated)  e. Sociable/friendly behaviour (teacher rated) | 1a: r= 0.07, n.s.  1b. r= 0.04, n.s.  1c. r= -0.02, n.s.  1d. r= 0.20, p<.01  1e. r= 0.19, p<0.01   2a: r= 0.03, n.s.  2b. r= 0.04, n.s.  2c. r= -0.13, p<.05  2d. r= -0.13, p<.05  2e. r= 0.69, p< .01 | Minority positive effect on health |
| Nielsen et al. (2010) (35) | Cross-sectional study to investigate whether the number of permanent playground facilities in schools and total playground area influences objectively measured daily school and total physical activity in children aged 5 to 12 years (n=417) in Otago, New Zealand. | *Risky Play Supportive Environments*  Assessed number of permanent play facilities in school playgrounds | *Physical Outcome*  Accelerometers (15 s epoch)   a. unadjusted average activity counts   b. home time activity   i. average counts   ii. time in MPA (1500-6499 cpm)   iii. time in VPA (>6500 cpm)   iv. time in MVPA (>1500 cpm)   c. school time activity   i. average counts   ii. time in MPA (1500-6499 cpm)   iii. time in VPA (>6500 cpm)   iv. time in MVPA (>1500 cpm)    d. total activity   i. average counts   ii. time in MPA (1500-6499 cpm)   iii. time in VPA (>6500 cpm)   iv. time in MVPA (>1500 cpm) | a. r= 0.34, p<0.001   Ratios per unit increase in play facilities (95% CI)    bi. 1.026 (1.010, 1.042), p= 0.001  bii. 1.010 (0.997, 1.024), p= 0.140  biii. 1.049 (1.029, 1.071), p< 0.001  biv. 1.013 (0.999, 1.027), p= 0.078   ci. 1.038 (1.025, 1.051), p<0.001  cii. 1.019 (1.007, 1.031), p= 0.003  ciii. 1.101 (1.072, 1.132), p<0.001  civ. 1.023 (1.010, 1.037), p= 0.001   di. 1.027 (1.012, 1.041), p<0.001  dii. 1.031 (1.013 1.050), p= 0.001  diii. 1.102 (1.066, 1.139), p<0.001  div. 1.034 (1.015, 1.054), p<0.001 | Some positive effect on health |
| Noonan et al. (2016) (36) | Cross-sectional study to investigate differences in health-related, home and neighbourhood environmental variables between children aged 9-10 years (n=194) living in areas of high deprivation and medium-to-high deprivationand and assess associations between these perceived home and neighbourhood environments and health-related variables stratified by deprivation group in Liverpool, England. | *Independent Mobility*  Independent mobility, reported by parents | *Physical Outcome*  a. physical activity, assessed with the PAQ-C  b. cardiorespiratory fitness, assessed using the 20mSRT  c. BMI z-score, calculated from height and weight  d. waist circumference | β (95% CI)   a. HD: 0.03 (-0.03 to 0.08), p>0.05  MD: 0.07 (0.01 to 0.12), p<0.01  b. HD: p > .05, stats NR  MD: p > .05, stats NR  c. HD: p > .05, stats NR  MD: p > .05, stats NR  d. HD: p > .05, stats NR  MD: p > .05, stats NR | Minority positive effect on health |
| Page et al. (2009) (37) | Cross-sectional study to investigate whether self-reported independent mobility (IM) was related to objectively measured weekday and weekend physical activity in children aged 10-11 years (n=1,307) in Bristol, United Kingdom. | *Independent Mobility*  Independent mobility, assessed through computerised child self-report surveys. Categorised into:   1. local-IM  2. area-IM  3. local-IM*area-IM | *Physical Outcome*  Accelerometers (10 s epoch):    a. weekday PA (cpm)  b. weekend PA (cpm) | β (95% CI)   1a. Boys: 35.92 (21.59, 50.24), p=0.001  Girls: 16.88 (4.98, 28.79), p=0.005  1b. Boys: 25.77 (-1.63, 53.17), p= 0.065  Girls: 23.45 (3.42, 43.47), p=0.022   2a. Boys: 32.61 (18.80, 46.42), p<0.001  Girls: 21.26 (8.52, 33.99), p=0.001  2b. Boys: 25.42 (-1.31, 52.15), p=0.062  Girls: 18.93 (-2.80, 40.65), p=0.088    3a. Boys: 6.51 (-21.66, 15.01), p=0.547  Girls: 3.31 (-21.66, 15.04), p=0.724  3b. Boys: 8.40 (-32.07, 48.88), p=0.684  Girls: 3.25 (-28.87, 35.37), p=0.843 | Minority positive |
| Page et al. (2010) (38) | Cross-sectional study to investigate whether children aged 10-11 years (n=1,307) independent mobility and perceptions of the environment were related to frequency of participation in three different physical activity contexts: outdoor play, structured sport/exercise and mode of travel to school in Bristol, United Kingdom. | *Independent Mobility*  Independent mobility, assessed through computerised child self-report surveys. Categorised into:   1. local-IM  2. area-IM | *Physical Outcome*  Child self-reported questionnaire   a. frequency of structured exercise/sport  b. walking/cycling home from school | OR (95% CI)   1a. Boys: 1.42 (1.06, 1.89), p=0.017  Girls: 1.22 (0.95-1.58), p=0.122  1b. Boys: 1.41 (1.07-1.87), p=0.014  Girls: 1.49 (1.07-2.07), p=0.018    2a. Boys: 1.34 (0.98-1.82), p=0.065  Girls: 1.08 (0.82-1.44), p= 0.570  2b. Boys: OR= 1.03 (0.75-1.42), p=0.833  Girls: 0.91 (0.64-1.31), p=0.617 | Minority positive effect on health |
| Paquette et al. (2003) (39) | Three studies, cross-sectional and longitudinal in nature, are summarized to analyze the functions of father-child play fighting in the social adaptation of children, and to determine the prevalence and correlates of parent-child RTP (n=468/75/372) in Quebec, Canada. | *Rough & Tumble*  Study 1  1. Father-child rough and tumble play (retrospective child report)   Study 2  2. father-child rough and tumble play frequency | *Aggression Outcome*  Study 1  a. Physical aggression (teacher report)    Study 2  b. Physical aggression (father report) | Study 1  1a. rho=.15, p<.01    Study 2  2b. rho=.10, n.s. | Some negative effect on health |
| Pellegrini (1988) (40) | Cross-sectional study to examine the extent to which rough-and-tumble play moved into aggressive behavior and the relationship with social competence for popular and rejected children ages 5-9 years (n=94) in the United States. | *Rough & Tumble*  Observed rough and tumble play during recess | *Aggression Outcome*  Antisocial behaviour questionnaire (teacher report) | Popular children:  r = -.3, n.s.   Rejected children:  r = .47, p < .06 (note the exact p-value is not reported. Unclear whether < .05). | Some negative effect on health |
|  |  |  | *Social Outcome*  Social problem solving flexibility, assessed using the Interpersonal Cognitive Problem Solving (ICPS) | Popular children:  r = 0.56, p<0.01  Rejected children:  r = -0.21, n.s. | Some positive effect on health |
| Pellegrini (1989) (41) | Observational study to examine the differential effects of age, sex, and physical contexts on children's rough and tumble (R&T) play and aggression, the extent of how R&T play moved into aggressive behavior, and the functional significance, or beneficial consequences of R&T play among children ages 5-9 years (n=94) in the United States. | *Rough & Tumble*  Rough and tumble play coded from recorded audio notes made by observer watching children’s play during recess at school:  1. frequency  2. percentage of play | *Aggression Outcome*  Antisocial Behaviour, assessed via teacher report using The Children's Behavior Questionnaire | 1. boys: r = -0.04, n.s.; girls: r = 0.10, n.s.  2. boys r = .02, n.s.; girls r =.36, p < .01. | Minority negative effect on health |
|  |  |  | *Social Outcome*  Sociometric status via peer report:  a) social preference  b) likes most  c) likes least  d) Social problem solving, assessed using the Interpersonal Cognitive Problem Solving procedure. | 1a. boys r = .13, n.s.; girls r = .10, n.s.  1b. boys r = .22, n.s.; girls r = -.20, n.s.  1c. boys r = -.0002, n.s.; girls r = .08, n.s.  1d. boys r = .30, p < .05; girls r =-.03, n.s.   2a. boys r = -.01, n.s.; girls r = -.01, n.s.  2b. boys r = .18, n.s.; girls r = -.16, n.s.  2c. boys r = -.13, n.s.; girls r = .14, n.s.  2d. boys r = .31, p < .05; girls r = .01, n.s. | Minority positive effect on health |
| Pellegrini (1991) (42) | Examined longitudinal associations between rough and tumble play of popular and rejected children ages 5-9 years (n=94) with measures of social affiliation and social cognition in the United States. | *Rough & Tumble*  Percentage of observed behaviour coded as rough and tumble play during recess | *Social Outcome*  Social  problem solving as assessed using the Interpersonal Cognitive Problem Solving (ICPS) procedure | Rejected children:  R2 = .58, F = .01, p = .9  Popular children:  R2 = .28, F = 3.02, p = .09 | No consistent evidence of effect on health |
| Pellegrini (1993) (43) | Cross-sectional study to examine the extent to which aspects of R&T play are related to children aged 10-11 years (n=42) social problem solving and social affiliation after the variance due to games-with-rules and other forms of reciprocal social interaction has been statistically removed in the United States. | *Rough & Tumble*  Rough and tumble play observed during recess on school playground:  1. frequency (as a proportion of coded behaviour)  2. flexibility of rough and tumble behaviours  3. vigour (low to high) | *Social Outcome*  Temperament. Assessed using the Dimensions of Temperament Survey, teacher report:  a) popularity (sociometric assessment)   Social problem solving flexibility, assessed using an adaptation of the Interpersonal Cognitive Problem Solving (ICPS) procedure:  b) positive solutions  c) negative solutions | 1a) r = -0.22, n.s.  1b) r = -0.13, n.s.  1c) r =0.18, n.s.   2a) r = 0.16, n.s.  2b) r= 0.34 (p<0.05)  2c) r = 0.54 (p<0.01)   3a) r = 0.04, n.s.  3b) r = -0.03, n.s.  3c) r = 0.18, n.s. | No consistent evidence of effect on health |
| Pellegrini (1994) (44) | Cross-sectional study to to determine the relation between R&T/Chase and R&T/Rough and aggression, and address the possible benefits that may be associated with R&T play including social problem-solving flexibility and perspective-taking among children aged 10-13 years (n=54) in Georgia, United States. | *Rough & Tumble*  Behavioural observation of rough and tumble play during recess. Coded  1. rough and tumble, chase  2. rough and tumble, rough | *Aggression Outcome*  Aggression coded via behavioural observation | Correlation analyses by sociometric status.   1. Average: r= 0.28, NS; Popular: r= 0.00, NS; Rejected: r= -0.18, NS    2. Average: r= 0.21, NS; Popular: r= 0.00, NS; Rejected: r= 0.54, p<0.05 | Minority negative effect on health |
|  |  |  | *Social Outcome*  Social problem solving, assessed using the Interpersonal Cognitive Problem Solving procedure:  a. prosocial problem solving  b. antisocial problem solving  c. chandler’s measure of perspective taking | 1a. Popular: r= 0.18, NS; Rejected: r= 0.31, NS; Average: r= 0.40, p<0.10   1b. Popular: r= -0.17, NS; Rejected: r=-0.16, NS; Average: r= 0.05, NS   1c. Popular: r= 0.01, NS; Rejected: r= 0.33, NS; Average: r= 0.15, NS   2a. Popular: r= 0.11, NS; Rejected: r= -0.10, NS; Average: r= -0.08, NS   2b. Popular: r= -0.09, NS; Rejected: r= -0.19, NS; Average: r= 0.01, NS   2c. Popular: r= 0.03, NS; Rejected: r= 0.47, p<0.10;  Average: r=0.12, NS | No consistent evidence of effect on health |
| Pellegrini (1995) (45) | Longitudinal study to determine the relation of R&T to peer dominance during early adolescence at aged 12-13 years (n=82) in the United States. | *Rough & Tumble*  Behavioural observation of rough and tumble play during recess. Coded  1. rough and tumble, chase  2. rough and tumble, rough | *Aggression Outcome*  a. Aggression coded via behavioural observation  b. Aggressive factor from Teacher Checklist (teacher report) | Cross-sectional year 1  1a. r= 0.06, NS  1b. r= 0.03, NS  2a. r= 0.29, p<0.01  2b. r=0.25, p< .01    Cross-sectional year 2  1a. r= 0.07, NS  1b. r= 0.05, NS  2a. r= 0.12, NS  2b. r=0.19, NS    Longitudinal year 2 outcomes  1a. r= 0.12, NS  1b. r= 0.05, NS  2a. r= -.12, NS  2b. r=0.25, p < .05 | Minority negative effect on health |
|  |  |  | *Social Outcome*  Sociometric status via peer report:  a) likes most  b) likes least  c) social preference  d) victim status | Cross-sectional year 1  1a. r=-0.07, NS;  1b: r= -0.22, p<0.05;  1c: r= 0.04, NS;  1d: r= 0.22, p<0.05  2a: r=-0.37, p<0.01;  2b r=-0.06, NS;  2c: r=-0.13, NS;  2d: r=0.21, p<0.05    Cross-sectional year 2  1a: r=0.02, NS;  1b: r=0.11, NS;  1c: r=-0.04, NS;  1d: r=0.12, NS  2a: r=0.02, NS;  2b: r=0.14, NS;  2c: r= -0.16, NS;  2d: r=0.02, NS    Longitudinal year 2 outcomes  1a: r=0.12, NS;  1b: r=-0.19, NS;  1c: r=0.28, P<.01;  1d: r=0.15, NS   2a: r=0.01, NS;  2b: r=0.02, NS;  2c: r= -0.02, NS;  2d: r=0.13, NS | Minority negative effect on health |
| Pellegrini (2003) (46) | Cross-sectional study to explore how rough and tumble play during adolescence (n=138) is used to serve dominance ends and as a form of early heterosexual contact in the United States. | *Rough & Tumble*  Observations of rough and tumble play at school. | *Aggression Outcome*  Observations of aggression. | r=.31, p<0.01 | Consistently negative effect on health |
| Pepler et al. (1998) (47) | Cross-sectional study examining the mechanisms hypothesized to maintain children aged 6-12 years (n=39)aggressive behaviour patterns on the school playground that reside within the individual aggressive child and in their reciprocal interactions with peers in Canada. | *Rough & Tumble*  Rough and tumble observed on playgrounds during lunch and recess: 1. prosocial  2. antisocial | *Aggression Outcome*  Total observed antisocial behaviours during lunch and recess (social interaction, talk, gossip, reject, verbal attack, touch, attack) | r = -.12, n.s.  r = .26, n.s. | No clear evidence effect on health |
|  |  |  | *Social Outcome*  Total observed prosocial behaviours during lunch and recess (social, talk, gossip, touch) | 1. r = .01, n.s.  2. r = -.04, n.s. | No consistent effect on health |
| Sando et al. (2021) (48) | Cross-sectional study to investigate the relationship between children aged 3-5 years (n=79) risky play, well-being, involvement, and physical activity in Norway. | *General Risky Play*  Risky play coded from video observations of free play. | *Mental Health & Well-being Outcome*  Observation of:  a. involvement  b. well-being | a. r = 0.27, p < .001  b. r = 0.19, p < .001 | Consistently positive effect on health |
| Schoeppe et al. (2014) (49) | Cross-sectional study to investigate associations between children aged 8-13 years (n=191) self-reported independent mobility (including independent travel and outdoor play) and objectively measured light, moderate-to-vigorous, and total physical activity in multiple cities across Australia. | *Independent Mobility*  Independent mobility, assessed through child self-report surveys. Categorised into: | *Physical Outcome*  Combined HR and accelerometer monitor (60 s epoch):   a. LPA (101-2295 cpm)  b. MVPA: (³2296 cpm)  c. total PA | β (95% CI)  1a. -0.55 (-19.12-18.02), p= 0.95  1b. -1.48 (-12.86-9.90), p=0.80  1c. -2.53 (-25.92-20.85), p= 0.83   2a. 29.73 (9.39-50.06), p<0.01  2b. 3.96 (-12.01-19.93), p=0.63  2c. 32.43 (3.23-61.62), p=0.03   3a. -13.24 (-35.85-9.37), p=0.25  3b. 4.81 (-11.17-20.78), p=0.56  3c. -4.10 (-39.96-31.76), p=0.82 | Minority positive effect on health |
| Stone et al. (2014) (50) | Cross-sectional study to examine if children’s aged 10-12 years (n=856) in Toronto, Canada independent mobility is related to multiple characteristics of accelerometry-measured PA behaviour (total PA and time spent sedentary and in light and moderate-to-vigorous PA). | *Independent Mobility*  Independent mobility, reported by parents | *Physical Outcome*  Accelerometers (5 s epoch):   a. weekday PA   i. total PA (counts.day-1)   ii. LPA (min)   iii. MVPA (min)   iv. Time spent sedentary (%)   b. weekend PA   i. total PA (counts.day-1)   ii. LPA (min)   iii. MVPA (min)   iv. Time spent sedentary (%)   c. After school PA   i. % time sedentary   ii. % time in LPA   iii. % time in MVPA | High IM vs. Low IM  ai. Boys: F388= 6.2, p= 0.013  Girls: F467 =7.3, p= 0.01  aii. Boys: p > .05, stats NR  Girls: p >0.05, stats NR  aiii. Boys: F388 = 6.2, p=0.013  Girls: F467 =5.8, p=0.01  aiv. Boys: p >0.05, stats NR  Girls: F467 = 10.5, p=0.001   bi. Boys: F388 =3.7, p=0.040  Girls: p<0.05, stats NR  bii. Boys: p>0.05, stats NR  Girls: p<0.05, stats NR  biii. Boys: F388 = 3.4, p=0.04  Girls: p<0.05, stats NR  biv. Boys: p>0.05, stats NR  Girls: p<0.05, stats NR   ci. Boys: p<0.05, stats NR  Girls: p<0.05, stats NR  cii. Boys: p<0.05, stats NR  Girls: p<0.05, stats NR  ciii. Boys: p<0.05, stats NR  Girls p<0.05, stats NR | Some positive effect on health |
| Storli and Hagen (2010) (51) | Case-control study to explore children aged 3-5 years (n=16) active play outdoors in traditional playgrounds and natural environments in Norway. | *Risky Play Supportive Environments*  Compared playground type: traditional vs. nature play | *Physical Outcome*  Accelerometers (epoch NR)   Mean level of PA (cpm) in:  a. traditional playground, spring  b. traditional playground, winter  c. natural environment, spring | a. p>0.05, stats NR  b. p>0.05, stats NR  c. p>0.05, stats NR | No consistent evidence of effect on health |
| Storli and Lorås (2025) (52) | Cross-sectional study to explore the relationship between Norweigan children’s (aged 7-10) risk willingness and their motor behavior in a virtual playground setting and its association with perceived gross motor competence (n=96 children). | *General Risky Play*  Virtual reality environment assessing risky play experience based on child risk willingness (high versus low). | *Physical Outcome*  a. whole-body motion capture analyzed with scale of Perceived Movement Skill Competence  b. self-reported perceived gross motor competence | a. high-risk-willingness group moved a longer total distance during the three-minute period (t(94) = 5.76, p < 0.001, ΔM = 15.40 [95% CI: 10.09, 20.72], d = 1.34 [95% CI: 0.84, 1.84]) and with higher average velocity (t(94) = 5.92, p < 0.001, ΔM = 0.11 [95% CI: 0.07, 0.15], d = 1.32 [95% CI: 0.83, 1.79]) compared to the low-risk-willingness group. The mean acceleration was also higher in the children with higher risk willingness (t(94) = 6.36, p < 0.001, ΔM = 0.17 [95% CI = 0.11, 0.22], d = 1.48 [95% CI: 0.97, 1.99]).  b. Perceived locomotor skills (running item): t(94) = 2.67, p = 0.009, ΔM = 0.39 [95% CI = 0.10, 0.67], d = 0.61 [95% CI: 0.15, 1.06] - significantly different between groups, with higher ratings from the children in the high-risk-willingness group. | Minority positive effect on health |
| Veiga et al. (2022) (53) | Cross-sectional study to examine the relationship between rough and tumble play, emotion regulation skills and aggressive behavior among children aged 3-5 years (n=90) in Lisbon, Portugal. | *Rough & Tumble*  1. Peer-to-peer rough and tumble play observed at school during outdoor recess.  2. Frequency of rough-and-tumble play at home (mother and father report)  3. Duration of rough-and-tumble play (father report) | *Mental Health & Well-being Outcome*  Emotion Dysregulation assessed via parent-report subscale of the Emotion Expression Questionnaire (mother report) | 1. Boys: r= -0.27, NS; Girls: r= 0.25, NS  2. r=0.35, p<0.001  3. r= 0.42, p<0.001 | Some negative effect on health |
|  |  |  | *Aggression Outcome*  Aggressive Behaviours Questionnaire, completed by mothers | 1. RTP at school: r= 0.25, p<0.05  2. r=0.41, p<0.001  3. Boys: r=-0.46, n.s.; Girls: r= -0.32, p<0.01 | Minority negative effect on health |

## Table S2‑2: Studies contributing to qualitative analysis (n=25)

| **Lead author (year)** | **Study aim and design** | **Risky play exposure** | **Data collection** | **Participants** | **Data analysis** | **Reported outcomes** |
| --- | --- | --- | --- | --- | --- | --- |
| Bauer et al. (2024) (54) | Phenomenological design to explore “What are military mothers’ perspectives on their children’s (aged 4-12 years) outdoor risky play and how may these perspectives be shaped by their military experiences?” with Canadian mothers. | General risky play | Semi-structured Interviews | Mothers (n=16) | Reflexive TA | Social problem solving  Cognitive problem solving  Risk competence  Injuries (-)  Antifragility |
| Bauer and Pike (2023) (55) | Phenomenological design to explore ‘What are children’s perspectives on safe and dangerous outdoor play and unintentional injuries?’ using a child- centred community- based participatory research approach with Canadian children aged 9-13 years. | General risky play | Unstructured Interviews | Children aged 9-13 years (n=13) | Thematic analysis | Enjoyment  Risk competence  Injuries (-) |
| Branje et al. (2021) (4) | Mixed-method, clustered, randomized control trial (RCT) to investigate the impact of a childcare-based outdoor loose parts play intervention on preschoolers' aged 3-5 years (n=197) fundamental movement skills in Nova Scotia, Canada.  Educators participated in focus groups exploring their perceptions of the intervention for children. | Risky play environments (loose parts play in ECEC settings) | Focus groups  Photo-elicitation | Educators (n=24 during the intervention and 27 post-intervention) | Thematic analysis | MVPA  Motor competence  Enjoyment  Self-efficacy  Self-regulation  Cooperation  Communication skills  Creativity  Perceptual awareness  Cognitive problem solving  Language development  Risk competence  Antifragility |
| Brussoni et al. (2017) (5) | Convergent mixed methods repeated measures study to investigate the effects of natural risky play environments on children's aged 2-5 years health and well-being in two childcare centres in Vancouver, Canada. Educators participated in 2 focus groups to explore their perceptions of the intervention. | Risky play environments (playground renovation with natural features) | Focus Groups, Field notes,  Observation,  Spatial behaviour maps | Educators (n=16),  Children aged 2-5 years (n=45) | Data coded, categorised and organised into themes | Self-confidence  Self-regulation  Social competence  Creativity  Cognitive problem solving  Injuries (-) |
| Bundy et al. (2009)(6) | Non-randomised experimental study to investigate a primary school intervention to promote active, social, and creative play through the addition of loose parts for children aged 5-7 years to play with within their school ground in Western Sydney, Australia. 9 teachers participated in interviews investigating their perceptions of the play materials on the playground and their impact on play. | Risky play environments (loose parts play in school playground) | Semi-structured Interviews (individual or paired) | Teachers (n=9) | Constant comparative analysis | MVPA  Cardiovascular fitness  Strength  Enjoyment  Resilience  Negative social behaviours (-)  Social competence  Cooperation  Communication skills  Creativity  Risk competence  Injuries (-)  Antifragility |
| Bundy et al. (2017)(7) | Randomised controlled trial in Sydney, Australia that aimed to promote play of 5 to 7-yeard-old children (n=113) through an intervention that placed recycled materials with no obvious play value on school playgrounds. Process evaluation during the intervention aimed to enrich understanding of play that was occurring. | Risky play environments (loose parts play in school playground) | Interviews,  Observations (video and photography),  Field notes | Teachers,  Principals  (number not specified) | Data categorised and organised into themes | Cardiovascular fitness  Negative social behaviours (-)  Social competence  Creativity  Language development  Risk competence  Injuries (-) |
| Coe (2017) (56) | Phenomenological design to explore the safe risk-taking and risky play experiences of four children (aged 4-5 years) at a nature-based early years programme in rural Ontario, Canada. | Risky play environments (nature-based ECEC program) | Observation, Photographs,  Interviews (students and teacher),  Photo elicitation (students) | Children aged 4-5 years (n=4), Teacher (n=1) | Data coded, categorised and organised into themes | MVPA  Cardiovascular fitness  Strength  Enjoyment  Self-efficacy  Cooperation  Perceptual awareness  Risk competence |
| Farmer et al. (2017) (57) | Randomised controlled trial to determine whether providing greater opportunities for risk and challenge in primary schools in Otago & Auckland regions of New Zealand increased children aged 6-9 years (n=840) physical activity and reduced relative body weight over the long term. School leaders participated in interviews examining their perceptions of the intervention. | Risky play environments (loose parts play in school playground) | Semi-structured Interviews (Individual or paired),  Field Notes,  Reflective research diary | Primary school principals and administrators (n=10) | Abductive analysis | MVPA  Motor competence  Cardiovascular fitness  Self-confidence  Negative social behaviours (-)  Cooperation  Creativity  Risk competence |
| Flannigan and Dietze (2018) (58) | Qualitative descriptive design to examine 4–5-year-old children’s play themes, patterns, and behaviours during the use of familiar loose parts in a natural outdoor environment in Canadian early childhood education and care. | Risky play environments (loose parts play in ECEC setting) | Observation, Videos,  Field notes | Children aged 4-5 years (n=27),  Educators (n=4) | Thematic analysis | MVPA  Self-confidence  Self-regulation  Negative social behaviours (+)  Social competence  Cooperation  Communication skills  Social problem solving  Creativity  Cognitive problem solving  Language development  Risk competence |
| Higginbottom et al. (2023) (59) | Qualitative descriptive design to explore (1) How do risk-focused interventions impact on children’ s risk-competence? and (2) How does intentional teaching promote children’s management of risk? As part of an action research project with early childhood educators in Australia to foster intentional teaching to promote risky play. | Risky play environments  (ECEC settings) | Pre-intervention Questionnaires  Pre-intervention and post-intervention: Interviews  Post-intervention:  Video, Photos | Children aged 2-6 years (n=60), Educators (n=15) | Abductive reasoning, data coded, themes generated | Risk competence  Injuries (-) |
| Hinchion et al. (2021) (60) | Ethnographic design to explore children’s subjective experiences and the meaning of risky play in their everyday contexts and environments in Ireland. | General risky play | Focus groups,  Photography tours,  Map-making, Observations | Children aged 6 - 8 years (n=10) | Thematic analysis | MVPA  Motor competence  Enjoyment  Self-efficacy  Social competence  Cooperation  Creativity  Risk competence |
| Little (2015)(61) | Phenomenological design to explore mothers’ beliefs about factors influencing risk-taking in play (of children aged 4-5 years) in Australia. | General risky play | Semi-structured Interviews | Mothers (n=26) | Constant comparisons | MVPA  Motor competence  Cardiovascular fitness  Strength  Enjoyment  Self-confidence  Self-regulation  Resilience  Cognitive problem solving  Risk competence  Injuries (-)  Antifragility |
| McCormack et al. (2024)(62) | Phenomenological design to explore knowledge, attitudes and perspectives regarding play among playworkers (“play ambassadors”) and caregivers exposed to a community based unstructured play intervention for children aged 3-11 years implemented in Calgary, Canada. | Risky play environments (loose parts play in local parks) | Semi-structured Interviews | Caregivers (n=9 parents, n=1 grandparent),  Play ambassadors (n=4) | Thematic analysis | Motor competence  Self-confidence  Self-regulation  Creativity  Risk competence  Injuries (+ risk of)  Antifragility |
| Oliver et al. (2022) (63) | Qualitative descriptive design to analyse data from a subsample of parents in Britain who participated in the nationally representative British Children’s Play Survey. Parents of children aged 5-11 years responded to two open-ended questions pertaining to the barriers to and facilitators of children’s adventurous play. | General risky play | Survey (open-ended) | Parents (n = 377) | Framework analysis | MVPA  Enjoyment  Self-confidence  Resilience  Cognitive problem solving  Risk competence  Injuries (+ risk of)  Antifragility |
| Rutberg et al. (2025)(64) | Qualitative descriptive photovoice design to explore children’s experiences of active school travel to inform a policy recommendation in Sweden. | Independent mobility (active school travel) | Photo-voice,  Focus groups | Children aged approximately 9-10 years (fourth graders) (n=43) | Qualitative content analysis | MVPA  Cardiovascular fitness  Enjoyment  Social competence |
| Sandseter (2009) (65) | Ethnographic design to explore children’s expressions of how they experience different kinds of risky play  In early childhood education and care settings in Norway. | General risky play (ECEC setting) | Observation (video) | Children aged 4-5 Years (n=29) | Data categorised, coded and interpreted | MVPA  Enjoyment  Self-efficacy  Negative social behaviours (+ , -)  Risk competence |
| Sandseter (2012) (66) | Phenomenological design investigating early childhood education and care practitioners’ opinions and reflections on children’s (3-5 years) risky play in Norwegian kindergartens. | General risky play | Semi-structured Interviews | ECEC practitioners (n=7)  Children aged 3-5 years (n=16) | Thematic Content analysis | MVPA  Enjoyment  Self-confidence  Self-efficacy  Injuries (-)  Antifragility |
| Speldewinde (2025)(67) | Ethnographic design to investigate how science teaching and learning occurs in nature-based early childhood education and care contexts such as ‘bush kinders’, and children’s risk and resilience in nature-based learning. | Risky play environments (bush kinder) | Ethnographic observation,  Field notes,  Journaling,  Photographs | Children aged 4-5 years (n=220)  Educators (n=15) | Thematic analysis | MVPA  Motor competence  Self-efficacy  Experiential learning  Risk competence |
| Spencer et al. (2021)(68) | Qualitative descriptive design to explore early childhood educators’ perspectives of risky play, in the context of the Physical Literacy in the Early Years (PLEY) loose parts play intervention in Canadia early childcare settings (for children aged 3-5 years). | Risky play environments (loose parts play in ECEC setting) | Focus groups | Educators (n=not specified; 15 focus groups with 3-5 participants) | Thematic analysis | MVPA  Motor competence  Strength  Enjoyment  Self-confidence  Cognitive problem solving  Risk competence  Injuries (-)  Antifragility |
| Sterman et al. (2020) (69) | Qualitative descriptive design to learn from participants about the utility of a loose parts play intervention for promoting choice and control among children with disability on the primary school playground in Australia.  Child age not specified; primary school age range is 5-17 years. | Risky play environments (loose parts play in school playground) | Semi-structured Interviews | School staff (teaching assistants, teachers, therapists, school leadership) (n=27) | Data coded, categorised and organised into themes | Social competence  Cooperation  Creativity  Risk competence |
| StGeorge (2018) (70) | Phenomenological design to investigate parents’ beliefs and knowledge about father–child rough and tumble play in Australia (child mean age 8.4 years). | Rough and tumble play | Semi-structured Interviews  Video-elicitation | Parents (n=52) | Thematic analysis | Motor competence  Enjoyment  Self-confidence  Self-regulation  Resilience  Negative social behaviours (+, -)  Social competence  Perceptual awareness  Injuries (-)  Antifragility |
| Traub (2025) (71) | Qualitative study in the USA to understand how directors, educators, and parents conceptualize risk within nature preschools using interviews. | Risky play environments (nature preschool) | Semi-structured interviews | ECE Directors (n=5); Teachers (n=4); Parents (n=4) | Emergent coding/analysis | Physical activity  Motor competence  Self-confidence  Anxiety  Experiential learning  Risk assessment  Injury occurrence (+ & -) |
| van Rooijen et al. (2023) (72) | Phenomenological design to explore children’s notions of risk and challenge in play during a loose parts intervention stimulating risky play and facilitated by after-school childcare practitioners in the Netherlands. | Risky play environments (loose parts play in after school program) | Observation, Informal interviews, Semi-structured round table talks,  Field notes | Afterschool childcare observation (n=475; children aged 4-10 years)  Roundtable talks (n=49; children aged 4-10 years) | Thematic analysis | Motor competence  Enjoyment  Self-confidence  Self-efficacy  Negative social behaviours (+) Social competence  Communication skills  Social problem solving  Creativity  Risk competence  Antifragility |
| Weir (2023) (73) | Phenomenological design to  Explore 9–10-year-old children’s experiences of their neighbourhoods in east London, England in order to contribute to understandings of how children experience and use their neighbourhoods. | Independent mobility | Go-along interviews, Annotated mapping, Workshops, Travel diaries | Children aged 9-10 years (n=17) | Thematic analysis | MVPA  Social competence  Risk competence  Antifragility |
| Zamani (2016) (74) | Qualitative descriptive design to explore young children’s cognitive play opportunities in natural, manufactured and mixed outdoor preschool zones in Clemson, USA (as part of a mixed method cross-sectional study). | Risky play environments (outdoor preschool site that had diverse natural and manufactured settings) | Semi-structured Interviews, photographic preferences and drawing session  (children)  Semi-structured interviews (Educators) | Children aged 4-5 years (n=22)  Educators (n=4) | Data coded, categorised and organised into themes | MVPA  Motor competence  Enjoyment  Self-confidence  Resilience  Cooperation  Creativity  Perceptual awareness  Experiential learning  Antifragility |

**References**

1.	Adams J, Veitch J, Barnett L. Physical Activity and Fundamental Motor Skill Performance of 5–10 Year Old Children in Three Different Playgrounds. Int J Environ Res Public Health. 2018 Aug 31;15(9):1896. doi:10.3390/ijerph15091896

2.	Aggio D, Gardner B, Roberts J, Johnstone J, Stubbs B, Williams G, et al. Correlates of children’s independent outdoor play: Cross-sectional analyses from the Millennium Cohort Study. Prev Med Rep. 2017;8(101643766):10–4. doi:10.1016/j.pmedr.2017.07.007

3.	Aliyas Z, Collins PA, Sylvestre MP, Frohlich KL. Investigating social inequalities in children’s independent mobility, active transportation and outdoor free play in two Canadian cities. Prev Med Rep. 2024;39(101643766):102642. doi:10.1016/j.pmedr.2024.102642

4.	Branje K, Stevens D, Hobson H, Kirk S, Stone M. Impact of an outdoor loose parts intervention on Nova Scotia preschoolers’ fundamental movement skills: a multi-methods randomized controlled trial. AIMS Public Health. 2022;9(1):194–215. doi:10.3934/publichealth.2022015

5.	Brussoni M, Ishikawa T, Brunelle S, Herrington S. Landscapes for play: Effects of an intervention to promote nature-based risky play in early childhood centres. J Environ Psychol. 2017 Dec 1;54:139–50. doi:10.1016/j.jenvp.2017.11.001

6.	Bundy AC, Luckett T, Tranter PJ, Naughton GA, Wyver SR, Ragen J, et al. The risk is that there is ‘no risk’: a simple, innovative intervention to increase children’s activity levels. Int J Early Years Educ. 2009;17(1):33–45.

7.	Bundy A, Engelen L, Wyver S, Tranter P, Ragen J, Bauman A, et al. Sydney Playground Project: A Cluster-Randomized Trial to Increase Physical Activity, Play, and Social Skills. J Sch Health. 2017;87(10):751–9. doi:10.1111/josh.12550

8.	Cho HJ, Jung S, Lee SE, Jo JH, Miller E. Young children’s self-control moderates the relationship between risky outdoor play and injury experiences in naturalistic settings. Early Child Dev Care. 2023;No-Specified. doi:10.1080/03004430.2023.2173187

9.	Colwell MJ, Lindsey EW. Preschool Children’s Pretend and Physical Play and Sex of Play Partner: Connections to Peer Competence. Sex Roles J Res. 2005;52(7–8):497–509. doi:10.1007/s11199-005-3716-8

10.	De Meester F, Van Dyck D, De Bourdeaudhuij I, Cardon G. Parental perceived neighborhood attributes: associations with active transport and physical activity among 10-12 year old children and the mediating role of independent mobility. BMC Public Health. 2014;14(1):631–631. doi:10.1186/1471-2458-14-631

11.	Dodd HF, Nesbit RJ, FitzGibbon L. Child’s play: Examining the association between time spent playing and child mental health. Child Psychiatry Hum Dev. 2022;54(6):1678–86. doi:10.1007/s10578-022-01363-2

12.	Engelen L, Bundy AC, Naughton G, Simpson JM, Bauman A, Ragen J, et al. Increasing physical activity in young primary school children — it’s child’s play: A cluster randomised controlled trial. Prev Med. 2013 May;56(5):319–25. doi:10.1016/j.ypmed.2013.02.007

13.	Farmer VL, Williams SM, Mann JI, Schofield G, McPhee JC, Taylor RW. Change of School Playground Environment on Bullying: A Randomized Controlled Trial. Pediatrics. 2017;139(5):1–10. doi:10.1542/peds.2016-3072

14.	Farmer VL, Williams SM, Mann JI, Schofield G, McPhee JC, Taylor RW. The effect of increasing risk and challenge in the school playground on physical activity and weight in children: a cluster randomised controlled trial (PLAY). Int J Obes 2005. 2017;41(5):793–800. doi:10.1038/ijo.2017.41

15.	Finegan JAK, Niccols GA, Zacher JE, Hood JE. The Play Activity Questionnaire: A parent report measure of children’s play preferences. Arch Sex Behav. 1991;20(4):393–408. doi:10.1007/BF01542619

16.	Flanders JL, Leo V, Paquette D, Pihl RO, Séguin JR, Flanders JL, et al. Rough-and-tumble play and the regulation of aggression: an observational study of father-child play dyads. Aggress Behav. 2009;35(4):285–95. doi:10.1002/ab.20309

17.	Flanders JL, Simard M, Paquette D, Parent S, Vitaro F, Pihl RO, et al. Rough-and-tumble play and the development of physical aggression and emotion regulation: a five-year follow-up study. J Fam Violence. 2010;25(4):357–67. doi:10.1007/s10896-009-9297-5

18.	Fliek L, Daemen E, Roelofs J, Muris P. Rough-and-Tumble Play and Other Parental Factors as Correlates of Anxiety Symptoms in Preschool Children. J Child Fam Stud. 2015;24(9):2795–804. doi:10.1007/s10826-014-0083-5

19.	Freeman EE, Robinson EL. The Relationship between Father–Child Rough-and-Tumble Play and Children’s Working Memory. Children. 2022;9(7):N.PAG-N.PAG. doi:10.3390/children9070962

20.	Garcia-Cervantes L, D’Haese S, Izquierdo-Gomez R, Padilla-Moledo C, Fernandez-Santos JR, Cardon G, et al. Physical Activity Coparticipation and Independent Mobility as Correlates of Objectively Measured Nonschool Physical Activity in Different School Grades: The UP&DOWN Study. J Phys Act Health. 2016;13(7):747–53.

21.	Hamamci B, Balaban Dagal A. Self-Regulation and Play: How Children’s Play Directed with Executive Function and Emotion Regulation. Early Child Dev Care. 2022;192(13):2149–59. doi:10.1080/03004430.2021.1990906

22.	Hart CH, DeWolf DM, Burts DC. Linkages Among Preschoolers’ Playground Behavior, Outcome Expectations, and Parental Disciplinary Strategies. Early Educ Dev. 1992 Oct;3(4):265–83. doi:10.1207/s15566935eed0304_1

23.	Huang WY, Feng J, Wong SH. Independent mobility and physical activity among children residing in an ultra-dense metropolis. Transl Exerc Biomed. 2024 Dec 19;1(3–4):236–43. doi:10.1515/teb-2024-0006

24.	Imai N, Shikano A, Kidokoro T, Noi S. Risky Play and Social Behaviors among Japanese Preschoolers: Direct Observation Method. Int J Environ Res Public Health. 2022;19(13):7889. doi:10.3390/ijerph19137889

25.	Kirby J, Levin KA, Inchley J. Parental and Peer Influences on Physical Activity Among Scottish Adolescents: A Longitudinal Study. J Phys Act Health. 2011 Aug 1;8(6):785–93. doi:10.1123/jpah.8.6.785

26.	Larouche R, Barnes JD, Blanchette S, Faulkner G, Riazi NA, Trudeau F, et al. Relationships Among Children’s Independent Mobility, Active Transportation, and Physical Activity: A Multisite Cross-Sectional Study. Pediatr Exerc Sci. 2020;32(4):189–96. doi:10.1123/pes.2019-0238

27.	Larouche R, Faulkner G, Bélanger M, Brussoni M, Gunnell K, Tremblay MS. “Out and about”: relationships between children’s independent mobility and mental health in a national longitudinal study. Child Geogr. 2024 Nov;22(6):860–70. doi:10.1080/14733285.2024.2397734

28.	Lavrysen A, Bertrands E, Leyssen L, Smets L, Vanderspikken A, De Graef P. Risky-Play at School. Facilitating Risk Perception and Competence in Young Children. Eur Early Child Educ Res J. 2017;25(1):89–105.

29.	Leung KYK, Loo BPY. Association of children’s mobility and wellbeing: A case study in Hong Kong. Travel Behav Soc. 2017;9:95–104. doi:10.1016/j.tbs.2017.07.004

30.	Lindsey EW, Colwell MJ. Pretend and physical play: Links to preschoolers’ affective social competence. Merrill-Palmer Q. 2013;59(3):330–60. doi:10.1353/mpq.2013.0015

31.	Lindsey EW. Physical activity play and preschool children’s peer acceptance: Distinctions between rough-and-tumble and exercise play. Early Educ Dev. 2014;25(3):277–94. doi:10.1080/10409289.2014.890854

32.	Marques EA, Pizarro AI, Mota J, Santos MP. Independent Mobility and its Relationship With Moderate-to-Vigorous Physical Activity in Middle-School Portuguese Boys and Girls. J Phys Act Health. 2014;11(8):1640–3.

33.	McCormack GR, Giles-Corti B, Timperio A, Wood G, Villanueva K. A cross-sectional study of the individual, social, and built environmental correlates of pedometer-based physical activity among elementary school children. Int J Behav Nutr Phys Act. 2011;8. doi:10.1186/1479-5868-8-30

34.	Nelson LJ, Hart CH, Evans CA. Solitary‐functional Play and Solitary‐pretend Play: Another Look at the Construct of Solitary‐active Behavior Using Playground Observations. Soc Dev. 2008 Nov;17(4):812–31. doi:10.1111/j.1467-9507.2008.00470.x

35.	Nielsen G, Taylor R, Williams S, Mann J. Permanent play facilities in school playgrounds as a determinant of children’s activity. J Phys Act Health. 2010;7(4):490–6.

36.	Noonan RJ, Boddy LM, Knowles ZR, Fairclough SJ. Cross-sectional associations between high-deprivation home and neighbourhood environments, and health-related variables among Liverpool children. BMJ Open. 2016;6(1):e008693. doi:10.1136/bmjopen-2015-008693

37.	Page AS, Cooper AR, Griew P, Davis L, Hillsdon M. Independent mobility in relation to weekday and weekend physical activity in children aged 10–11 years: The PEACH Project. Int J Behav Nutr Phys Act. 2009;6(1):2. doi:10.1186/1479-5868-6-2

38.	Page AS, Cooper AR, Griew P, Jago R. Independent mobility, perceptions of the built environment and children’s participation in play, active travel and structured exercise and sport: the PEACH Project. Int J Behav Nutr Phys Act. 2010;7(1):17. doi:10.1186/1479-5868-7-17

39.	Paquette D, Carbonneau R, Dubeau D, Bigras M, Tremblay RE. Prevalence of father-child rough-and-tumble play and physical aggression in preschool children. Eur J Psychol Educ. 2003;18(2):171–89. doi:10.1007/BF03173483

40.	Pellegrini AD. Elementary-school children’s rough-and-tumble play and social competence. Dev Psychol. 1988;24(6):802–6. doi:10.1037/0012-1649.24.6.802

41.	Pellegrini AD. Elementary school children’s rough-and-tumble play. Early Child Res Q. 1989 Jun;4(2):245–60. doi:10.1016/S0885-2006(89)80006-7

42.	Pellegrini AD. A Longitudinal Study of Popular and Rejected Children’s Rough-and-Tumble Play. Early Educ Dev. 1991;2(3):205–13.

43.	Pellegrini AD. Boys’ rough-and-tumble play, social competence and group composition. Br J Dev Psychol. 1993;11(3):237–48. doi:10.1111/j.2044-835X.1993.tb00600.x

44.	Pellegrini AD. The rough play of adolescent boys of differing sociometric status. Int J Behav Dev. 1994;17(3):525–40. doi:10.1177/016502549401700308

45.	Pellegrini AD. A longitudinal study of boys’ rough-and-tumble play and dominance during early adolescence. J Appl Dev Psychol. 1995;16(1):77–93. doi:10.1016/0193-3973%2895%2990017-9

46.	Pellegrini AD. Perceptions and Functions of Play and Real Fighting in Early Adolescence. Child Dev. 2003;74(5):1522–33. doi:10.1111/1467-8624.00620

47.	Pepler DJ, Craig WM, Roberts WL. Observations of aggressive and nonaggressive children on the school playground. Merrill-Palmer Q. 1998;44(1):55–76.

48.	Sando OJ, Kleppe R, Sandseter EBH. Risky Play and Children’s Well-Being, Involvement and Physical Activity. Child Indic Res. 2021 Aug;14(4):1435–51. doi:10.1007/s12187-021-09804-5

49.	Schoeppe S, Duncan MJ, Badland HM, Oliver M, Browne M. Associations between children’s independent mobility and physical activity. BMC Public Health. 2014 Dec;14(1):91. doi:10.1186/1471-2458-14-91

50.	Stone MR, Faulkner GE, Mitra R, Buliung RN. The freedom to explore: examining the influence of independent mobility on weekday, weekend and after-school physical activity behaviour in children living in urban and inner-suburban neighbourhoods of varying socioeconomic status. 2014.

51.	Storli R, Hagen TL. Affordances in outdoor environments and children’s physically active play in pre‐school. Eur Early Child Educ Res J. 2010 Dec;18(4):445–56. doi:10.1080/1350293X.2010.525923

52.	Storli L, Lorås H. Movement Variability and Perceived Motor Competence in Children with High or Low Risk Willingness in a Virtual Playground. Children. 2025 Jun 18;12(6):796. doi:10.3390/children12060796

53.	Veiga G, O’Connor R, Neto C, Rieffe C. Rough-and-tumble play and the regulation of aggression in preschoolers. Early Child Dev Care. 2022;192(6):980–92. doi:10.1080/03004430.2020.1828396

54.	Bauer MEE, Giles AR, Brussoni M. “I’ve seen what evil men do”: Military mothering and children’s outdoor risky play. Leis Sci. 2024;46(2):150–66. doi:10.1080/01490400.2021.1920521

55.	Bauer MEE, Pike I. What children’s perspectives on safe and dangerous outdoor play can tell us about their risk-seeking and injury experiences: ‘You don’t feel right doing the thing that got you hurt.’ Inj Prev. 2023 Oct;29(5):367–70. doi:10.1136/ip-2023-044865

56.	Coe HA. Embracing Risk in the Canadian Woodlands: Four Children’s Risky Play and Risk-Taking Experiences in a Canadian Forest Kindergarten. J Early Child Res. 2017;15(4):374–88.

57.	Farmer VL, Fitzgerald RP, Williams SM, Mann JI, Schofield G, McPhee JC, et al. What did schools experience from participating in a randomised controlled study (PLAY) that prioritised risk and challenge in active play for children while at school? J Adventure Educ Outdoor Learn. 2017;17(3):239–57. doi:10.1080/14729679.2017.1286993

58.	Flannigan C, Dietze B. Children, Outdoor Play, and Loose Parts. J Child Stud. 2018 Mar 17;42(4):53–60. doi:10.18357/jcs.v42i4.18103

59.	Higginbottom K, Newman L, West-Sooby K, Wood A. Intentional Teaching for Risky Play: Practitioner Researchers Move Beyond Their Comfort Zones. Australas J Early Child. 2023 Mar;48(1):18–33. doi:10.1177/18369391221112740

60.	Hinchion S, McAuliffe E, Lynch H. Fraught with Frights or Full of Fun: Perspectives of Risky Play among Six-to-Eight-Year Olds. Eur Early Child Educ Res J. 2021;29(5):696–714.

61.	Little H. Mothers’ beliefs about risk and risk-taking in children’s outdoor play. J Adventure Educ Outdoor Learn. 2015;15(1):24–39. doi:10.1080/14729679.2013.842178

62.	McCormack GR, Naish C, Petersen J, Ghoneim D, Doyle-Baker PK. It is child’s play: Caregiver and playworker perspectives on a community park-based unstructured play program. De Silva D, editor. PLOS ONE. 2024 Sep 30;19(9):e0311293. doi:10.1371/journal.pone.0311293

63.	Oliver BE, Nesbit RJ, McCloy R, Harvey K, Dodd HF. Parent perceived barriers and facilitators of children’s adventurous play in Britain: a framework analysis. BMC Public Health. 2022;22(1):1–17. doi:10.1186/s12889-022-13019-w

64.	Rutberg S, Henriksson M, Andersson M, Palstam A, Lindqvist AK. My Way to School Through a Camera Lens: Involving Children to Inform a Policy Recommendation on Active School Travel. Health Promot Pract. 2025 May;26(3):437–45. doi:10.1177/15248399241255376

65.	Sandseter EBH. Children’s Expressions of Exhilaration and Fear in Risky Play. Contemp Issues Early Child. 2009;10(2):92–106.

66.	Sandseter EBH. Restrictive safety or unsafe freedom? Norwegian ECEC practitioners’ perceptions and practices concerning children’s risky play. Child Care Pract. 2012;18(1):83–101. doi:10.1080/13575279.2011.621889

67.	Speldewinde C. ‘Don’t Pick, Don’t Lick’: Connecting Young Children’s Risky Play in Nature to Science Education in Australian Bush Kinders. Early Child Educ J. 2025 Apr;53(4):1133–44. doi:10.1007/s10643-024-01661-5

68.	Spencer RA, Joshi N, Branje K, Murray N, Kirk SF, Stone MR. Early childhood educator perceptions of risky play in an outdoor loose parts intervention. AIMS Public Health. 2021;8(2):213–28. doi:10.3934/publichealth.2021017

69.	Sterman J, Villeneuve M, Spencer G, Wyver S, Beetham KS, Naughton G, et al. Creating play opportunities on the school playground: Educator experiences of the Sydney playground project. Aust Occup Ther J. 2020;67(1):62–73. doi:10.1111/1440-1630.12624

70.	StGeorge JM, Goodwin JC, Fletcher RJ. Parents’ views of father-child rough-and-tumble play. J Child Fam Stud. 2018;27(5):1502–12. doi:10.1007/s10826-017-0993-0

71.	Traub M. Perspectives of Childcare Directors, Teachers, and Parents Regarding the Risks and Challenges of Nature Preschools [Dissertations and Doctoral Studies]. [Minneapolis, Minnesota]: Walden University; 2025.

72.	van Rooijen M, De Martelaer K, Lensvelt-Mulders G, van der Poel L, Cotterink M. “It Is Scary, but Then I Just Do It Anyway”: Children’s Experiences and Concerns about Risk and Challenge during Loose Parts Play. Int J Environ Res Public Health. 2023;20(22). doi:10.3390/ijerph20227032

73.	Weir H. Children’s autonomous mobility and their well-being. Wellbeing Space Soc. 2023;4. doi:10.1016/j.wss.2023.100134

74.	Zamani Z. ‘The woods is a more free space for children to be creative; their imagination kind of sparks out there’: exploring young children’s cognitive play opportunities in natural, manufactured and mixed outdoor preschool zones. J Adventure Educ Outdoor Learn. 2016 Apr 2;16(2):172–89. doi:10.1080/14729679.2015.1122538
