# Supplementary material for: Outdoor risky play and health-related outcomes in children aged 3–12 years: A mixed-methods systematic review
Source: PLoS One. 2026 Oct 26;21(10):e0347444. doi: 10.1371/journal.pone.0347444 (PMC13704885; doi:10.1371/journal.pone.0347444)
Supplement: Supplementary file 3 [file pone.0347444.s003.docx]

# **S3 Appendix: Qualitative Evidence Synthesis by Health Outcome**

| **Outcome category** | **Outcome** | **Exposure type** | **Qualitative studies reporting outcome** | **Illustrative quotes** |
| --- | --- | --- | --- | --- |
| PHYSICAL | Moderate to vigorous physical activity (MVPA) | General risky play | (Little, 2015, Hinchion et al., 2021, Oliver et al., 2022, Sandseter, 2009, Sandseter, 2012) | “*I jump on the rocks. It is called ‘Jumping house.’ We jump from one place and another friend jumps to another place, and the other friend jumps to the ‘two’ place. Then I jump, and other friend jumps to the ‘three’ place*.” (Child, outdoor preschool, US) (Zamani, 2016)  “*I like playing with the bikes mostly, and I just play with bikes, because sometimes I get to go fast, even though if someone is on the bike with me I can go fast*.” (Child, outdoor preschool, US) (Zamani, 2016)  “*Play with high speed includes swinging on swings or ropes hanging from trees, sliding down switchbacks or steep hills, sledging on the snow, running or skiing uncontrollably down steep hills, cycling at high speed both on level ground and downhill, and skating with high speed over icy surfaces in the winter.*” (Researcher, preschool, general risky play, Norway) (Sandseter, 2009)  “*She loves climbing, walls, trees the big climbing frame at the park. Running and jumping off things, trying to cartwheel at the beach.*” (Mother, general risky play, Britain) (Oliver et al., 2022)  “*With the exception of one teacher, who stated there had been no change in children’s activity, informants reported that access to materials on the playground generated more physically active play. Increases in activity were perceived not only in the form of greater aerobic exercise (e.g. running, jumping) but also in the opportunities presented for resistive exercise (e.g. lifting, pushing and pulling of large, heavy objects like hay-bales).*” (Researcher, loose parts play in primary school, Australia) (Bundy et al., 2009) |
|  |  | Risky play supportive environments | (Branje et al., 2021, Speldewinde, 2025, Bundy et al., 2009, Flannigan and Dietze, 2017, Spencer et al., 2021, Coe, 2017, Zamani, 2016, Farmer et al., 2017) |  |
|  |  | Independent mobility | (Weir, 2023, Rutberg et al., 2025) |  |
|  | Motor competence | General risky play: | (Hinchion et al., 2021; Little, 2015; Sandseter, 2009, 2012) | “*Some of them don’t have the balance or coordination that some of the older ones do, so they do start up a little more slowly and tentative then–but then when they go through these movements–you can really see that their coordination is improving, their balance is improving*.” (Educator, loose parts play in ECEC, Canada) (Branje et al., 2021)  “*I love the tire. I get in it and I hide under it…I also like to play jump over where I have to jump over it, it is a little hard*.” (Child, outdoor preschool, US) (Zamani, 2016)  **Pushing limits**: “*When sitting up on the top of the rock-climbing apparatus, Charlie started to tell Aoife what to do –‘get on the big bar, then put your foot here …’. Aoife then pushed her boundaries and tried to climb as high as Charlie*.” (Researcher and children aged 8 years, general risky play, Ireland) (Hinchion et al., 2021)  “*It is scary, but then I just do it anyway*.” (Child, loose parts play afterschool program, the Netherlands) (van Rooijen et al., 2023) |
|  |  | Risky play supportive environments | (Branje et al., 2021, McCormack et al., 2024, Spencer et al., 2021, Coe, 2017, Zamani, 2016, Farmer et al., 2017, van Rooijen et al., 2023, Speldewinde, 2025, Traub, 2025) |  |
|  |  | Rough and tumble play | (StGeorge et al., 2018) |  |
|  | Cardiovascular fitness | General risky play | (Little, 2015) | *“We have noticed that our children…we do those preparatory walks before going to Year 8 camp…that they’re much ﬁtter as a general group and we’re only, well, we’re only having to do two walks this year instead of four. The kids are much ﬁtter; like there’s generally no exceptions*.” (Primary School Leader, loose parts play in school, New Zealand) (Farmer et al., 2017)  “*Increases in activity were perceived not only in the form of greater aerobic exercise (e.g. running, jumping) but also in the opportunities presented for resistive exercise (e.g. lifting, pushing and pulling of large, heavy objects like hay-bales).*” (Researcher, loose parts play in primary school, Australia) (Bundy et al., 2009) |
|  |  | Risky play supportive environments | Bundy et al., 2017; Bundy et al., 2009; Farmer et al., 2017) |  |
|  |  | Independent mobility | (Rutberg et al., 2025) |  |
|  | Strength | General risky play | (Little, 2015) | “*Since I’ve seen her for the past year and a half, I’ve seen her physically become much stronger and much more coordinated. She was always coordinated, just growing into her own body and her own strength*.” (Teacher, Forest Kindergarten, Canada) (Coe, 2017)  “*There are some kids that can climb the tree on day one [and] there are other kids that really have to learn how to hang, how to put their leg up. It might take them a month to get to the first wrung of the tree and then they feel proud because they have done it. And then by the end of the year they might be able to go a bit higher. But they’re learning their limits. And then they are finally strong enough and adept enough to get up that tree, they’re less likely to get hurt because they worked their way up and kind of learned how to maneuver*.” (Director, Nature Preschool, US) (Traub, 2025) |
|  |  | Risky play supportive environments | (Bundy et al., 2009, Coe, 2017, Spencer et al., 2021, Traub, 2025) |  |
| MENTAL HEALTH & WELLBEING | Enjoyment | General risky play | (Hinchion et al., 2021, Bauer and Pike, 2023, Oliver et al., 2022, Sandseter, 2009, Sandseter, 2012) | “*It makes me happy, and it makes me less fearful.” “A bit scary, and yeah, it is fun!”* (Children, loose parts play afterschool program, the Netherlands) (van Rooijen et al., 2023)  “*Both fathers and mothers described RTP [rough and tumble] play as being moments of pure joy and fun between parent and child*.” (Researcher, rough and tumble play, Australia) (StGeorge et al., 2018)  *“Risky play is fun … It’s just a feeling of anticipation.*” (Child, general risky play, Canada) (Bauer and Pike, 2023)  “*Both fathers and mothers described RTP play as being moments of pure joy and fun between parent and child. However, there was also a view, mainly articulated by fathers, that child and father are strategizing about how to play and win the game. The father is planning to challenge the child while still giving them the opportunity of winning, and the child is thinking about how they can beat their father. Without this strategizing and motivation to win, some felt the play would not be fun.*” (Researcher, rough and tumble play, Australia) (StGeorge et al., 2018)  “*The children’s words show that they value risky play and that they experience fear and joy at the same time. ‘It makes me happy, and it makes me less fearful’. (H.) ‘A bit scary, and yeah, it is fun!’ (X.)*” (Researcher and children, loose parts play afterschool program, the Netherlands) (van Rooijen et al., 2023) |
|  |  | Risky play supportive environments | (Branje et al., 2021, Coe, 2017, Bundy et al., 2009, Spencer et al., 2021, van Rooijen et al., 2023, Zamani, 2016) |  |
|  |  | Independent mobility | (Rutberg et al., 2025) |  |
|  |  | Rough and tumble play | (StGeorge et al., 2018) |  |
|  | Self-confidence | General risky play | (Little, 2015, Oliver et al., 2022, Sandseter, 2012) | “*That we get more trust, we want to gain self-conﬁdence, we just want so much more*.” (Child, loose parts play afterschool program, the Netherlands) (van Rooijen et al., 2023)  “*I think the more risk they take, the more confidence they have*”. (Educator, loose parts play in ECEC, Canada) (Spencer et al., 2021)  “*Because they’re building up that inner, that inner, oh how would you say it, that resilience, that strength in feeling that they’re conﬁdent, they’re secure, they’re learning how to express themselves.*” (Mother, rough and tumble play, Australia) (StGeorge et al., 2018)  “*I think a certain level of risk is important to push you, to gain confidence, to expand your skill set and your knowledge base and so forth. I think I wouldn’t want him doing anything super dangerous, but moderate risk is pretty important, I think to push beyond your comfort zone*.” (Caregiver, loose parts play in community park, Canada) (McCormack et al., 2024) |
|  |  | Risky play supportive environments | (Spencer et al., 2021, McCormack et al., 2024, Flannigan and Dietze, 2017, Brussoni et al., 2017, Farmer et al., 2017, van Rooijen et al., 2023, Zamani, 2016, Traub, 2025) |  |
|  |  | Rough and tumble play | (StGeorge et al., 2018) |  |
|  | Self-efficacy | General risky play | (Hinchion et al., 2021, Sandseter, 2009, Sandseter, 2012) | “*Educators also spoke about how children’s progression of FMS coincided with increased confidence. As children repeated their movements, educators felt they became more confident that they could complete the movements, helping to improve their associated FMS [fundamental movement skills]*.” (Researcher, loose parts play in ECEC, Canada) (Branje et al., 2021)  “*The data indicated a strong desire to have opportunities to make their own decisions in risky play. With such opportunities, they feel free to push their boundaries and therefore dare to attempt something beyond their current skill level. By doing so, children feel more conﬁdent and trust their capabilities.”*  *‘That we think ourselves if it will succeed and have freedom to do this…Yes, then I am also proud of myself.’ (G.)*” (Researcher and child, loose parts play afterschool program, the Netherlands) (van Rooijen et al., 2023)  **Mastery experiences:** “*Similarly, ‘great speed’ changed over time and with mastery. Chloe explained during the ﬁnal discussion that ‘running up and down the ramps’ [at a skatepark] was challenging to begin with as it ‘took [her] a while’. She described ‘feeling really good’ after mastering it but sought more challenges for her enjoyment.*” (Researcher and child, general risky play, Ireland) (Hinchion et al., 2021) |
|  |  | Risky play supportive environments | (Branje et al., 2021, van Rooijen et al., 2023, Coe, 2017, Speldewinde, 2025) |  |
|  | Self-regulation | General risky play | (Little, 2015) | “*I think [the biggest advantage of a nature preschool is] for children to be who they are, to have the opportunity to move their bodies in ways that they need to move them, that when they move them in those ways, they [children] are more successful, very often at self-regulation.*” (Director, Nature Preschool, US) (Traub, 2025)  “*It’s how he copes. If he’s cranky, he’ll sit in the tree…he’ll swing around. He doesn’t function if he’s not climbing or playing outside.*” (Mother, general risky play, Australia) (Little, 2015)  **Manage fear and anxiety**: “*When the children* [3-5 years old] *at first built this little ramp, probably two and a half feet high off the ground. She* [child] *was very cautious and the little boy that first built it he was encouraging her, you can do it, you can do it, and he actually would walk along holding her hand until she became comfortable. And then it’s hard to tell but she’s running across it, she moved on from nervous to not cautious at all*.” (Educator, loose parts play in ECEC, Canada) (Branje et al., 2021) |
|  |  | Risky play supportive environments | (Flannigan and Dietze, 2017, McCormack et al., 2024, Brussoni et al., 2017, Branje et al., 2021, Traub, 2025) |  |
|  |  | Rough and tumble play | (StGeorge et al., 2018) |  |
|  | Resilience | General risky play | (Little, 2015, Oliver et al., 2022) | “*So engaged were they in [loose parts play] play that children who fell over were more likely to pick themselves up and continue playing rather than cry as they might previously have done*.” (Teacher, loose parts play in primary school, Australia) (Bundy et al., 2009)  “*Children enjoy risky behaviors and independent mobility in natural and mixed playgrounds…When you see kids, you worry that they might get hurt or they might fall down. But they really can control their bodies. A lot of kids fall down; they just hop back up and try again. They say, ‘No, I don’t want your help. I can do it myself.’”*(Teacher and child, outdoor preschool, US) (Zamani, 2016) |
|  |  | Risky play supportive environments | (Bundy et al., 2009, Zamani, 2016) |  |
|  |  | Rough and tumble play | (StGeorge et al., 2018) |  |
| AGGRESSION | Negative social behaviours | General risky play | Yes (Sandseter, 2009)  No (Sandseter, 2009) | “*The most prevalent negative behaviours observed were tattling, bossiness, testing of social limits, and exclusion of others*.” (Researcher, loose parts play in ECEC, Canada) (Flannigan and Dietze, 2017)  “*We have less teachers on duty now because the positive outcomes in terms of behaviour, kids being busy, there being less bullying, less conﬂict in the playground, means that we don’t run a time out space anymore for playground, you know, problems and we have less staﬀ on duty because the kids are much more engaged*.” (Primary School Leader, loose parts play in school, New Zealand) (Farmer et al., 2017)  “*The greatest concern about RTP* [rough and tumble play] *was the transfer of its antagonistic aspects to school settings. Some parents were clear that peer-to-peer RTP, without oversight from an adult, could end in crying, ﬁghting, and aggression when children do not know their own strength or want to dominate.*” (Researcher, rough and tumble play, Australia) (StGeorge et al., 2018) |
|  |  | Risky play supportive environments | Yes (Flannigan and Dietze, 2017, van Rooijen et al., 2023)  No (Bundy et al., 2009, Farmer et al., 2017, Bundy et al., 2017) |  |
|  |  | Rough and tumble play | Yes (StGeorge et al., 2018)  No (StGeorge et al., 2018) |  |
| SOCIAL | Social competence | General risky play | (Hinchion et al., 2021) | *“Children who did not play together before were now becoming playmates. They made plans together for what to do with the loose parts, which gave them enjoyment. ‘She didn’t belong to the group, and now we are playing together*.’” (Researcher and child, loose parts play afterschool program, the Netherlands) (van Rooijen et al., 2023)  *“They [Children] also pointed out that being independent made it easier to make after-school plans with friends. Tilde: ‘And it’s easier if you want to hang out with a friend or someone else after school if you cycle or walk.’ Olivia: ‘Because when I want to stay on the football field, I usually cycle to school.’”* (Children and Researcher, Independent mobility, Sweden) (Rutberg et al., 2025)  “*Data from the tour, photographs and pictures, showed that risky play was usually a social form of play, most often done together with friends and siblings, and without adult surveillance. When the children played together, they encouraged each other to test their boundaries further, which also augmented the risky play potential.*” (Researcher, general risky play, Ireland) (Hinchion et al., 2021) |
|  |  | Risky play supportive environments | (Brussoni et al., 2017, Bundy et al., 2009, Flannigan and Dietze, 2017, Sterman et al., 2020, van Rooijen et al., 2023, Bundy et al., 2017) |  |
|  |  | Independent mobility | (Weir, 2023, Rutberg et al., 2025) |  |
|  |  | Rough and tumble play | (StGeorge et al., 2018) |  |
|  | Cooperation | General risky play | (Hinchion et al., 2021) | “*Most of the play episodes included children working toward achieving common goals, such as finding materials for their home, chasing the bad guy, or making food. Each child would have a role assigned and they would work together to execute the play episode.*” (Researcher, loose parts play in ECEC, Canada) (Flannigan and Dietze, 2017)  “*In the natural playground, children talk a lot, and they even say, ‘We are in the same team’ or ‘Let’s work together,’ even [when they are] just picking up a stick or building something that they all can use.*” (Researcher and child, outdoor preschool, US) (Zamani, 2016)  “*When I first came, there was no cooperative play, but now they're working together in role play games, using their imaginations and building block towers together*.” (Special education teacher, loose parts play in primary school, Australia) (Sterman et al., 2020) |
|  |  | Risky play supportive environments | (Branje et al., 2021, Bundy et al., 2009, Farmer et al., 2017, Flannigan and Dietze, 2017, Sterman et al., 2020, Zamani, 2016, Coe, 2017) |  |
|  | Communication skills | Risky play supportive environments | (Branje et al., 2021, Flannigan and Dietze, 2017, Bundy et al., 2009, van Rooijen et al., 2023) | *“Children engaged in a number of common verbal patterns: imitation, repetition, stating the theme, expressions of happiness or excitement… and using socially inclusive phrases of ‘we’ or ‘ours,’ such as ‘we have to go get the bad guys,’ ‘this tree stump is ours and we must protect it’.”* (Researcher and children aged 4-5 years, loose parts play in ECEC, Canada) (Flannigan and Dietze, 2017) |
|  | Social problem solving | General risky play | (Bauer et al., 2024) | “*I think it* [risky outdoor play] *makes them be better problem solvers...They need to, you know, work as a team with their friends. And you know, if a bully comes along and pushes [my daughters] off the swing, they have to figure out how to deal with it cause I can’t be there with them all the time.”* (Mother, general risky play, Canada) (Bauer et al., 2024) |
|  |  | Risky play environments | (Flannigan and Dietze, 2017, van Rooijen et al., 2023) |  |
| COGNITIVE | Creativity | General risky play | (Hinchion et al., 2021) | “*We always play astronaut; the parasol is then the satellite and the barrel is the rocket which rolls down the mountain. I then go after the barrel with the buggy or run with the parasol after it to have enough reach*.” (Child, loose parts play afterschool program, the Netherlands) (van Rooijen et al., 2023)  “*Children identiﬁed that risky play was associated with fun combined with scary experiences concerning heights, speed and use of exciting tools. These play events were often accompanied by imaginative play. For example, in the focus group, Dan was keen to depict how he climbs trees at home to be a spy who jumps out and shoots people*.” (Researcher and child, general risky play, Ireland) (Hinchion et al., 2021)  “*Children were reported to have made inventive use of the materials’ potential for construction (e.g. building a ‘pyramid’…creating spontaneous rule-based games (e.g. who was allowed to climb on a built structure); creating friendly competitive games (e.g. tyre-rolling contests); testing physical prowess (e.g. ‘balancing’ on tyres or walking along planks); and creating highly imaginative play (e.g. sitting in tyres ‘pretending [to be] on some Caribbean cruise’)*." (Researcher and teachers, loose parts play in primary school, Australia) (Bundy et al., 2009) |
|  |  | Risky play supportive environments | (Branje et al., 2021, Brussoni et al., 2017, McCormack et al., 2024, Bundy et al., 2009, Farmer et al., 2017, Flannigan and Dietze, 2017, Sterman et al., 2020, van Rooijen et al., 2023, Zamani, 2016, Bundy et al., 2017, Traub, 2025) |  |
|  | Perceptual awareness | Risky play supportive environments | (Coe, 2017, Zamani, 2016, Branje et al., 2021) | “*When children play on playground structures, it is very much predictable. The steps are always a certain measure of height…But when they are in the woods, they climb up on the logs; and this log might be diﬀerent from that log; and it might roll over; and it might crunch. They really learn their bodies out there.*” (Educator, outdoor preschool, US) (Zamani, 2016) |
|  |  | Rough and tumble play | (StGeorge et al., 2018) |  |
|  | Cognitive problem solving | General risky play | (Bauer et al., 2024, Oliver et al., 2022, Little, 2015) | “*One educator described a specific photograph where several children were using the loose parts as an obstacle course. One child, however, wanted to go in the opposite direction of the other children…‘They were trying to figure out how to get around each other because they were, like, the rest of them were kind of heading this way, and he was trying to go the opposite way as everybody else, so he was trying to figure out how to get around here without falling off’*.” (Researcher and educator, loose parts play in ECEC, Canada) (Branje et al. 2021) |
|  |  | Risky play supportive environments | (Branje et al., 2021, Brussoni et al., 2017, Flannigan and Dietze, 2017, Spencer et al., 2021) |  |
|  | Language development | Risky play environments | (Branje et al., 2021, Flannigan and Dietze, 2017, Bundy et al., 2017) | *“We have a lot of children at this site that are learning English it’s been remarkable like how fast these children are learning English because they’re trying to help with building these things and so they’re learning the words for it and they’ll say ‘can I help’ and then listening to all the cues that they’re giving each other and catching on to them until they’re using them.*” (Educator, loose parts play in ECEC, Canada) (Branje et al., 2021) |
|  | Experiential learning | Risky play supportive environments | (Speldewinde, 2025, Zamani, 2016, Traub, 2025) | *“We moved over to Poppy* [child] *and Bernie* [educator] *asked ‘why do you keep falling down, what makes things fall down from trees?’ Poppy replied ‘I think it is gravity.’ Bernie, continued to ask Poppy about gravity then asked Poppy about what she had learned about climbing. Poppy said she had to hold on tightly but wasn’t scared of falling off because ‘the ground was nice and soft.’”* (Child, educator and researcher, Bush Kindergarten, Australia) (Speldewinde, 2025)  “*Science is a huge part of everything they do. They do this dirt hill, that is the kids favorite thing, [a] massive dirt hill…they are always learning about bugs, different types of bugs…frog life cycles, they [the children] caught tadpoles and studied watching them grow*.” (Parent, Nature Preschool, US) (Traub, 2025) |
| RISK COMPETENCE | Risk willingness | General risky play | (Bauer and Pike, 2023, Hinchion et al., 2021, Sandseter, 2009) | *“Marianne eagerly took on challenges that pushed her physical and mental boundaries. According to Teacher Jane…‘She likes to climb. Whatever it is. Rocks, trees. She likes to challenge herself but is very, very conscious of and is very aware of her body and her limitations. She’s a risk-taker in a safe manner.’”* (Research and teacher, Forest Kindergarten, Canada) (Coe, 2017)  “*It [loose parts play] gives them the opportunity to scale their risk, depending on what they’re comfortable with.*” (Educator, loose parts play in ECEC, Canada) (Spencer et al., 2021) |
|  |  | Risky play supportive environments | (Coe, 2017, Flannigan and Dietze, 2017, Spencer et al., 2021, van Rooijen et al., 2023) |  |
|  |  | Independent mobility | (Weir, 2023) |  |
|  | Risk assessment | General risky play | (Bauer et al., 2024, Bauer and Pike, 2023, Little, 2015, Oliver et al., 2022, Sandseter, 2009) | “*It* [risky play] *works on their judgment skills. As an adult, we’ve gone through that experience and had a chance to rationalize and think about it and use our judgment. Kids need those experiences*.” (Caregiver, loose parts play in community park, Canada) (McCormack et al., 2024)  *“… If someone* [child] *wants to climb a tree, which they’re most welcome to do, they make sure that one of the grown-ups is nearby [and] able to watch…they also then check all of the ground around for anything they may hurt themselves on…And then if there are branches low enough down, they try to wiggle them and see if they’re going to be strong enough for their weight.”* (Teacher, Forest Kindergarten, Canada) (Coe, 2017)  “*Giving them the room to assess their own limits will serve them well as an adult. They definitely need the space to be able to look at the situation and make a safety assessment for themselves … a couple of times [my youngest son] was climbing things and I was watching and I was thinking ‘I’m not going to jump in, I’m not going to jump in’, and I have to talk myself down. But then you see the light switch and look and assess and change his behavior and I think that translates directly into adulthood where he will look at a behavior that he is choosing and having practiced, even in play-base, he will assess ‘oh this is probably not the wisest course, I should self-correct’.*” (Mother, general risky play, Canada) (Bauer et al., 2024)  “*Encouraging kids to trust their own bodies, so that might mean that they’re climbing a tree and does that branch feel strong enough to hold you. Do you feel safe that high? Do you feel like you can get back down? So encouraging them to take risks, in a sense to learn what they [the children] are most comfortable with.*” (Teacher, nature preschool, US) (Traub, 2025) |
|  |  | Risky play supportive environments | (Branje et al., 2021, McCormack et al., 2024, Coe, 2017, Higginbottom et al., 2023, Spencer et al., 2021, Flannigan and Dietze, 2017, Sterman et al., 2020, Traub, 2025) |  |
|  | Risk handling | General risky play | (Bauer et al., 2024, Oliver et al., 2022, Bauer and Pike, 2023, Hinchion et al., 2021, Little, 2015) | “*The educators recalled how children’s movement and manipulation of loose parts sometimes created an element of risk (e.g., carrying heavy loose parts, climbing and balancing at great height). In order to navigate these risks, children needed to move their bodies in different ways, which demanded various FMS*.” (Researcher, loose parts play in ECEC, Canada) (Branje et al., 2021)  “*Many observations illustrated children’s risk-taking behaviours. For example, children were observed climbing trees, swinging fast on a tree-rope, climbing on a pile of sticks, and walking across a tightrope. The behaviours revealed ways in which the children managed their own risks separate from teacher influence. The children rarely rushed into a risky situation without testing or questioning the action or environment. Oftentimes they would take sequential steps as part of completing an entire risky movement*”. (Researcher, loose parts play in ECEC, Canada) (Flannigan and Dietze, 2017)  “*Development of risk-competence Interviews at pre- and post-intervention indicated changes in children’s vocabulary associated with risk-competence (Table 3). This includes children’s articulation of additional safety measures during risky play, such as wearing gloves, or seeking adult supervision, when asked the question ‘How do you make play safe?’ Post-intervention, two observations captured through video footage, reﬂected the children’s developing risk-competence. In observation one, despite child A pointing to child B’s sockclad feet, stating ‘you [have] not got your socks off’, child B slipped from a balance board. Following the incident, in conversation with an educator, child B was able to articulate that he should have removed his socks, stating that it is ‘slippery on socks’. Educator –‘what did you do after that?’ Child B- ‘I climbed without socks on’. Educator –‘and why did that make it better?’ Child B –‘cause my feet sticked to it’. This reﬂected Child B’s growing understanding around controlling future hazards for risky play and language associated with maintaining safety during risky-play experiences.*” (Researcher, educator, child, loose parts play in ECEC, Australia) (Higginbottom et al., 2023) |
|  |  | Risky play supportive environments | (Branje et al., 2021, Speldewinde, 2025, Bundy et al., 2009, Coe, 2017, Farmer et al., 2017, Flannigan and Dietze, 2017, Higginbottom et al., 2023, Spencer et al., 2021, Traub, 2025) |  |
|  | Antifragility | General risky play | (Bauer et al., 2024, Little, 2015, Oliver et al., 2022, Sandseter, 2012) | “*Giving them the opportunities to learn from their mistakes cause they are going to make mistakes and chances are they are going to fall, and chances are they might hurt themselves, but they’re also going to learn the next time, I’m not going to do that so how can I make it better so that doesn’t happen again*.” (Educator, loose parts play in ECEC, Canada) (Branje et al., 2021)  “*I think nearly every aspect of learning involves risk. Not necessarily physical risk but social risk or emotional risk. Like learning to speak, learning new words, making new friends, all of that, there’s potential for you to get hurt in some way.*” (Mother, general risky play, Australia) (Little, 2015)  “*I do like them to take some risks because that’s how they learn, right, that if they fall, they need to learn how to get back up. So, I do like that, so that they’re not always protected and not being exposed, you know what I’m saying, but at the same time, you don’t want them to get hurt too badly*.” (Caregiver, loose parts play in community park, Canada) (McCormack et al., 2024) |
|  |  | Risky play supportive environments | (Branje et al., 2021, McCormack et al., 2024, Bundy et al., 2009, Spencer et al., 2021, van Rooijen et al., 2023, Zamani, 2016) |  |
|  |  | Independent mobility | (Weir, 2023) |  |
|  |  | Rough and tumble play | (StGeorge et al., 2018) |  |
|  | Injuries | General risky play | Greater risk of: (Oliver et al., 2022, Bauer et al., 2024)  Few/er or minor: (Bauer et al., 2024, Bauer and Pike, 2023, Little, 2015, Sandseter, 2012) | *“All of the practitioners in this study indicated that there were very few injuries in their kindergarten; if injuries happened, they were mostly minor such as bruises, scratches, small cuts and wounds, and a few broken bones.* (Researcher, preschool, general risky play, Norway) (Sandseter, 2012)  *“Worrying that the risks they take may result in injury.”* (Mother, general risky play, Britain) (Oliver et al., 2022)  *“Fear of them getting seriously hurt.”* (Father, general risky play, Britain) (Oliver et al., 2022)  *“They [Educators] perceived that children experienced less stress, boredom and injury.”* (Researcher, loose parts play in ECEC, Canada) (Brussoni et al., 2017)  *“Some parents noted that that even though the play could end with injury or upset, this was not problematic and it could be considered a healthy part of growing up. Further, some parents highlighted that although there is a risk of tears and injury (which is more likely in sibling or peer play), these outcomes usually do not eventuate in father–child play.”* (Researcher, rough and tumble play, Australia) (StGeorge et al., 2018)  *“Most of the injuries in the kindergartens, according to these practitioners [Educators], were unpredictable and unforeseen incidents in places other than the most obvious risky features. This could be such as children running on flat ground and falling in an unfortunate way, rather than severe falls from great heights”.* (Researcher, preschool, general risky play, Norway) (Sandseter, 2012) |
|  |  | Risky play supportive environments | Greater risk of: (Bundy et al., 2009, McCormack et al., 2024, Traub, 2025)  Few/er or minor: (Brussoni et al., 2017, Bundy et al., 2009, Higginbottom et al., 2023, Spencer et al., 2021, Bundy et al., 2017, Traub, 2025, McCormack et al., 2024) |  |
|  |  | Rough and tumble play | Few/er or minor: (StGeorge et al., 2018) |  |

# Table Key

ECEC = early childhood education and care; US = United States

# References

BAUER, M. E. E., GILES, A. R. & BRUSSONI, M. 2024. “I’ve Seen What Evil Men Do”: Military Mothering and Children’s Outdoor Risky Play. *Leisure Sciences,* 46**,** 150-166.

BAUER, M. E. E. & PIKE, I. 2023. What children's perspectives on safe and dangerous outdoor play can tell us about their risk-seeking and injury experiences: 'You don't feel right doing the thing that got you hurt'. *Inj Prev,* 29**,** 367-370.

BRANJE, K., STEVENS, D., HOBSON, H., KIRK, S. & STONE, M. 2021. Impact of an outdoor loose parts intervention on Nova Scotia preschoolers' fundamental movement skills: a multi-methods randomized controlled trial. *AIMS Public Health,* 9**,** 194.

BRUSSONI, M., ISHIKAWA, T., BRUNELLE, S. & HERRINGTON, S. 2017. Landscapes for play: Effects of an intervention to promote nature-based risky play in early childhood centres. *Journal of Environmental Psychology,* 54**,** 139-150.

BUNDY, A., ENGELEN, L., WYVER, S., TRANTER, P., RAGEN, J., BAUMAN, A., BAUR, L., SCHILLER, W., SIMPSON, J. M., NIEHUES, A. N., PERRY, G., JESSUP, G. & NAUGHTON, G. 2017. Sydney Playground Project: A Cluster-Randomized Trial to Increase Physical Activity, Play, and Social Skills. *Journal of School Health,* 87**,** 751-759.

BUNDY, A. C., LUCKETT, T., TRANTER, P. J., NAUGHTON, G. A., WYVER, S. R., RAGEN, J. & SPIES, G. 2009. The risk is that there is ‘no risk’: a simple, innovative intervention to increase children’s activity levels. *International Journal of Early Years Education,* 17**,** 33-45.

COE, H. A. 2017. Embracing risk in the Canadian woodlands: Four children’s risky play and risk-taking experiences in a Canadian Forest Kindergarten. *Journal of Early Childhood Research,* 15**,** 374-388.

FARMER, V. L., FITZGERALD, R. P., WILLIAMS, S. M., MANN, J. I., SCHOFIELD, G., MCPHEE, J. C. & TAYLOR, R. W. 2017. What did schools experience from participating in a randomised controlled study (PLAY) that prioritised risk and challenge in active play for children while at school? *Journal of Adventure Education and Outdoor Learning,* 17**,** 239-257.

FLANNIGAN, C. & DIETZE, B. 2017. Children, outdoor play, and loose parts. *Journal of Childhood Studies***,** 53-60.

HIGGINBOTTOM, K., NEWMAN, L., WEST-SOOBY, K. & WOOD, A. 2023. Intentional teaching for risky play: Practitioner researchers move beyond their comfort zones. *Australasian Journal of Early Childhood,* 48**,** 18-33.

HINCHION, S., MCAULIFFE, E. & LYNCH, H. 2021. Fraught with frights or full of fun: perspectives of risky play among six-to-eight-year olds. *European Early Childhood Education Research Journal***,** 1-19.

LITTLE, H. 2015. Mothers’ beliefs about risk and risk-taking in children’s outdoor play. *Journal of Adventure Education and Outdoor Learning,* 15**,** 24-39.

MCCORMACK, G. R., NAISH, C., PETERSEN, J., GHONEIM, D. & DOYLE-BAKER, P. K. 2024. It is child's play: Caregiver and playworker perspectives on a community park-based unstructured play program. *PLoS One,* 19**,** e0311293.

OLIVER, B. E., NESBIT, R. J., MCCLOY, R., HARVEY, K. & DODD, H. F. 2022. Parent perceived barriers and facilitators of children’s adventurous play in Britain: a framework analysis. *BMC Public Health,* 22**,** 636.

RUTBERG, S., HENRIKSSON, M., ANDERSSON, M., PALSTAM, A. & LINDQVIST, A. K. 2025. My Way to School Through a Camera Lens: Involving Children to Inform a Policy Recommendation on Active School Travel. *Health Promot Pract,* 26**,** 437-445.

SANDSETER, E. B. H. 2009. Children's expressions of exhilaration and fear in risky play. *Contemporary Issues in Early Childhood,* 10**,** 92-106.

SANDSETER, E. B. H. 2012. Restrictive safety or unsafe freedom? Norwegian ECEC practitioners' perceptions and practices concerning children's risky play. *Child Care in Practice,* 18**,** 83-101.

SPELDEWINDE, C. 2025. ‘Don’t Pick, Don’t Lick’: Connecting Young Children’s Risky Play in Nature to Science Education in Australian Bush Kinders. *Early Childhood Education Journal,* 53**,** 1133-1144.

SPENCER, R. A., JOSHI, N., BRANJE, K., MURRAY, N., KIRK, S. F. & STONE, M. R. 2021. Early childhood educator perceptions of risky play in an outdoor loose parts intervention. *AIMS public health,* 8**,** 213.

STERMAN, J., VILLENEUVE, M., SPENCER, G., WYVER, S., BEETHAM, K. S., NAUGHTON, G., TRANTER, P., RAGEN, J. & BUNDY, A. 2020. Creating play opportunities on the school playground: Educator experiences of the Sydney playground project. *Australian occupational therapy journal,* 67**,** 62-73.

STGEORGE, J. M., GOODWIN, J. C. & FLETCHER, R. J. 2018. Parents’ views of father–child rough-and-tumble play. *Journal of Child and Family Studies,* 27**,** 1502-1512.

TRAUB, M. 2025. *Perspectives of Childcare Directors, Teachers, and Parents Regarding the Risks and Challenges of Nature Preschools.* Walden University.

VAN ROOIJEN, M., DE MARTELAER, K., LENSVELT-MULDERS, G., VAN DER POEL, L. & COTTERINK, M. 2023. “It Is Scary, but Then I Just Do It Anyway”: Children’s Experiences and Concerns about Risk and Challenge during Loose Parts Play. *International journal of environmental research and public health,* 20**,** 7032.

WEIR, H. 2023. Children's autonomous mobility and their well-being. *Wellbeing, Space and Society,* 4**,** 100134.

ZAMANI, Z. 2016. ‘The woods is a more free space for children to be creative; their imagination kind of sparks out there’: Exploring young children’s cognitive play opportunities in natural, manufactured and mixed outdoor preschool zones. *Journal of adventure education and outdoor learning,* 16**,** 172-189.
