# Supplementary material for: Outdoor risky play and health-related outcomes in children aged 3–12 years: A mixed-methods systematic review
Source: PLoS One. 2026 Oct 26;21(10):e0347444. doi: 10.1371/journal.pone.0347444 (PMC13704885; doi:10.1371/journal.pone.0347444)
Supplement: Supplementary file 4 [file pone.0347444.s004.docx]

# **S4 Appendix: Synthesized quantitative and qualitative results**

Table S4-1: Health outcome categories derived from the quantitative narrative synthesis and the qualitative framework synthesis listed and matched.

| **Quantitative Health Outcomes (List)** | **Synthesis Matching** | **Qualitative Health Outcomes (List)** |
| --- | --- | --- |
| **Aggression** | **Aggression** | **Aggression and Behavioural Issues** |
| Aggression |  | Less aggressive behaviour |
| Dominance |  | More competition for play materials |
| Maladjustment (aggression) |  | Negative social behaviours |
| Physical aggression |  | Play fighting turns into real fighting |
| Playful function |  |  |
| Proactive aggression bullying |  |  |
| Proactive instrumental aggression |  |  |
| Reactive aggression bullying |  |  |
| Reactive instrumental aggression |  |  |
| Reactive physical aggression |  |  |
| **Cognitive** | **Cognitive** | **Cognitive / Academic Outcomes** |
| Executive Function |  | Creativity |
| Concentration |  | Executive Function |
| Distractible |  | Perceptual awareness |
| Executive functioning |  |  |
| Involvement |  |  |
| Working memory |  |  |
| **Injury and Risk Behaviours** | **Risk Competence and Injury** | **Injury and Risk Behaviours** |
| ***Injury*** | ***Injury*** | ***Injury*** |
| Experience of injury | ***Risk Perception & Antifragility*** | Fewer injuries |
| Injury occurrence |  | Greater injury risk |
| Injury severity |  | Injuries - fewer from risky play |
| Risk factors for non-fatal drowning incidence |  | Injuries - fewer injuries from risky play |
|  |  | Injuries - risky vs dangerous play |
|  |  | More injuries from risky play |
| ***Risk Perception*** |  | ***Risk Competence*** |
| Observed risk competence skills |  | Adjust behaviour in response to risk |
| Proportion of correct answers on risk perception test |  | Antifragility |
| Reaction time on risk perception test |  | Children don't understand own physical limits, |
|  |  | Peer monitoring, |
|  |  | Perceive and assess risk |
|  |  | Problem solving |
|  |  | Risk competence - |
|  |  | Risk competence - Adjust behaviour in response to risk |
|  |  | Risk competence - children don't understand their own physical limits |
|  |  | Risk competence - perceive and assess risk |
|  |  | Risk competence - Problem solving |
|  |  | Risk competence - safety checks, |
|  |  | Risk competence -Problem solving, |
|  |  | Risk competence -Self monitoring, |
|  |  | Risk competence -Self monitoring, self-perception, |
|  |  | Risk competnece - Peer-monitoring |
|  |  | Risk comptenece - undertsand own physical limits |
|  |  | Safety checks, |
|  |  | Self assessment |
|  |  | Self assessment, Understand physical limits, |
|  |  | Self monitoring |
|  |  | Self-monitoring safety behaviours |
|  |  | Understand physical limits |
| **Mental Health and Well-being** | **Mental Health and Well-being** | **Mental and emotional wellbeing** |
| ***Emotion regulation*** |  | ***Anxiety*** |
| Emotion dysregulation |  | Manage fear & anxiety |
| Emotion regulation |  | Parent anxiety |
| Internalising and externalising problems |  | ***Resilience*** |
| Negative emotion expressiveness |  | Resilience |
| Positive emotion expressiveness |  | ***Self-confidence*** |
| Self-control ability |  | Confidence |
| Socioemotional strengths and difficulties |  | Confidence - Mastery experiences |
| Venting |  | Self-perception |
| ***Self-confidence*** |  | ***Self-regulation*** |
| Self-esteem |  | Self-regulation |
| ***Happiness and Well-being*** |  | Self-regulation - emotions |
| Affect |  | ***Wellbeing*** |
| Childhood anxiety symptoms |  | Wellbeing, enjoyment |
| Happy at school |  | Wellbeing, happiness and enjoyment |
| Wellbeing |  |  |
| Wellbeing- happiness |  |  |
| **Physical** | **Physical** | **Physical** |
| ***Anthropometric*** |  | ***Cardiovascular*** |
| BMI z-score |  | CV fitness |
| Waist circumference |  | ***Coordination*** |
| Waist to height ratio |  | Complex movements |
| ***Cardiovascular*** |  | FMS development |
| Cardiorespiratory fitness |  | FMS development - coordination |
| ***Motor Competence*** |  | FMS development - risk taking testing limits |
| Balance |  | ***Physical Activity*** |
| Fundamental motor skills |  | Vigorous PA |
| Fundamental movement skills |  | ***Strength*** |
| Locomotor skills |  | Muscular strength |
| Motor control |  |  |
| Object control skills |  |  |
| ***Physical Activity*** |  |  |
| Active transport |  |  |
| Activity level |  |  |
| Physical activity |  |  |
| Sedentary behaviour |  |  |
| **Social** | **Social** | **Social** |
| ***Peer Relationships*** |  | ***Peer Relationships*** |
| Active exclusion |  | Cooperation |
| Excluded during playtime |  | ***Social Competence*** |
| Peer acceptance |  | communication skills |
| Play and social interactions |  | Connection to place and community |
| Popularity |  | Negative social behaviours |
| Sociable/Friendly |  | Pro-social behaviours |
| Social acceptance |  | Pro-social behaviours - friendly, helpful |
| Social preference & social impact |  | Social competence |
| Sociometric status |  | Social competence - relationships |
| Sociometry (likes most, likes least) |  | Social problem solving |
| Solitary passive withdrawal reticence |  |  |
| Victim status |  |  |
| Victimisation |  |  |
| Withdrawal |  |  |
| ***Social Communication*** |  |  |
| Assertiveness |  |  |
| ***Social Competence*** |  |  |
| Adjustment (social behaviours) |  |  |
| Antisocial Behaviour |  |  |
| Conflict sensitivity |  |  |
| Outcome expectations (unfriendly-assertive strategy; friendly-assertive strategy) |  |  |
| Peer competence |  |  |
| Prosocial behaviour |  |  |
| Social affiliation |  |  |
| Social behaviours |  |  |
| Social cognition |  |  |
| Social competence |  |  |
| Social problem solving |  |  |
| Social problem-solving flexibility |  |  |
| Temperament |  |  |

Table S4-2: Graded strength and directionality of evidence for each dataset (quantitative and qualitative) and overall (synthesized).

| **Health Outcome Category** | **Exposure Type** | **Quantitative Evidence (n)** | **Quantitative Quality of Evidence (%)** | **Quantitative Relationship** | **Qualitative Evidence (n)** | **Qualitative Quality of evidence (%)** | **Qualitative Relationship** | **Synthesis (Overall Relationship)** |
| --- | --- | --- | --- | --- | --- | --- | --- | --- |
| Aggression | Disappear/get lost (IM) | n/a | n/a | Insufficient | n/a | n/a | Insufficient | Insufficient |
| Aggression | General risky play | n/a | n/a | Insufficient | * (1) | * 100% high | Insufficient | Insufficient |
| Aggression | Risky play supportive environments | + (1) | + 100% moderate | Insufficient | + (3); - (2) | + 33% low; 33% moderate; 33% high - 50% moderate; 50% high | Mixed | Insufficient |
| Aggression | Rough-and-tumble | - (12) | - 75% low; 25% moderate | Moderate Negative | * (1) | * 100% moderate | Insufficient | Low Negative |
| Cognitive | Disappear/get lost (IM) | n/a | n/a | Insufficient | n/a | n/a | Insufficient | Insufficient |
| Cognitive | General risky play | + (1) | + 100% low | Insufficient | + (4) | + 100% high | Moderate Positive | Low Positive |
| Cognitive | Risky play supportive environments | n/a | n/a | Insufficient | + (14) | + 21% low; 43% moderate; 36% high | High Positive | Moderate Positive |
| Cognitive | Rough-and-tumble | + (1); * (1); - (2) | + 100% moderate * 100% moderate - 100& low | Mixed | + (1) | + 100% moderate | Insufficient | Insufficient |
| Injury | Disappear/get lost (IM) | n/a | n/a | Insufficient | n/a | n/a | Insufficient | Insufficient |
| Injury | General risky play | - (1) | - 100% moderate | Insufficient | + (4); - (1) | + 25% moderate; 75% high | Low Positive | Low Positive |
| Injury | Risky play supportive environments | n/a | n/a | Insufficient | + (4); * (2); - (1) | + 75% moderate; 25% high * 100% low - 100% high | Low Positive | Low Positive |
| Injury | Rough-and-tumble | n/a | n/a | Insufficient | + (1) | + 100% moderate | Insufficient | Insufficient |
| Mental Health & Well-being | Disappear/get lost (IM) | + (1) - (1) | + 100% moderate - 100% moderate | Mixed | + (1) | + 100% high | Insufficient | Insufficient |
| Mental Health & Well-being | General risky play | + (4) | + 25% low; 25% moderate; 50% high | Moderate Positive | + (6) | + 17% moderate; 83% high | High Positive | High Positive |
| Mental Health & Well-being | Risky play supportive environments | + (2) | + 50% low; 50% moderate | Low Positive | + (12) | + 25% low; 33% moderate; 42% high | High Positive | High Positive |
| Mental Health & Well-being | Rough-and-tumble | + (2); - (3) | + 50% moderate; 50% low - 100% moderate | Mixed | + (1) | + 100% moderate | Insufficient | Insufficient |
| Physical | Disappear/get lost (IM) | + (13); * (1) | + 15% low; 69% moderate; 15% high  * 100% moderate | High Positive | + (2) | + 100% high | Low Positive | High Positive |
| Physical | General risky play | + (4); * (3); - (3) | + 25% low; 75% moderate * 33% low; 66% moderate - 33% low; 66% moderate | Mixed | + (5) | + 100% high | High Positive | Moderate Positive |
| Physical | Risky play supportive environments | + (1); * (1) | + 100% high * 100% low | Mixed | + (12) | + 25% low; 33% moderate; 42% high | High Positive | Moderate Positive |
| Physical | Rough-and-tumble | n/a | n/a | Insufficient | + (1) | + 100% moderate | Insufficient | Insufficient |
| Risk Competence | Disappear/get lost (IM) | n/a | n/a | Insufficient | + (1) | + 100% high | Insufficient | Insufficient |
| Risk Competence | General risky play | + (1) | + 100% low | Insufficient | + (7) | + 14% moderate; 86% high | High Positive | Moderate Positive |
| Risk Competence | Risky play supportive environments | n/a | n/a | Insufficient | + (13) | + 23% low; 31% moderate; 46% high | High Positive | Moderate Positive |
| Risk Competence | Rough-and-tumble | n/a | n/a | Insufficient | + (1) | + 100% moderate | Insufficient | Insufficient |
| Social | Disappear/get lost (IM) | n/a | n/a | Insufficient | + (2) | + 100% high | Low Positive | Low Positive |
| Social | General risky play | + (2) | + 100% low | Low Positive | + (2) | + 100% high | Low Positive | High Positive |
| Social | Risky play supportive environments | + (2); * (1) | + 50% moderate; 50% high * 100% moderate | Mixed | + (10) | + 20% low; 50% moderate; 30% high | High Positive | Moderate Positive |
| Social | Rough-and-tumble | + (4); * (5); - (2) | + 50% low; 50% moderate * 20% low; 80% moderate - 50% low; 50% moderate | Mixed | + (1) | + 100% moderate | Insufficient | Insufficient |
